# Photothermal Nanomaterials: A Powerful Light-to-Heat Converter

**DOI:** 10.1021/acs.chemrev.3c00159

**Published:** 2023-05-03

**Authors:** Ximin Cui, Qifeng Ruan, Xiaolu Zhuo, Xinyue Xia, Jingtian Hu, Runfang Fu, Yang Li, Jianfang Wang, Hongxing Xu

**Affiliations:** ∇ State Key Laboratory of Radio Frequency Heterogeneous Integration, College of Electronics and Information Engineering, Shenzhen University, Shenzhen 518060, China; ‡ Department of Physics, The Chinese University of Hong Kong, Shatin, Hong Kong SAR 999077, China; § Ministry of Industry and Information Technology Key Lab of Micro-Nano Optoelectronic Information System & Guangdong Provincial Key Laboratory of Semiconductor Optoelectronic Materials and Intelligent Photonic Systems, Harbin Institute of Technology, Shenzhen 518055, China; ∥ Guangdong Provincial Key Lab of Optoelectronic Materials and Chips, School of Science and Engineering, The Chinese University of Hong Kong (Shenzhen), Shenzhen 518172, China; ⊥ School of Physics and Technology and School of Microelectronics, Wuhan University, Wuhan 430072, Hubei, China; £ Henan Academy of Sciences, Zhengzhou 450046, Henan, China; ◆ Wuhan Institute of Quantum Technology, Wuhan 430205, Hubei, China

## Abstract

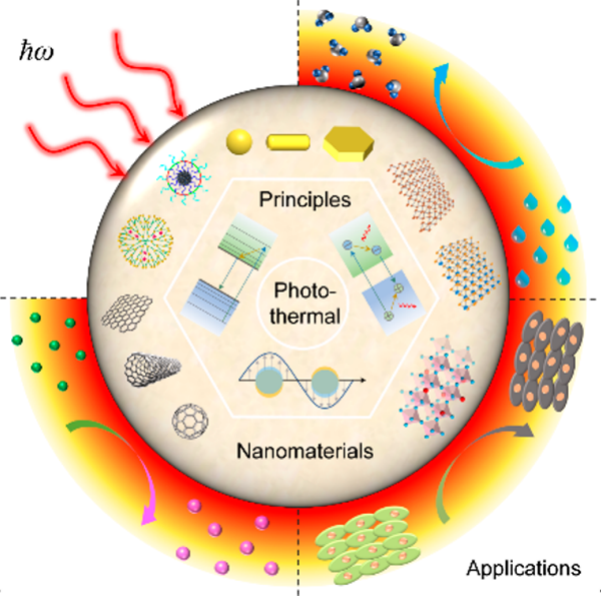

All forms of energy follow the law of conservation of energy, by which they can be neither created nor destroyed. Light-to-heat conversion as a traditional yet constantly evolving means of converting light into thermal energy has been of enduring appeal to researchers and the public. With the continuous development of advanced nanotechnologies, a variety of photothermal nanomaterials have been endowed with excellent light harvesting and photothermal conversion capabilities for exploring fascinating and prospective applications. Herein we review the latest progresses on photothermal nanomaterials, with a focus on their underlying mechanisms as powerful light-to-heat converters. We present an extensive catalogue of nanostructured photothermal materials, including metallic/semiconductor structures, carbon materials, organic polymers, and two-dimensional materials. The proper material selection and rational structural design for improving the photothermal performance are then discussed. We also provide a representative overview of the latest techniques for probing photothermally generated heat at the nanoscale. We finally review the recent significant developments of photothermal applications and give a brief outlook on the current challenges and future directions of photothermal nanomaterials.

## 1 Introduction

The Sun, as the brightest star in the Earth’s sky, supplies almost all energy for life and human activities on the Earth. Even conventional fossil fuels are the long-term storage of solar energy.^1−3^ The Sun radiates its energy by emitting ultraviolet (UV), visible, and infrared (IR) light that carries photons with different vibrational frequencies. When encountering an object, a portion of photons in the light can be absorbed by the object, thereby heating it up. Sunlight that reaches the Earth can be largely absorbed and thus warm the atmosphere, land, and ocean, where the generated heat is essential for creating a suitable climate and environment for all living things.^4^ Moreover, energy transfer from light to heat occurs widely in physical, chemical, and biological reactions. It is one of the most fundamental processes in nature. This light-to-heat conversion process, where materials can act as light absorbers and efficiently transfer light energy into heat, is called photothermal conversion.^5^ The photothermal performance of a photoexcited material is mainly determined by two key intrinsic properties—the light-harvesting ability and the light-to-heat conversion efficiency. The investigation of photothermal materials with broadband absorption is beneficial for the utilization of renewable solar energy, while the engineering of materials with efficient heat generation abilities can be widely useful in various fields, including water evaporation,^6,7^ photothermal catalysis,^8,9^ and biomedicine.^10,11^

With the rapid development in both advanced nanotechnologies and materials science, a library of photothermal materials has been developed into nanoscale ones and designed into functional nanostructures. Similar to the classical bulk case, the photothermal effect can be universally observed in numerous nanomaterials, including metallic nanostructures,^12,13^ semiconductors,^14,15^ carbon-based nanomaterials,^16−19^ organic polymers,^20,21^ two-dimensional (2D) transition metal carbides/nitrides (MXenes),^22−24^ and their hybrids. In contrast to bulk structures, however, well-designed nanomaterials can exhibit unique thermal, optical, and electronic properties by tailoring their shapes, sizes, compositions, and surrounding environments, thus providing much more possibilities in tuning their photothermal properties. Based on the diversity in nanomaterials and their rich physiochemical properties, various strategies have been proposed and established for improving photothermal conversion capabilities. For example, metal nanostructures such as Au, Ag, Al, and Cu have attracted enormous attention because of their tunable localized surface plasmon resonances (LSPRs) ranging from the visible to IR region.^25,26^ The extremely large absorption cross-sections of plasmonic metal nanostructures and their associated plasmonic heating are highly promising for converting light into heat through the LSPR effect.^27,28^ Nanostructured semiconductors typified by metal oxides and chalcogenides represent a new type of photothermal nanomaterials, whose optical properties strongly rely on their bandgap energies.^29−31^ Either bandgap engineering or free-carrier-induced LSPRs can govern the light absorption of semiconducting nanomaterials and further improve the light-to-heat conversion efficiency.^32−34^ Carbon- and polymer-based nanomaterials are two other competitive photothermal material candidates with strong light-to-heat conversion abilities through thermal vibrations within the atomic lattices.^20,35−38^ The conjugation and hyperconjugation effects can easily facilitate the excitation of less tightly held electrons from the π orbitals to the π* orbitals, enabling broad light absorption over the solar spectrum. Apart from these widely used material options, more newly emerging classes of photothermal materials are continuously being developed, such as MXenes,^39−41^ metal–organic frameworks (MOFs),^42−46^ and covalent organic frameworks (COFs).^47−50^ Nevertheless, the mechanisms of the photothermal conversion processes can be typically attributed to the three categories mentioned above, namely, plasmonic localized heating, nonradiative relaxation of electron–hole pairs, and thermal vibrations of molecules. For enhancing the photothermal performance, photothermal nanomaterials can be designed to consist of a single component or multiple components and can involve more than one mechanism of photothermal conversion. The proper selection of materials and the ingenious design of nanostructures are consequently the most deterministic criteria for photothermal technologies.

The extensive search for new types of photothermal nanomaterials has emerged as a frontier research area for a wide range of high-potential applications in physics, chemistry, and life sciences.^20,45,51^ One important implementation of photothermal nanomaterials is the solar evaporation technology that allows steam and clean water to be produced from either seawater or wastewater, while the sustainable solar energy is collected and stored in the form of thermal, electrical, or mechanical energy. To meet the pressing demands of energy and potable water, more and more photothermal nanomaterials with diverse structural designs have been employed for seawater desalination, wastewater purification, and electricity generation.^6,7,52−54^ Heat-mediated optical manipulation is another emerging technique based on photothermal materials, where the generated optical heating reversely exerts an optothermal force on the heated nanostructures, which are thus endowed with versatile control of movements, including rotating, pulling, oscillating, walking, and swimming.^55,56^ Based on various optothermo–matter interactions, light-driven photothermal nanomaterials have been demonstrated as optothermal motors, probes, assemblers, and robots for performing complicated motions and realizing functional tasks.^57−60^ Besides mechanical movements, shape morphing expectedly occurs under excess thermal energy and thus brings new functions of photothermal materials. Localized heating at plasmonic/photonic nanostructures can easily modify their structural morphologies and thereby their LSPRs and dielectric electromagnetic (EM) resonances, leading to promising applications in color printing and display.^61−65^ The combination of photothermal nanostructures and phase-changing materials has been further developed into soft photothermal actuators with reversible and controllable deformation.^66−70^ Moreover, the photothermal effect has also been widely used to drive catalytic reactions because of a synergy of thermochemical and photochemical pathways.^8,9,45^ Thermal energy localized at active sites can effectively reduce the activation energy of photothermal catalysis and promote the transfer of charge carriers, thus greatly enhancing the catalytic process. On the other hand, photothermal nanomaterials have exhibited vast perspectives in biomedical areas because of the remote control of heating with high selectivity and spatial accuracy. Numerous applications and techniques based on the photothermal effect have been developed into photothermal therapy (PTT),^20,24,71^ drug delivery,^13,72,73^ the polymerase chain reaction (PCR),^74−77^ and even the fight against coronavirus disease 2019 (COVID-19).^78−82^

**Figure 1:**
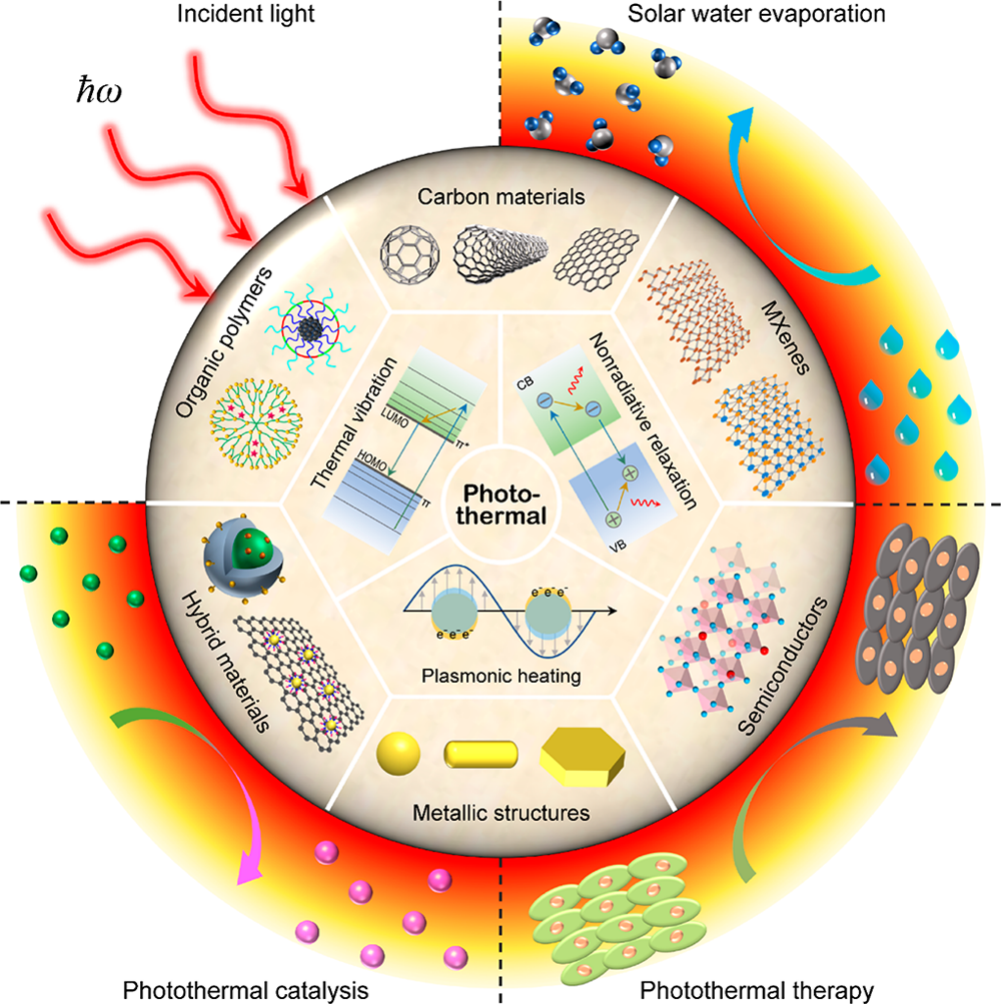
Overview of the mechanisms, categories, and applications of photothermal nanomaterials.

Several comprehensive reviews of photothermal materials have been published in recent years.^7,11,12,22^ However, most of these only review selected categories of photothermal materials or focus on one or two representatives of their promising technologies. In this review, we endeavor to provide a comprehensive overview of why photothermal nanomaterials can convert light into heat, what the material choices of photothermal converters are, and how the photothermal effect is applied (Figure 1). We will start with the fundamental principles underlying the light-to-heat conversion processes, including the LSPR effect, electron–hole generation and nonradiative relaxation, and molecular vibrations (Section 2). We will summarize the major categories of nanostructured photothermal materials and evaluate their light-to-heat conversion capabilities based on the distinct mechanisms. From this starting point, the proper material choice and rational structural design of photothermal nanomaterials for specified utilizations will become clearer and more achievable (Section 3). We will further review the latest techniques for probing photothermal heat generation at the nanoscale and categorize nanothermometers according to the physical mechanism that is employed to measure the temperature (Section 4). We will also expatiate on the recent significant developments toward the applications of photothermal nanomaterials in solar water evaporation, photothermal manipulation, photothermal catalysis, and PTT (Section 5). In the concluding section, we will provide our perspectives on the current challenges and future directions in the field of photothermal nanomaterials.

## 2 Photothermal Conversion Mechanisms

As a representative phenomenon of light–matter interaction, the photothermal effect is generally characterized with the temperature increase in a material through the absorption of light. Various materials possess different light-to-heat conversion abilities that rely on the responses of their electronic or bandgap structures to EM radiation. In this section, we will briefly introduce three major fundamental mechanisms involved in light-to-heat conversion processes.

### 2.1 Plasmonic Localized Heating

The LSPR effect is the most intriguing phenomenon appearing in metallic structures down to the subwavelength-sized dimensions. Plasmonic nanostructures can break the diffraction limit of conventional optics and further confine incident light into the nanoscale, leading to the enhancement of light–matter interaction.^83,84^ The unexpected behavior of metallic nanostructures irradiated by an external EM field is associated with their unusual electronic configurations, where electrons are coherently oscillated and redistributed at the surface. LSPRs refer to the collective oscillations of conduction-band electrons restricted inside highly conductive nanostructures. Plasmons are thus termed from the quanta of collective electron oscillations, which is the analogy to photons defined from the quanta of light waves as well as phonons treated from the quanta of sound waves. The excitation of plasmons can not only induce strong electric field enhancement near the surface of the structure but also bring about extremely large absorption and scattering cross-sections at the resonance frequency. These two important effects make metallic nanostructures perfect candidates for harvesting light and concentrating energy, endowing them with an excellent light-to-heat conversion ability.^13,85^

**Figure 2:**
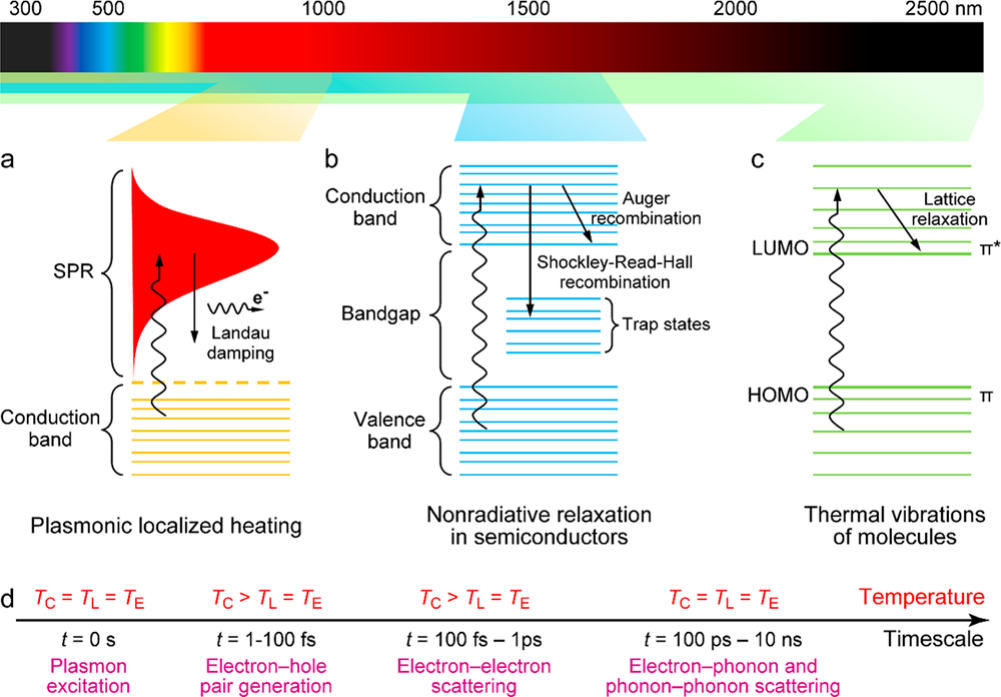
Three mechanisms of the photothermal effect with the corresponding light absorption range. (a) Plasmonic localized heating. (b) Nonradiative relaxation in semiconductors. (c) Thermal vibrations of molecules. Reprinted with permission from ref (7). Copyright 2019 Royal Society of Chemistry. (d) Physical processes of plasmon excitation and damping at different time scales. *T*_C_, *T*_L_, and *T*_E_ represent the temperatures of free charge carriers, the lattice, and the surrounding environment, respectively. Reprinted with permission from ref (28). Copyright 2022 Wiley-VCH.

The photothermal conversion process in a metallic nanostructure can be understood from the excitation and damping of the surface plasmons (Figure 2a). Metallic nanostructures as conductive materials are generally featured by a considerable number of free and polarizable electrons. Upon light illumination, a plasmonic nanostructure can absorb energy from incident photons through electron transitions. When the photon energy matches the LSPR band, a strong resonant interaction takes place, giving rise to enhanced light absorption and local field. The free conductive electrons of the metallic nanostructure are displaced from their intrinsic equilibrium state and relocated at the structure surface, together with an in-phase oscillation with the external EM wave. The resultant photoexcitation of the LSPR is a global nonequilibrium, where the dephasing and decay of the plasmons occur at an ultrafast speed (Figure 2d).^86^ To restore a thermally equilibrated state, the absorbed energy of electrons can therefore be relaxed through either the radiative re-emissions of photons or the nonradiative generation of electron–hole pairs through Landau damping.^87,88^ During such a pure quantum mechanical process of Landau damping, the energy transfer process from a plasmon quantum to a single electron–hole pair happens in a time interval from 1 to 100 fs.^86,89^ This process occurs through electron–electron collisions without loss of the absorbed photon energy. The produced energetic electrons from the nonradiative plasmon decay are termed as hot charge carriers, whose distribution is highly nonthermal within the first 100 fs.^90^ The hot electrons further quickly interact with low-energy electrons on the time scale ranging from 100 fs to 1 ps. This electron–electron collision is an inelastic Coulombic process that converts electron energy into heat. At the same time, low-energy electrons couple with the metallic lattice through electron–phonon scattering processes with a period of several to hundreds of picoseconds. This relaxation step leads to the lattice thermalization of the nanostructure as well as a Fermi–Dirac-like distribution of electrons.^91^ In the final step, the thermal energy inside the metallic structure is released to the surrounding environment through phonon–phonon collisions in the time scale of 100 ps to 10 ns. With thermal dissipation and lattice cooling, the electrons of the metallic nanostructure in the conduction band eventually return to their ground states before the photoexcitation.

In summary, the plasmonic effect for localized heating in metallic nanostructures involves the excitation of LSPRs with enhanced absorption, generation, and relaxation of hot electrons, and heat transfer to the surrounding medium. The energy transfer from the absorbed photons to the conduction-band electrons of metallic nanostructures goes through: (1) a resonant excitation of surface plasmons; (2) an athermal process of electron–electron collisions; (3) a fast lattice thermalization through electron–phonon scattering; and (4) a slow thermal dissipation through phonon–phonon collisions.

### 2.2 Nonradiative Relaxation in Semiconductors

Besides the excitation of the LSPRs in metallic nanostructures, the direct interband/intraband electron transitions of nonplasmonic semiconductors can also display the photothermal effect. When a semiconducting material is excited by photons with sufficient energies, electron–hole pairs are generated with their energies comparable to the bandgap. The energy of the excited electrons can be either released by emitting photons or transferred to the material lattice through nonradiative relaxation.^7,92^ The release of phonons instead of photons in a semiconductor through the recombination of charge carriers can increase heat loss and therefore lead to a local temperature increase of the lattice. The established thermal distribution is strongly dependent on the characteristics of the light absorption and surface/bulk recombination of the semiconductor. Two types of the nonradiative relaxation processes, including Shockley–Read–Hall and Auger recombination,^93,94^ are ultimately responsible for heat generation in semiconductors (Figure 2b). Auger recombination, which occurs with three carriers, is an intrinsic process relying on the properties of the involved material. The effect of Auger recombination becomes ascendant with decreasing bandgaps. When an electron–hole pair recombines without the emission of photons, their energy can be transferred to either another electron higher in the conduction band or another hole deeper in the valence band. The third energetic carrier normally thermalizes back to the band edge through lattice vibrations. Shockley–Read–Hall recombination is an alternative nonradiative relaxation that depends on the quality of the material. In the presence of defects/impurities in a semiconductor, midgap energy states are usually created within the bandgap due to the defect modification of the electronic structure.^95^ These defect states are also known as trap levels that capture charge carriers. The Shockley–Read–Hall recombination process is thus called trap-assisted recombination. It involves a two-step process. Conduction-band electrons first relax to the trap level and then move to the valence band, where a hole is annihilated. These relaxation processes are accompanied with the exchange of thermal energy with the material. For the case of the defect traps located at or near the surface of the semiconductor, the trap-assisted recombination process is termed as surface recombination, which is dependent on the density of surface defects.

### 2.3 Thermal Vibrations of Molecules

Carbon-based materials and some organic polymers also demonstrate excellent light absorption characteristics and are very competent in the heat generation through lattice vibrations. Although the usual carbon bonds like C-C, C-O, and C-H possess large energy differences between the σ and σ* orbitals that can be hardly excited, loosely held electrons in these materials can be easily excited from the π orbitals to the π* orbitals under low-energy irradiation.^23,96,97^ When the energy of incident photons satisfies an electronic transition within the material, the π electrons are excited from the ground state (highest-occupied molecular orbital, HOMO) to a higher energy state (lowest-unoccupied molecular orbital, LUMO) (Figure 2c). After the relaxation of the excited electrons to the ground state through vibration–electron coupling, the excess energy is released in the form of heat. Moreover, the conjugation or hyperconjugation of the π orbitals can deeply modify the electron transitions between HOMO and LUMO, whose gap energy decreases with the increase in the number of π bonds.^98,99^

### 2.4 Basic Mathematical Descriptions for Photothermal Conversion

To elucidate the light-to-heat conversion process, it is essential to consider the light-harvesting ability and heat generation of a material as well as heat transfer and loss. Owing to the fundamental difference in the physical mechanisms, diversely categorized photothermal materials have various mathematical descriptions for their optical and thermal properties. In this section, basic universal equations are presented for understanding photothermal conversion in terms of three aspects including light harvesting, light-to-heat conversion, and heat transfer.

#### 2.4.1 Light Harvesting

One of the critical factors to evaluate the photothermal performance is the light-harvesting ability that reveals how well a photothermal material absorbs the energy of incident photons. The light absorption is an important process of light-to-heat conversion. Absorptance is defined as the fraction of the energy of incident photons that is absorbed by a material. The absorbed energy for a certain material depends on the range of absorption over the spectrum of the incident light and the intensity of absorbance for each wavelength. By integrating the absorption intensity of the photothermal material over the spectral range, the absorptance can be calculated by the energy ratio of the total absorbed light to the incident radiation. At an incidence angle of θ, the overall absorptance *A*(θ) of a light absorber can be expressed as^51,100^
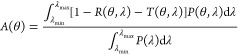
2.1where *P*(λ), λ_max_ and λ_min_ represent the wavelength-dependent radiation power and the maximum and minimum wavelengths of the incident light and *R*(*θ,λ*) and *T*(*θ,λ*) stand for the total reflectance and transmittance of the absorber at the wavelength λ, respectively. According to this equation, the absorptance can be enhanced by reducing the transmittance and reflectance of the photothermal material. Moreover, broadband light absorption is crucial for light absorbers to harvest enough light for obtaining high light-to-heat conversion efficiencies.

For the case of photothermal materials dispersed in homogeneous semitransparent media, light absorption is a cumulative process with an exponential decay that can be described by Beer–Lambert’s law as *I* = *I*_0_*e*^–*κcl*^, where *I* and *I*_0_ represent the light radiation intensity after and before the absorption, κ stands for the extinction coefficient, *c* is the particle concentration, and *l* is the length of the optical path. To further determine how much of the light is absorbed by the material, absorbance *a* is defined as the attenuation of the incident radiation power. It can be calculated from the transmittance *T* as
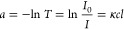
2.2The absorbance is therefore proportional to the extinction coefficient of the absorber and the amount of the absorber per unit area. The extinction coefficient is the intrinsic property of the absorber. It depends on the material, size, and shape.

#### 2.4.2 Light-to-Heat Conversion

Besides the light absorption of a photothermal material, the light-to-heat conversion efficiency is another essential factor that directly quantifies the absorbed energy transferred to thermal energy, instead of radiative re-emission of photons. One straightforward method for determining the conversion efficiency is to measure the increase in temperature and calculate the heat generation induced by an incident light. The photothermal conversion efficiency η can be written as^101,102^
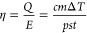
2.3where *Q* is the generated thermal energy by the absorber, *E* represents the total energy of the incoming light, *c* and *m* denote the specific heat and mass of the photothermal material, Δ*T* is the temperature increase of the material under the light irradiation, *p* is the power density of the light source, and *s* and *t* represent the radiation area and time. By use of this method, all of the incident light including the reflected, scattered, absorbed, and transmitted photons originating from the incident light is taken into account as the input energy. The advantage of this strategy is that only the absorbed photons contribute to the generation of thermal energy in the photothermal material. As the heat generation is dependent on the amount of the used photothermal material, it is difficult for this method to directly compare the efficiency values among different materials. Moreover, the heat transfer from the photothermal material to the surrounding medium is not considered in the calculation.

The heat loss to the environment can be estimated by recording the temperature decay process after the incident light is removed. The equation of the photothermal conversion efficiency η can then be expressed as^103−105^
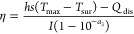
2.4where *h* is the heat transfer coefficient, *s* represents the surface area of heat transfer, *T*_max_ is the equilibrium temperature, *T*_sur_ represents the temperature of the surrounding environment, *Q*_dis_ stands for the heat dissipated by the surrounding environment, *I* is the radiation intensity of the incident light, and *a*_*λ*_ represents the absorbance of the photothermal material at the wavelength λ. Although only the absorbed photons are treated as the input energy, this equation quantitatively eliminates the impact of the heat transfer and the concentration of the photothermal material on the light-to-heat conversion efficiency. The photothermal conversion abilities of various materials can therefore be readily compared by this method. But there are a number of parameters that need to be experimentally measured. Some of them are not trivial and can significantly affect the ultimate value of the photothermal conversion efficiency. Some recent works have modified eq 2.4 and provided a more straightforward and reproducible approach for the evaluation of the photothermal conversion efficiency.^106,107^

#### 2.4.3 Heat Transfer

After the incident light is absorbed by a photothermal material, the photon energy is converted into thermal energy through a light-to-heat conversion process. The generated heat will be further transferred to other lower-temperature materials or released to the surrounding environment. Therefore, heat transfer is the third important process in a photothermal conversion system. The transfer of thermal energy from one material to another is driven by the thermal gradients between the materials, which can be realized in three main means, which are conduction, convection, and radiation.^51,104^

Thermal conduction usually occurs within a material or contiguous objects, where heat spontaneously flows from the higher-temperature part (the light absorber) to the lower-temperature one (heat transfer object). The conduction energy can be expressed as
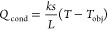
2.5where *k* represents the thermal conductivity of the photothermal material, *s* represents the surface area of heat transfer, *L* is the conduction length of the light absorber, and *T*_obj_ and *T* are the steady temperatures of the heat transfer object and the light absorber.

Thermal convection refers to the heat energy transfer induced by the movement of fluid. The convection energy from the heating source to the fluid can be written as
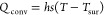
2.6where *h* is the heat transfer coefficient. As the reciprocal of thermal insulance, the heat transfer coefficient depends on the physical properties of the fluid and the thermohydraulic conditions.

Thermal radiation takes place in all objects through EM waves without the requirement of any medium. According to the Stefan–Boltzmann law, the emission energy is proportional to the fourth power of the temperature. When an object emits thermal radiation based on its temperature, it also absorbs radiation from the surrounding objects. The thermal radiation energy is the net energy exchange between the hot object and the cold environment, which can be expressed as
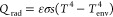
2.7where *ε* is the emissivity, σ represents the Stefan–Boltzmann constant, and *T*_env_ stands for the temperature of the environment.

Consequently, the heat transfer not only is dependent on the physical properties of the photothermal material but also is affected by the circumstance of its surrounding environment. The three mathematical expressions provided above offer efficient ways to regulate the heat transfer process. For interfacial solar water evaporation, thermal conduction is the dominant process that requires the minimization of heat loss to the surrounding environment. The generated heat can thus be localized on the surface of the photothermal material for efficient vapor generation. For volumetric water heating, the convection process needs to be first considered, where a promoted heat transfer is demanded for a fast increase in the temperature of the surrounding environment. In short, the proper choice of both photothermal materials and their surrounding media is essential for photothermal applications with desired purposes.

## 3 Recent Developments of Photothermal Nanomaterials

With the development of advanced nanofabrication methods in the last few decades, nanomaterials, for example, metallic/semiconductor nanostructures, carbon-based nanomaterials, organic polymer nanomaterials, and 2D nanomaterials, have been thoroughly investigated with various beneficial and functional qualities. The light-to-heat conversion mechanisms and intriguing properties of differently categorized nanomaterials are summarized in Table 1. Photothermal conversion as an ancient technology has recently received extensive attention and regained a breakthrough. The capability of photothermal nanomaterials to enhance light absorption, convert heat, and conduct thermal energy is highly dependent on the material choice and structural design. In this section, we will summarize the recent progresses in the development of both new photothermal materials and advanced methods for structural engineering with excellent light-to-heat conversion performances.

**Table 1:** Proposed Photothermal Conversion Mechanisms and Advantages of Different Types of Nanomaterials for Photothermal Applications

references	photothermal materials	working mechanisms	advantages for photothermal applications
(13, 85)	plasmonic metals	LSPR effect	facile synthesis, tunable plasmon resonance, and large absorption cross-sections
(32, 33)	slightly doped and intrinsic semiconductors	nonradiative recombination of electron–hole pairs	facile synthesis, low toxicity, and strong extinction coefficients in the NIR region
(34)	heavily doped semiconductors	LSPR effect	
(326)	carbon-based nanomaterials	nonradiative relaxation of delocalized π electrons	high chemical stability, broadband light absorption, and lightweight
(98)	organic polymers	nonradiative relaxation of delocalized π electrons	versatile molecular designs, strong absorption of NIR light, and good biocompatibility
(22, 364)	2D nanomaterials	nonradiative recombination of electron–hole pairs and LSPR effect	layered structures, broad light absorption band, and high photothermal conversion efficiencies

### 3.1 Metallic Nanostructures

Noble metal nanostructures show attractive photothermal conversion properties because of the excitation of their strongly confined LSPRs. According to the Drude–Lorentz model, the high density of free electrons and their collective oscillations can give rise to the LSPR effect at the metal–dielectric interface.^108,109^ Plasmon resonances are excited when the incident photon energy matches the LSPR band of metallic structures. The redistribution of the excited electrons contributes to the generation of plasmonic heating, which can be finely controlled by external irradiation. The LSPR frequency can be further tailored by changing the materials, sizes, and shapes of metallic nanoparticles, as well as their surrounding environments and assembly configurations.^110,111^

#### 3.1.1 Effect of the Material and Geometry

**Figure 3:**
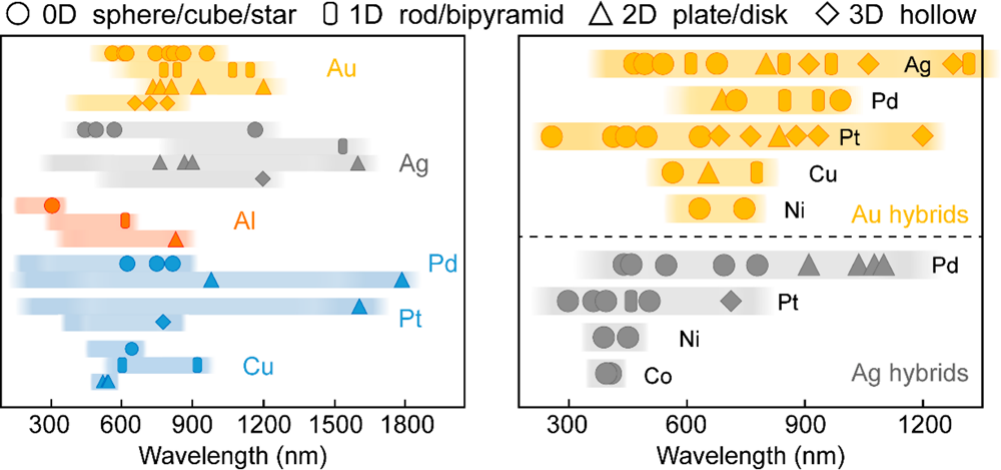
Typical plasmonic spectral bands of material- and shape-dependent metallic nanostructures. Left: homogeneous metals (Au,^101,164−182^ Ag,^183−192^ Al,^131,145,193−195^ Pd,^196−199^ Pt,^186,200^ and Cu.^136,201−204^ Right: metal hybrids (Au hybrids: Au-Ag,^156,201−218^ Au-Pd,^219−224^ Au-Pt,^225−234^ Au-Cu,^235−238^ and Au-Ni;^239,240^ Ag hybrids: Ag-Pd,^241−248^ Ag-Pt,^249−251^ Ag-Ni,^252,253^ and Ag-Co^254,255^) according to their different geometries.

Because of the LSPR effect, numerous types of metallic nanostructures for light-to-heat conversion have been explored, such as Au,^112−119^ Ag,^120−124^ Pd,^125−130^ Al,^131−135^ Cu,^136,137^ Ge,^138,139^ and other metals. Au and Ag are two of the most commonly employed metals in photothermal conversion because of their high free charge carrier concentrations and relatively low Ohmic losses.^140,141^ Their plasmon resonances can be finely tuned from the visible to near-infrared (NIR) region through control of the size and morphology (Figure 3, left panel). However, another loss mechanism originating from interband transitions plays an important role for Au and Ag at optical frequencies. For Au structures, the interband losses are dominant at the short wavelengths of the visible region, while the intraband losses are high in the NIR range and limited in the shorter-wavelength region.^142^ In addition, the plasmonic properties of Ag structures are easily degraded due to rapid oxidation and sulfuration under ambient conditions. The earth-abundant metals such as Al and Cu are newly emerging as intriguing alternatives to Au and Ag in plasmonics. Cu and Al are more cost-effective and possess similar LSPR performances to those of Au and Ag.^131,143^ Moreover, Al nanostructures can support strong plasmon resonances in the UV range with extremely high photothermal conversion efficiencies.^144^ Although Al suffers from chemical instability, the formation of a passivation layer of aluminum oxide can effectively protect the structure without degrading its LSPR response. The limitation for Al structures is the broad line widths of their plasmon resonances in the visible range because of interband transitions.^145^ In addition, a wide variety of metal materials including Ni, Co, Cr, Pt, and Pd have been demonstrated to exhibit LSPRs in the UV region.^144^ Among them, Pt and Pd are of practical interest in photothermal catalysis owing to their outstanding catalytic properties and high thermal stability.^146,147^ In general, plasmonic nanoparticles made of these metals can be synthesized by bottom-up methods, where crystal structures are grown by stacking metal atoms. The bottom-up methods are usually based on the chemical reduction of metal salts and the nucleation of tiny particles. One of the famous synthetic approaches is seed-mediated growth with a two-step process.^148^ The first step is the preparation of small seeds through homogeneous nucleation. The second step is overgrowth on the seeds using shape-directing agents, where nanoparticles are overgrown into the desired geometry and size. A large number of asymmetric metal nanostructures can be facilely synthesized by changing the seeds, metal salt, reductant, and capping agent, as well as the shape-directing agent.^149^ Asymmetric structures usually have more sharp tips, corners, and edges where plasmonic heating is generated and concentrated because of largely enhanced EM fields. The geometry-dependent photothermal conversion efficiency has been well demonstrated in both numerical simulations and experiments. The effect of the morphology on the heat generation in plasmonic nanoparticles has been numerically investigated using Green’s dyadic method.^150^ The heat generation can be well quantified by mapping the heating power density within the studied Au nanostructures. This geometry effect has also been experimentally shown in various differently shaped structures, including nanocubes, nanorods, nanoplates, nanocages, and branched nanostructures (Figure 3, left panel).^151,152^ Nanospheres and nanocubes with high symmetry usually exhibit strong absorption resonance.^153,154^ On the contrary, low-symmetry polyhedron nanoparticles show broader but weaker absorption resonances with multiple plasmon peaks. The most straightforward way to broaden the absorption spectrum is to mix plasmonic nanoparticles with different sizes and shapes.^85^ But this method is practically unattractive, as it requires the parallel synthesis of various types of nanoparticles and the careful control of the amount ratio of these different nanoparticles. An alternative strategy is to grow new particles on pre-existing structures to broaden the plasmon resonances. A nearly ideal type of blackbody nanostructure composed of a Au nanorod with an attached Au nanosphere has been reported.^155^ This material has a nearly perfect absorption of 98–99% over a broad spectral range from 400 to 1400 nm.

As the LSPR bands of homogeneous metallic structures are intrinsically limited by the materials, metal hybrids, including alloys, can be obtained by reacting two or more metal elements (Figure 3, right panel). Metal alloys containing less conductive metals can effectively lower the free charge concentration and thus reduce the optical losses.^108^ Their LSPR properties can also be tailored by adjusting the proportion of each plasmonic or functional reactant. For instance, Au-Ag alloyed nanostructures can support plasmon resonances over a much broad range of the UV–visible spectrum and exhibit distinct blueshifts by decreasing the atomic ratio of Au to Ag.^156^ Because the change in the proportion of an alloy alters the effective dielectric constant of the alloy, the photothermal conversion efficiency can therefore be greatly improved with enhanced light absorption for Au-Ag alloy nanostructures.^157−160^ Apart from the tunable LSPR frequencies, the formation of alloys offers a new route for improving the sensitivity of plasmonic structures through dispersion engineering.^161^ The real permittivities of alloyed structures such as Au-Pd^162,163^ and Ag-Ti^161^ can be reduced compared to the pure metals, leading to the increase in sensitivity.

#### 3.1.2 Effect of Plasmon Coupling

**Figure 4:**
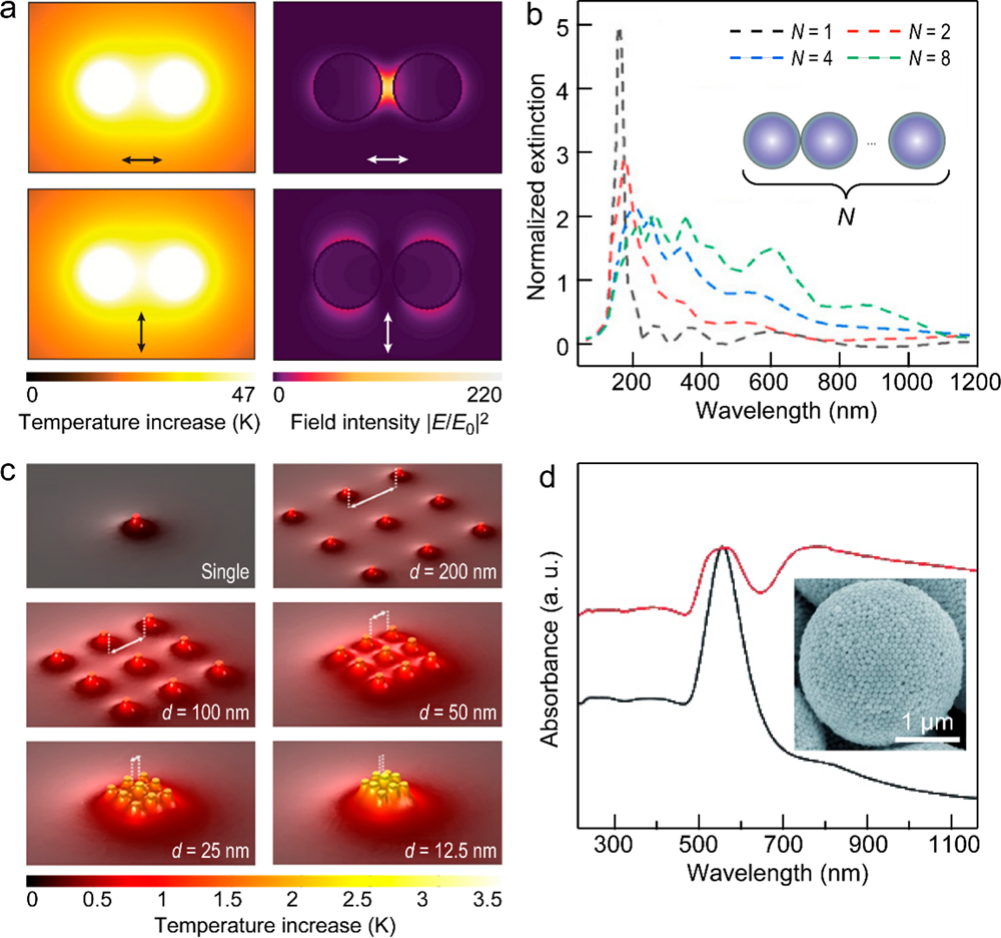
Plasmon-coupling-induced absorption broadening and related heat effects. (a) Temperature distributions (left column) of a Au dimer under the longitudinal (top) and transverse (bottom) polarization excitation and corresponding electric near-field enhancements (right column). Reprinted with permission from ref (261). Copyright 2017 American Chemical Society. (b) Dependence of the calculated normalized extinction spectra of one-dimensional (1D) Al nanoparticle chains on the particle number *N*. Reprinted with permission from ref (133). Copyright 2016 Springer Nature. (c) Calculated temperature maps of a single array and five arrays of nine Ag nanoparticles in a square lattice with lateral periods from 12.5 to 200 nm. Reprinted with permission from ref (266). Copyright 2020 American Chemical Society. (d) Measured absorption spectra of self-assembled Au colloidosomes (red) and the Au nanoparticles (black) suspended in 1-butanol. The inset shows the corresponding scanning electron microscopy (SEM) image of the Au colloidosomes. Reprinted with permission from ref (271). Copyright 2015 Wiley-VCH.

The key challenge for improving the photothermal conversion efficiencies of plasmonic nanomaterials is to realize broadband absorption over the solar spectrum. But metal nanostructures often absorb photons at one or a few certain bands, limiting their photothermal performances.^144^ The most widely used strategy is to assemble two or more metallic nanoparticles into plasmon-coupled structures where the interparticle nanogaps are formed and known as “hotspots”.^256^ The generation of hotspots can not only achieve strong EM field enhancement but also produce multiple plasmon modes through plasmon coupling. The enhanced local field can give rise to pronounced photothermal conversion in the interparticle nanogaps. The overall photothermal performance of the assembled nanoparticles is based on their collective heating ability and plasmon coupling effect.^257^ The plasmonic properties of the coupled nanostructures are sensitively dependent on the particle number, gap distance, structural configuration, and polarization state of the incident light.^258^ When two metallic nanoparticles are placed close to each other, a plasmonic dimer as the simplest coupled structure is formed. According to plasmon hybridization, the interaction between the two nanoparticles produces a lower-energy symmetric (bonding) plasmon resonance mode and a higher-energy antisymmetric (antibonding) resonance mode.^259^ The total heat generation and temperature distribution in a plasmonic dimer have been numerically calculated.^260^ The calculated temperature is distributed nonuniformly around the nanoparticles even at the nanoscale. The plasmonic heating has been found to be dependent on the polarization state of the incident light. When the external electric field is polarized along the dimer axis, the heat generation increases as well as the electric field enhancement. Interestingly, an extraordinary photothermal isosbesticity has been discovered in plasmonic nanostructures, where the temperature is invariant with the change of the illumination polarization state at specific wavelengths (Figure 4a).^261^ The isosbestic wavelength of a given plasmonic nanostructure can be found at the intercrossing of the absorption spectra under two different polarization directions. Since the ability of a single dimer to broaden the LSPR band is limited, more nanoparticles need to be assembled and interact together to increase the light absorption bandwidth.^52,262−264^ The linear assembly of Al nanoparticles has been shown to broaden the extinction peaks with distinct redshifts toward the visible and NIR regions as the particle number is increased (Figure 4b).^133^ Randomly distributed Au nanoparticles have been employed as a perfect absorber to fully absorb light. The average absorbance can reach up to 99% in the spectral range from 400 nm to 10 μm because of the suppression of back reflection.^265^ Analogous to the distance-dependent electric field from a charged particle, the temperature of a heated structure drops slowly as ∼1/*d* from the particle surface.^260^Figure 4c shows the simulated temperature profiles for the arrays of nine Ag nanoparticles with varied periodic spacings.^266^ When a large number of nanoparticles are irradiated at the same time, the overall temperature of the entire particle system can reach large but homogeneous values by adding together all the temperature contributions.^267^ Three-dimensional (3D) or more disordered nanostructures, such as plasmonic colloidosomes that are generally prepared by assembly approaches, have also been used to produce strong plasmon coupling.^123,268−270^ For example, Au nanospheres have been assembled into 3D black plasmonic colloidosomes by use of an emulsion-templating method (Figure 4d).^271^ The fabricated colloidosomes have hexagonal close-packed multilayer shells and exhibit intense broadband light absorption. The assembly process is universal, and this method is suitable for nanoparticles with different sizes and shapes. In general, photothermal heat generation is highly sensitive to the assembly state of metal nanoparticles (particle number, spacing, and configuration) and the polarization state of the incident light. To take advantage of the plasmon coupling effect, the particle arrangement and structural design need to be carefully considered.

### 3.2 Semiconductors

**Figure 5:**
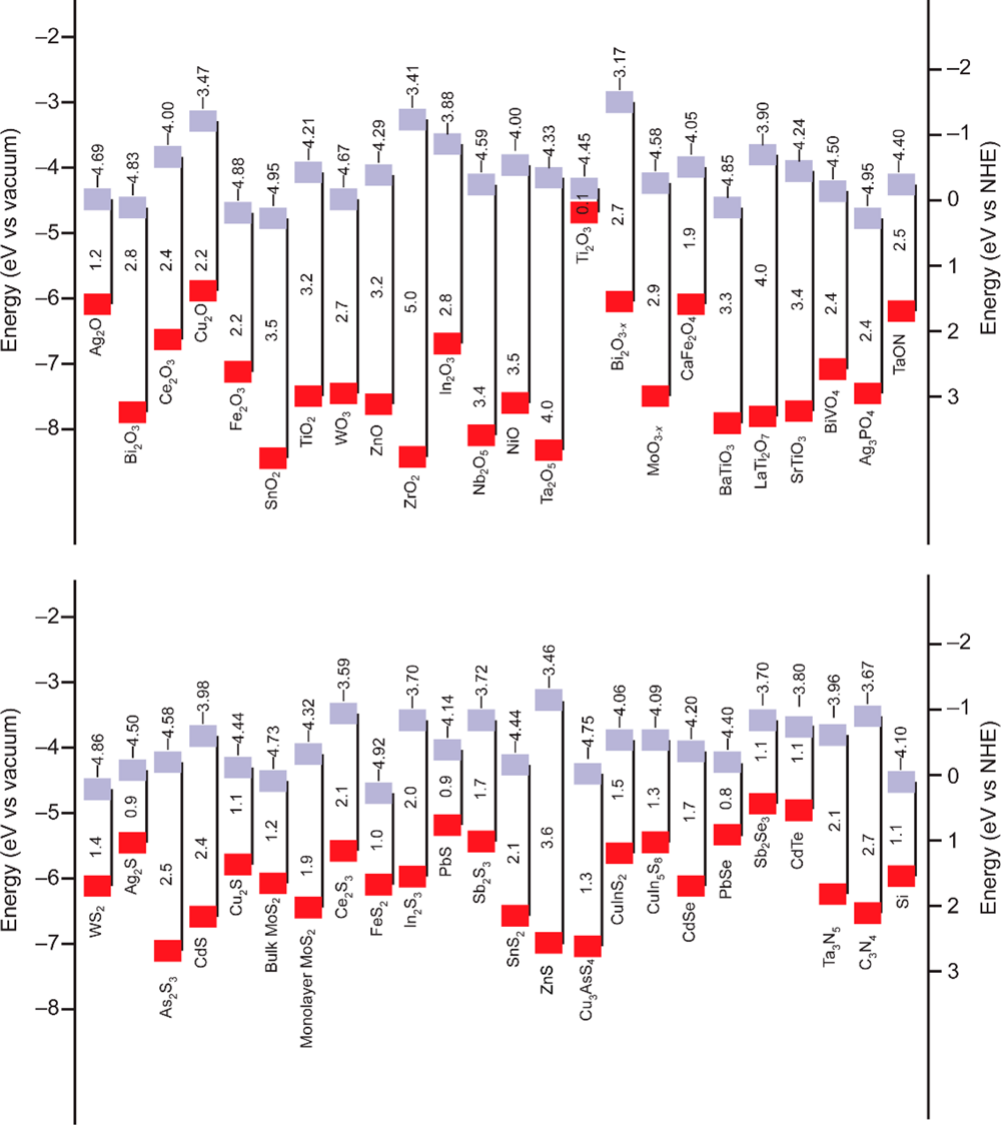
Band edge positions and bandgaps with respect to the vacuum level and the normal hydrogen electrode (NHE) for selected semiconductors including oxides, chalcogenides, nitrides, and silicon. The top squares indicate the conduction band edges. The bottom squares indicate the valence band edges. The top numbers represent the exact conduction band levels, and the numbers between the squares show the bandgaps. The data are taken from ref (272) with permission from the Royal Society of Chemistry and modified according to the new works.^273,274^

**Figure 6:**
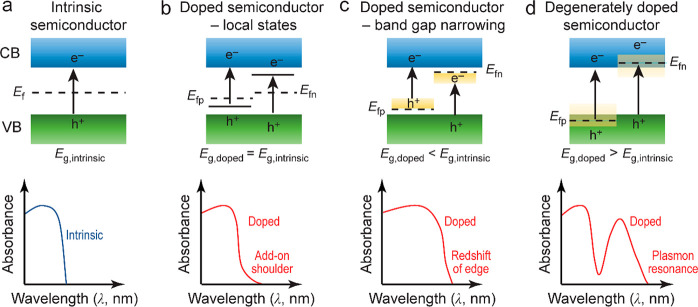
Schematics for engineering the band structures of semiconductors. (a) An intrinsic semiconductor. (b) Doping-induced formation of shallow-level energy states in the bandgap. (c) Doping-induced formation of deep-level energy states in the bandgap. (d) Degenerate-doping-induced LSPR. The electronic band structures are shown on the top, and the corresponding optical absorption curves are displayed on the bottom. Reprinted with permission from ref (7). Copyright 2019 Royal Society of Chemistry.

Semiconductor materials, such as metal oxides and chalcogenides, are another type of extraordinary candidate for photothermal conversion because of their low cost, facile synthesis, and low toxicity. Semiconductors are not easily vulnerable to photodegradation or photobleaching compared to organic photothermal materials.^14^ They also possess tunable absorption wavelengths and exhibit strong extinction coefficients in the NIR region. The bandgaps of semiconductors govern the light absorption based on the free charge carrier generation inside the material. Figure 5 summarizes the bandgaps and band edge positions of some commonly studied semiconductors, including oxides, chalcogenides, nitrides, and silicon.^272−274^ When the photon energy of incident light matches the bandgap energy, the incoming photons absorbed by a semiconductor can cause the generation of electron–hole pairs (Figure 6a). The heat generation relies on the nonradiative recombination of the electron–hole pairs. Semiconductors usually possess much lower free charge carrier concentrations than metal materials. Semiconductor materials are therefore generally translucent in the IR region but opaque in the visible region.^51^ To extend the absorption range, doping is one of the classical means to increase the free charge carrier concentrations of semiconductors. While the light absorption of a semiconductor is enhanced by doping, the photothermal conversion efficiency can also be improved owing to the increased probability of nonradiative recombination.

Through the stratagem of defect or impurity introduction, not only can the band energy be shifted, but also new energy states can be created within the bandgap. There are several doping-induced changes during the bandgap engineering of semiconductors, including (1) the emergence of intraband energy states; (2) the narrowing of the bandgap; and (3) the formation of impurity bands in degenerately doped semiconductors.^7^ In the first case, doping introduces defect and trap-level states without changing the bandgap (Figure 6b). The defect-level states can work as charge recombination centers for the relaxation of conduction-band electrons, resulting in the extension of the absorption spectrum to longer wavelengths. An add-on shoulder can often be observed in the absorption curve of a defect-level-doped semiconductor. N-doped TiO_2_ was first prepared in 2001. It possesses a visible-light absorption capability.^275^ From then on, doping has been extensively employed for TiO_2_ with either non-metal anions of C, N, S, and P at the O sites or transition metal cations of Fe, Co, Cu, Cr, and Ni at the Ti sites.^272,276−279^

In the second case of doping, the position of the valence or conduction band is shifted instead of the introduction of any new energy levels (Figure 6c). The bandgap thus becomes narrower compared to that of the pristine semiconductor, resulting in both the broadening and redshift of the absorption edge. Upon light illumination, photons that possess higher energies than the band energy of the narrow bandgap semiconductor can generate above-bandgap electron–hole pairs.^33^ The subsequent relaxation of the electron–hole pairs to the band edges can effectively convert the extra energy into heat through a thermalization process. On the contrary, most photons absorbed by the broad bandgap semiconductor are radiatively released through light emissions, leading to a lower photothermal conversion efficiency. Various narrow bandgap semiconductors, including black TiO_2_,^32,280,281^ Ti_2_O_3_,^33^ MoO_3_,^282^ and Fe_3_O_4_,^283,284^ have been developed as light-to-heat converters. Black TiO_2_ with broadband light absorption has recently received intensive attention as a perfect light absorber.^285−288^ It can reduce the recombination of electron–hole pairs and further enhance visible light absorption. Since the first discovery in 2011,^32^ various fabrication strategies have been proposed for the development of black TiO_2_ with superior photothermal properties. The introduction of surface disorder and the creation of oxygen vacancies are the two main approaches for bandgap engineering. The introduction of surface disorder can destroy the lattice periodicity and modify the edges of the conduction and valence bands, resulting in the narrowing of the bandgap. The created oxygen vacancies can serve as traps for reducing the recombination of photogenerated charge carriers and thus improve the photothermal performance. Remarkably, the bandgap of black TiO_2_ was reported in 2017 to be further narrowed by adding Ti^3+^ ions.^33^ Ti_2_O_3_ nanoparticles were synthesized with an excellent absorption capability and a high photothermal conversion efficiency of 92%. Because of the localized hybridization between titanium and oxygen, Ti_2_O_3_ tends to generate oxygen vacancies instead of titanium vacancies.^289^ The presence of vacancies can promote the band overlap near the Fermi level and therefore improve the electronic transport of Ti_2_O_3_.^290^

Degenerately doped semiconductors have recently been discovered with strong LSPRs in the NIR region (Figure 6d). Both n-type and p-type semiconductors can be heavily doped to generate high-enough free charge carrier concentrations, which is necessary for achieving similar absorption characteristics to those of noble metals. The phenomena of free-carrier-induced LSPRs have been widely observed in many metal chalcogenides and oxides, including Cu_2-*x*_S, Cu_2-*x*_Se, Cu_2-*x*_Te, Fe_1-*x*_S_2_, MoO_3-*x*_, WO_3-*x*_, ZnO, and CdO.^34,291−295^ In addition to the structural parameters (material, size, shape) that can affect the plasmonic responses, the plasmon resonances of heavily doped semiconductors can also be tuned by controlling their free charge carrier concentrations. The LSPR frequency can be largely spanned from the visible to far-infrared (FIR) region. Increases in the free charge carrier concentration can cause blueshifts of the LSPR peak and increases in the resonance amplitude. The free charge carrier concentrations of the doped semiconductors can be controlled from 10^18^ cm^–3^ to 10^21^ cm^–3^, which are still smaller than those of the conventional plasmonic metals^34^ on the order of 10^22^ cm^–3^. However, the doping level for many semiconductors is limited owing to the perturbation of high-concentration doping on the band structure. Many factors, including the fraction of the active dopant, the solid solubility limit, and doping compensation effects, need to be considered.^296−298^

### 3.3 Carbon-Based Nanomaterials

**Figure 7:**
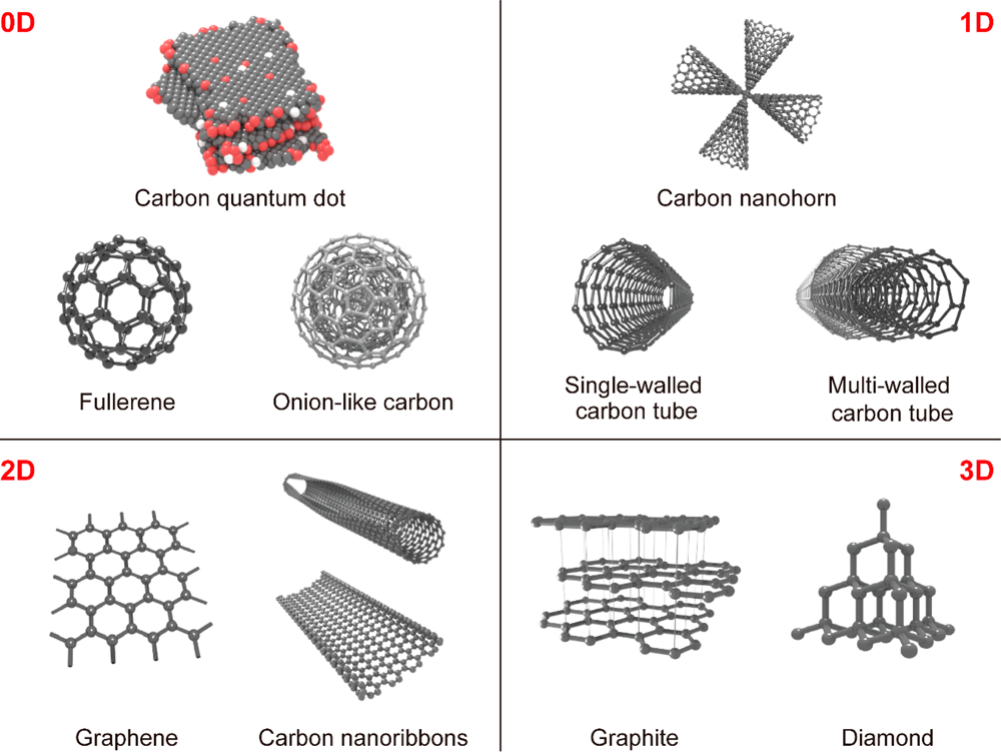
Carbon-based nanomaterials with different dimensionalities. Reprinted with permission from ref (35). Copyright 2015 American Chemical Society.

Carbon is one of the most abundant elements in the universe and can be found in all life forms. The atoms of carbon can bond with each other in diverse hybridization states (sp, sp^2^, sp^3^), resulting in a variety of carbon allotropes (Figure 7).^35^ Well-known carbon materials include carbon dots,^299−303^ nanodiamond,^304,305^ fullerene,^306,307^ graphite,^308−312^ carbon nanotubes (CNTs),^313−318^ graphene and its derivatives.^36,319−325^ All these carbon allotropes are excellent candidates as photothermal materials due to their high chemical stability, broadband light absorption, lightweight and low cost.^326^ Their light-to-heat conversion relies on the excitation of loosely held π electrons and relaxation to their ground states. The light absorption of carbon-based nanomaterials can be extended to a wide range of the solar spectrum. Many efforts have therefore been made to increase the absorption intensity and reduce the surface light reflection rather than broadening the absorption range. The optical properties of carbon-based nanomaterials vary with specific structural parameters, including the size, shape, doping, and number of layers.^327^ These physicochemical factors are also related to the fabrication methods. The fabrication methods include both top-down and bottom-up approaches. In the top-down approaches, bulk materials are reduced in size, such as mechanical exfoliation. In the bottom-up approaches, the carbon-based nanomaterials are constructed from the atomic level, such as chemical vapor deposition and epitaxial growth.^328,329^ When carbon-based materials are fabricated into porous nanostructures, the light reflection is significantly reduced through the minimization of the influence of the incidence angle and the reduction of the effective refractive index of the materials.^7^ For instance, the synthesis of a double-layer structure consisting of an exfoliated graphite layer and a carbon foam layer has been demonstrated.^312^ The reflectivity of this porous nanostructure reaches down to 3% within the solar spectrum ranging from 250 to 2250 nm. Porous graphene sheets^330,331^ and CNTs^313,314^ have also been fabricated to improve light absorption. Optical microcavities can be formed between two sides of a spacer layer. These porous structures can therefore effectively confine light within the structures and significantly enhance the interaction between the materials and light.^332^ A porous network comprising graphene sheets has been reported to achieve 97% absorption across 200–2500 nm because of the multiscattering effect.^331^ 98% absorption of visible irradiation and almost 100% absorption of NIR light have been realized with vertically aligned graphene sheets.^330^ In addition, hollow CNTs can be fabricated into a hierarchically nanoporous network structure,^314^ which exhibits broad absorption of 99% and high photothermal conversion of 86.8%.

### 3.4 Organic Polymer Nanomaterials

**Figure 8:**
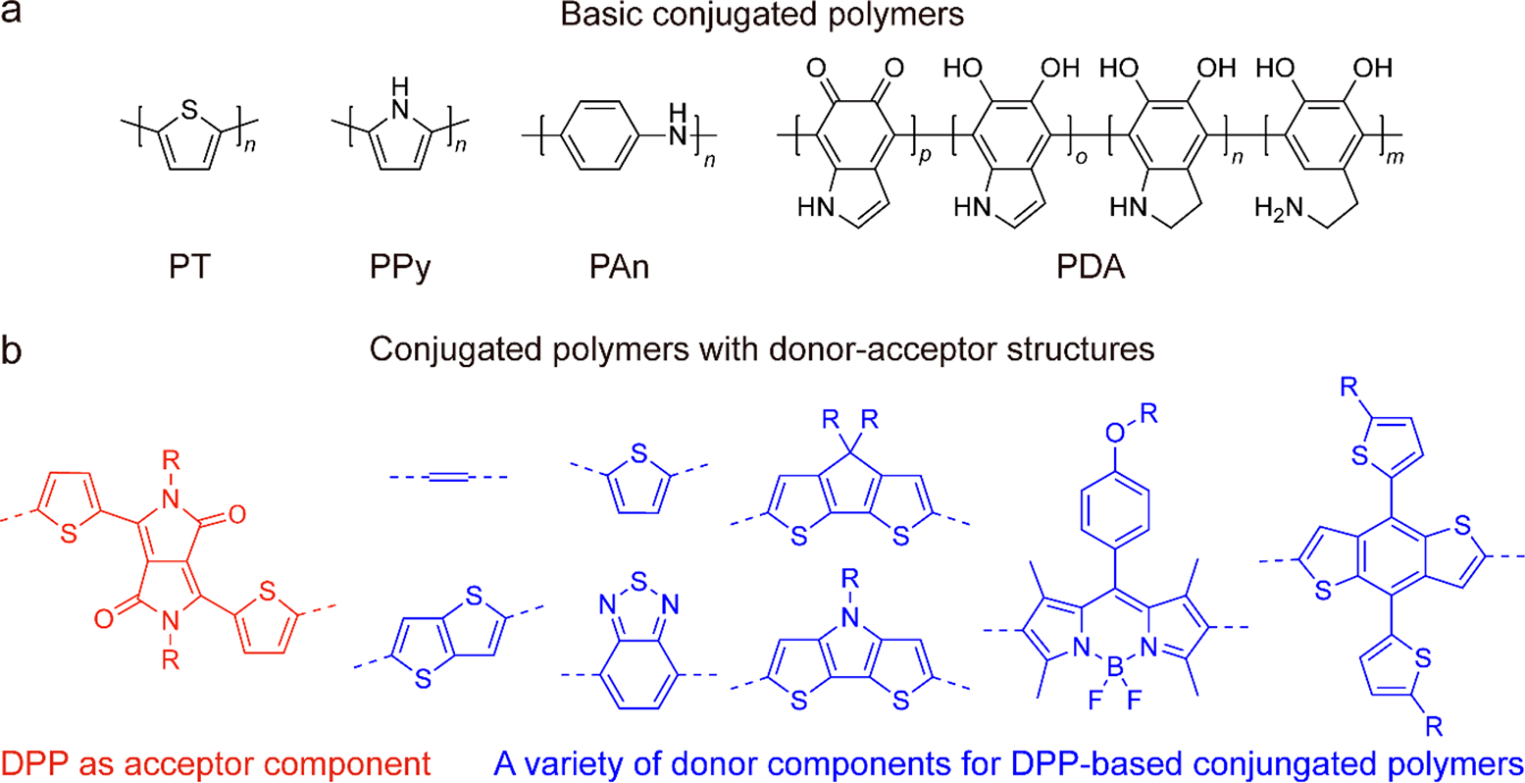
Summary of polymer-based photothermal materials. (a) Basic conjugated polymers as photothermal materials. Their monomers can be treated as the basic components of the conjugated polymers with D-A structures. (b) Conjugated polymers with donor–acceptor structures as photothermal materials. Reprinted with permission from ref (102). Copyright 2020 American Chemical Society.

Organic polymers with conjugated structures have emerged as a new category of photothermal nanomaterials because of their versatile molecular designs, strong absorption of NIR light, high light-to-heat conversion efficiencies, and good biocompatibility.^98^ Similar to the case of carbon-based nanomaterials, the absorption capabilities of conjugated polymers in the visible and NIR regions stem from the nonradiative relaxation of their rich delocalized π electrons. Various polymer photothermal nanomaterials have been strategically designed on the basis of most basic conjugated structures, such as polythiophene (PT), polypyrrole (PPy), polyaniline (PAn), and polydopamine (PDA) (Figure 8a).^20,102,333^ Through the close stacking of their monomeric units, the intermolecular collisions are strengthened in the formed polymers. The construction of conjugated polymers can partly quench molecular fluorescence and enhance nonradiative relaxation, resulting in efficient photothermal conversion.^98^ Moreover, donor–acceptor (D-A) strategies have been proposed to develop a new series of conjugated polymers for further extending light absorption and improving light-to-heat conversion efficiencies.^334−338^Figure 8b presents a typical class of polymer-based photothermal materials with D-A structures consisting of diketopyrrolopyrrole (DPP) as an acceptor and a series of electron-donating polymers as donors.^102^ While a conjugated polymer serves as a donor for absorbing light, another polymer accepts the excited electrons from the conjugated polymer and further releases them through nonradiative decay. The polymer as an acceptor needs to have a Fermi level that is lower than the excited state of the donor. The bandgap of these D-A structures can be facilely modulated by tuning the ratio and strengths of the donor and acceptor units.^339^ When the D-A structures are activated under external light irradiation, intramolecular charge transfer is induced along the backbone. The radiative recombination of electrons and holes can be consequently suppressed, which enhances heat generation. This D-A strategy has been employed to prepare conjugated polymer nanoparticles for the harvesting of NIR light.^334^ The photothermal conversion efficiency as high as 62.3% has been achieved by introducing a porphyrin-pyrene pendant as an extra light-harvesting unit. The same research group has further demonstrated a new type of biodegradable conjugated polymer made of two acceptors and one donor.^340^ This A-D-A structure not only contributes to nearly complete fluorescence quenching but also brings about a narrow energy gap. The photothermal conversion efficiency has therefore been improved to 82%.

**Figure 9:**
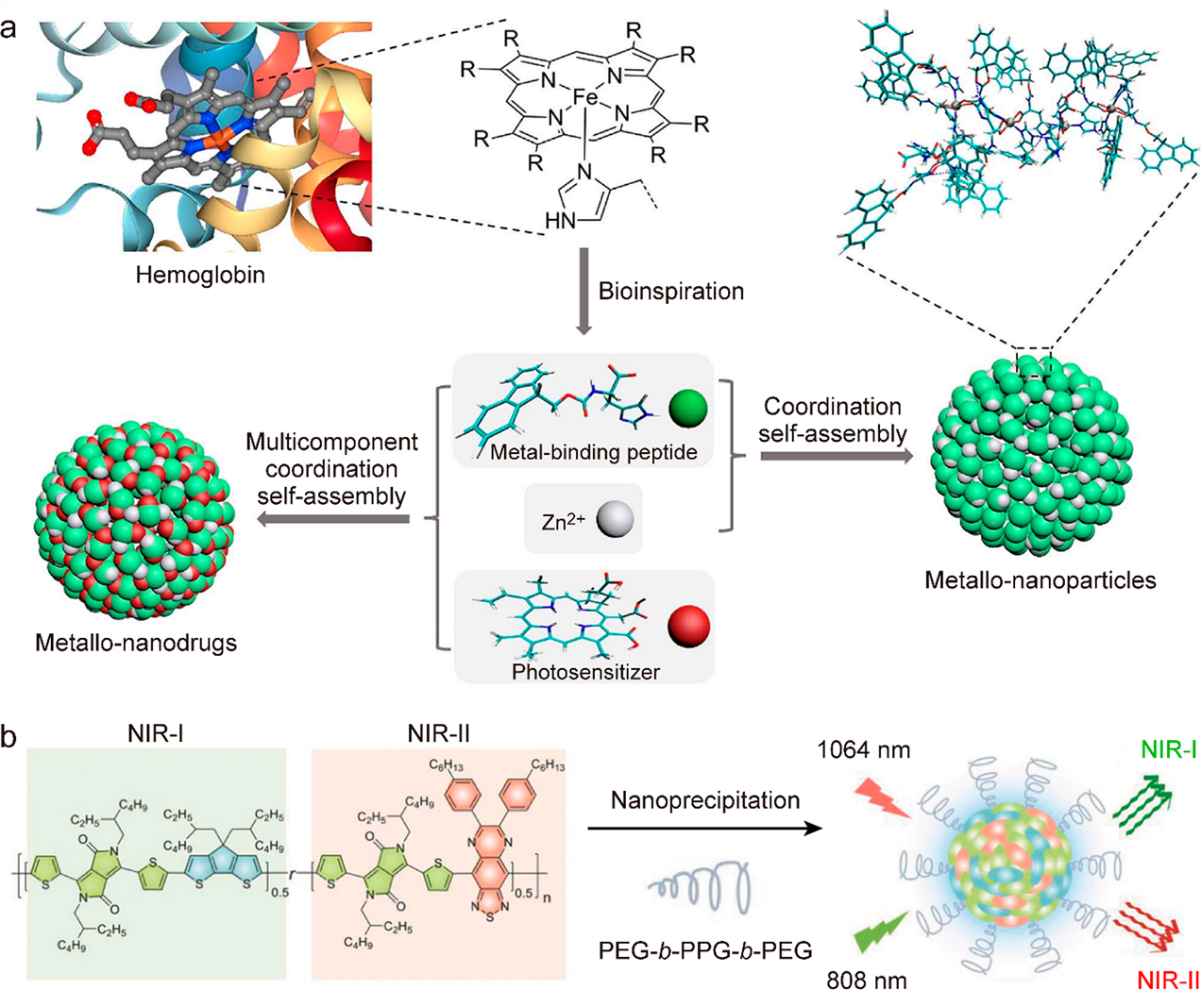
Supramolecular assembly of polymer-based photothermal materials. (a) Schematic illustrating the multicomponent coordination assembly of metallo-nanodrugs. Reprinted with permission from ref (349). Copyright 2018 American Chemical Society. (b) Design and synthesis of a dual-NIR-window absorbing photothermal nanoagent. Reprinted with permission from ref (353). Copyright 2018 Wiley-VCH.

To further improve the photothermal conversion efficiency and enhance the stability of conjugated polymers, supramolecular assemblies have been extensively developed to fabricate new polymer-based photothermal materials.^341−346^ The assembly is distinguished from traditional molecular structural design and based on flexible noncovalent intermolecular interactions, such as van der Waals interaction, hydrogen bonding, and electrostatic interaction.^37^ Similar to the aggregation-caused quenching effect, the intrinsic fluorescent emissions of molecules can be largely quenched in the supramolecular system, which results in the enhancement of heat generation.^347^ In addition, the material stability can be significantly improved through the formation of supramolecular structures. The monomeric molecules are trapped into supramolecular assemblies where only a few peripheral molecules can be affected by the outer environment.^21^ The isolation of the photothermal molecules can effectively avoid decomposition or oxidation, thereby maintaining their photophysicochemical properties. The photothermal conversion of unstable molecules can be greatly improved. Due to these unique advantages of supramolecular assembly, much effort has been dedicated to the exploration of new assembly methods and the promotion of the light-to-heat conversion performance.^348−352^ A multicomponent coordination self-assembly approach has recently been developed to synthesize supramolecular nanodrugs (Figure 9a).^349^ The assembled multicomponent nanoparticles exhibit well-defined spherical structures, uniform sizes, and robust colloidal stability. Moreover, the development of dual-peak absorbing photothermal nanoagents through the supramolecular assembly strategy has been demonstrated (Figure 9b).^353^ Two types of conjugated polymers with D-A structures are used for light absorption in both NIR-I and NIR-II windows. The photothermal conversion efficiencies of this dual-peak absorbing polymer are 44.9% and 43.4% at 808 and 1064 nm, respectively.

### 3.5 Two-Dimensional Nanomaterials

**Figure 10:**
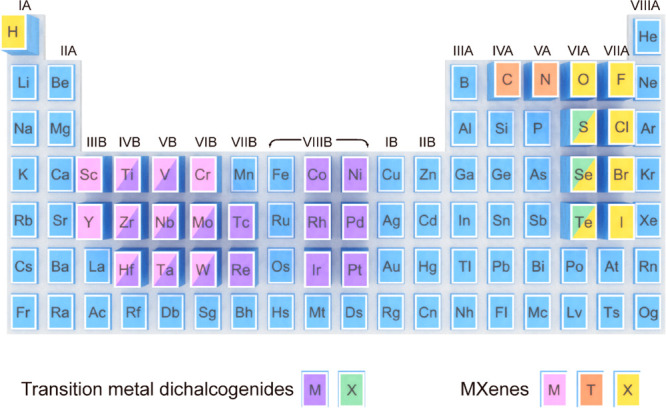
Periodic table showing the compositions of TMDCs and MXenes.

Since the discovery of single-layer graphene in 2004, there has been tremendous growth in the development of 2D nanomaterials with extraordinary physical and chemical properties.^354^ Various new types of 2D nanomaterials, including MXenes, MOFs, transition metal dichalcogenides (TMDCs), black phosphorus (BP), tellurene, and layered double hydroxides, have been delicately fabricated with appealing photothermal conversion properties.^22−24,355−358^ Unlike graphene or BP made of a single element, TMDCs and MXenes, as two big fast-growing families of 2D nanomaterials, have been rapidly expanded with a large variety of compositions (Figure 10).^359,360^ The versatile designability of 2D nanomaterials allows their photothermal performances to be facilely tailored and become competitive with other photothermal materials. 2D nanomaterials possess a typical layered structure. Their bandgaps can be simply tuned by adjusting the number of layers. The thickness-dependent bandgaps endow them with a wide absorption band. For instance, the bandgap of BP can be changed from 0.3 to 2 eV across a broad range from the visible to FIR region by controlling the layer number.^361,362^ As the thinnest materials ever known, 2D nanomaterials have the highest specific surface areas compared to zero-dimensional (0D), 1D, and bulk materials.^363^ The ultrathin structure can not only bring rapid response to light but also provide excellent in-plane electron mobilities to achieve high photothermal conversion efficiencies. MXenes, specifically Ti_3_C_2_, have been reported to possess a perfect light-to-heat conversion efficiency of 100%, showing the promising future of MXenes for photothermal applications.^364^

**Figure 11:**
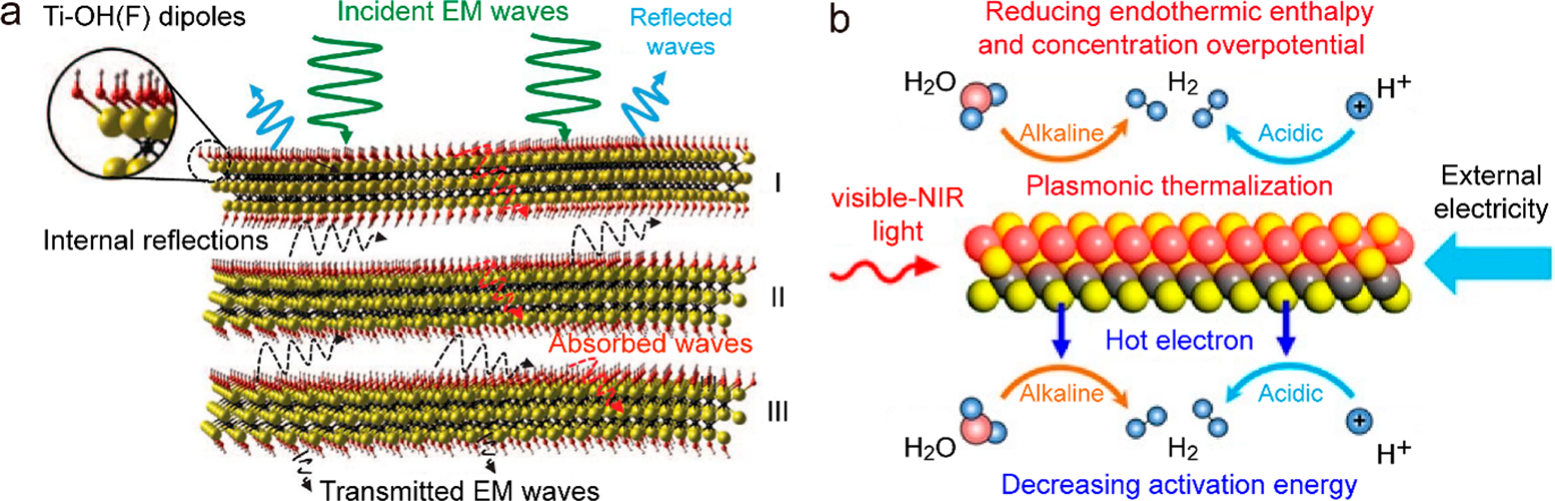
EMI shielding and LSPR effects in MXenes. (a) Proposed EMI shielding mechanism. Reprinted with permission from ref (371). Copyright 2016 American Association for the Advancement of Science. (b) Schematic illustration of the LSPR-induced photothermal and hot-electron effects. Reprinted with permission from ref (372). Copyright 2021 Wiley-VCH.

MXenes have become a rapidly rising star among 2D nanomaterials since the first reported synthesis of Ti_3_C_2_ in 2011.^360,365,366^ As carbides and nitrides of transition metals, MXenes have a general chemical formula of M_*n*+1_X_*n*_T_*x*_, where M represents an early transition metal (Sc, Ti, V, Cr, Y, Zr, Nb, Mo, Hf, Ta, W), X represents carbon or nitrogen, and T denotes the surface termination (OH, O, F, Cl).^367^ They can be synthesized by selective etching of their precursor ternary MAX phases, where A represents a group IIIA or IVA element.^368^ While numerous MXene compositions have already been prepared, many more have been predicted by computational methods. Because of the presence of transition metals, the free charge carrier densities of MXenes are on the order of 10^22^ cm^–3^ and the reported highest metallic conductivity^369,370^ reaches up to 20000 S cm^–1^. Based on their excellent metallic conductivity and layered structure, MXenes exhibit a strong electromagnetic interference (EMI) shielding effect (Figure 11a).^371^ Except for the immediately reflected waves from the surface, the remaining EM waves suffer from multiple internal reflections within the MXene flakes, leading to more absorption and an overall attenuation of EM waves. In addition to the EMI shielding effect, the abundant free charge carriers of MXenes also bring about LSPRs (Figure 11b).^372^ The plasmonic properties can be chemically tuned by altering the structure and type of the X and M sites, as well as the stoichiometry of the surface terminations.^39^ MXenes with various compositions support tunable plasmon resonances in the entire visible and NIR regions.^373^ The interband transitions of MXenes can also induce strong absorption in the UV region. These unique merits of MXenes are beneficial for efficient light harvesting and thermal energy generation.^23^

### 3.6 Hybrid Photothermal Nanomaterials

Hybridization is a simple strategy to obtain complementary and synergistic properties from each component. The formation of hybrid nanostructures composed of different photothermal materials has been widely employed to achieve enhanced light absorption and better photothermal capabilities.^49,374−380^ Metal nanostructures incorporated with plasmonic semiconductors or other metals can create a dual-plasmonic system with multiple plasmon resonance modes for broadening the NIR absorption band.^34^ The decoration of semiconductors with Pd nanoparticles as metallic active sites is usually used for improving the photothermal catalytic activities.^146^ The introduction of Ag nanoparticles to other photothermal materials can give rise to a greatly synergistic antibacterial and photothermal performance.^381^ To overcome the disadvantages of poor photostability and high photoluminescence emission, conjugated polymers are integrated with carbon-based materials or plasmonic metals.^99^ On the other hand, the modification of these inorganic materials with polymers can also minimize their long-term toxicity. Consequently, the hybrid design provides a promising way to engineer photothermal materials with synergistic optical properties for targeted applications.

## 4 Probing of Photothermal Heat Generation

**Figure 12:**
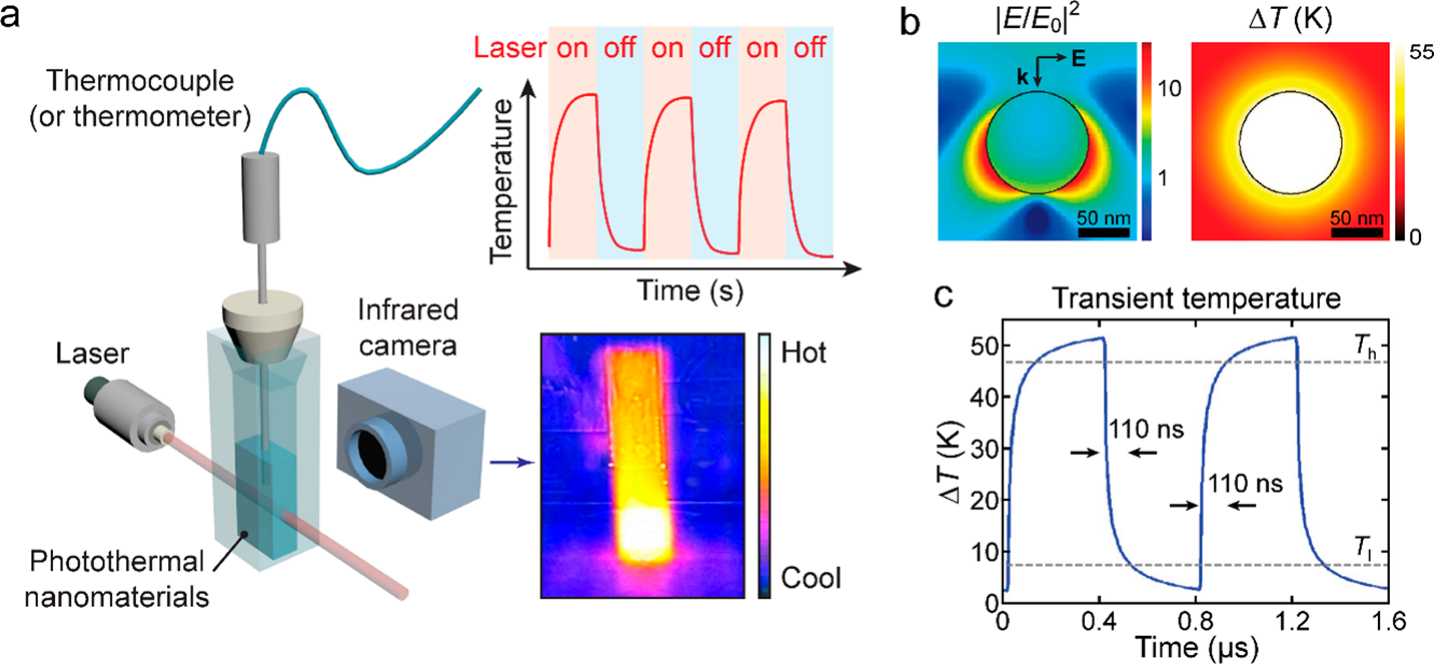
Macroscopic and microscopic temperature measurements. (a) Schematic diagram of typical photothermal characterization with traditional thermometric techniques. The heating and cooling processes are controlled by switching on and off the laser and monitored with mercury thermometers or thermocouples. The macroscopic temperature distribution can be imaged with an IR camera. (b) Simulation of the photothermal effect of a Au nanosphere (radius, 50 nm) placed in water under an incident light at 530 nm, tuned to the dipole plasmon resonance wavelength. Left: electric field intensity profile normalized to the incident field. Right: equilibrium distribution of the temperature increase. Reprinted with permission from ref (260). Copyright 2010 American Chemical Society. (c) Simulated transient temperature increase of a Au nanosphere (radius, 50 nm) in water excited by on–off modulated light. *T*_h_ and *T*_l_ represent 90% and 10% of the temperature step, respectively, which define the rise and fall time of 110 ns. Reprinted with permission from ref (382). Copyright 2012 American Chemical Society.

Traditional thermometric techniques, such as mercury thermometers, thermocouples, and IR cameras, can only give macroscopic temperatures as a collective effect of photothermal nanomaterials and are usually limited to the measurements of bulk or surface temperatures with low spatial and temporal resolution (Figure 12a). These conventional thermometers are commonly used for monitoring the heating and cooling processes of photothermal nanomaterials irradiated by a continuous laser on the time scale of a few hundreds of seconds, from which the photothermal conversion efficiency can thereby be derived.^12,21,85,363^ However, photothermal conversion and the associated effects actually occur on much shorter time scales and in nanoscale dimensions that are beyond the reach of traditional thermometry.

Take plasmonic nanoparticles as an example. LSPR and plasmonic heating are intrinsically localized and involve ultrafast excitation. Electromagnetic and thermodynamic simulations show that the photothermal-induced heat is highly concentrated at the surface (<20 nm) of irradiated plasmonic nanoparticles (Figure 12b) and strongly depends on the size and shape of the nanoparticles.^260^ In addition, the local temperature increase can be extremely rapid (Figure 12c), reaching the nanosecond and even picosecond levels, as revealed in the pioneering works^383,384^ and a more recent work.^382^ This recent work shows the reshaping of individual Au nanoparticles triggered by transient photothermal heating with femtosecond and nanosecond laser pulses.

Accurate temperature measurement of nanomaterials is critical for understanding their photothermal properties, heat generation mechanisms, and roles in physical, chemical, and biological applications. Controlled local heating is essential in biomedical diagnosis and therapeutics, especially for killing cancer cells in tumors with temperature elevation above the physiological level (41–42 °C) and simultaneously preventing excessive injury to the surrounding healthy tissues or post-treatment inflammatory responses.^385,386^ In photothermal catalysis, the temporal scales of the plasmon-induced hot carrier generation, hot electron transfer, and thermalization processes are on the time scales of fs to sub-ns.^9,387^ Excessive local heating also poses problems for the stability of the nanomaterials themselves and for the proper functioning of the associated optoelectronic devices. Therefore, monitoring the local temperature distributions and transient temperatures of photothermal nanomaterials, with sufficient sensitivity and accuracy, is of general interest to a wide range of scientific communities, from fundamental materials science to chemical and medical applications. Consequently, nanothermometers and spectroscopic methods have emerged as alternative tools for temperature measurements with improved spatial, temporal and temperature resolutions.^388−391^

### 4.1 Heater–Thermometer Nanoplatforms

**Figure 13:**
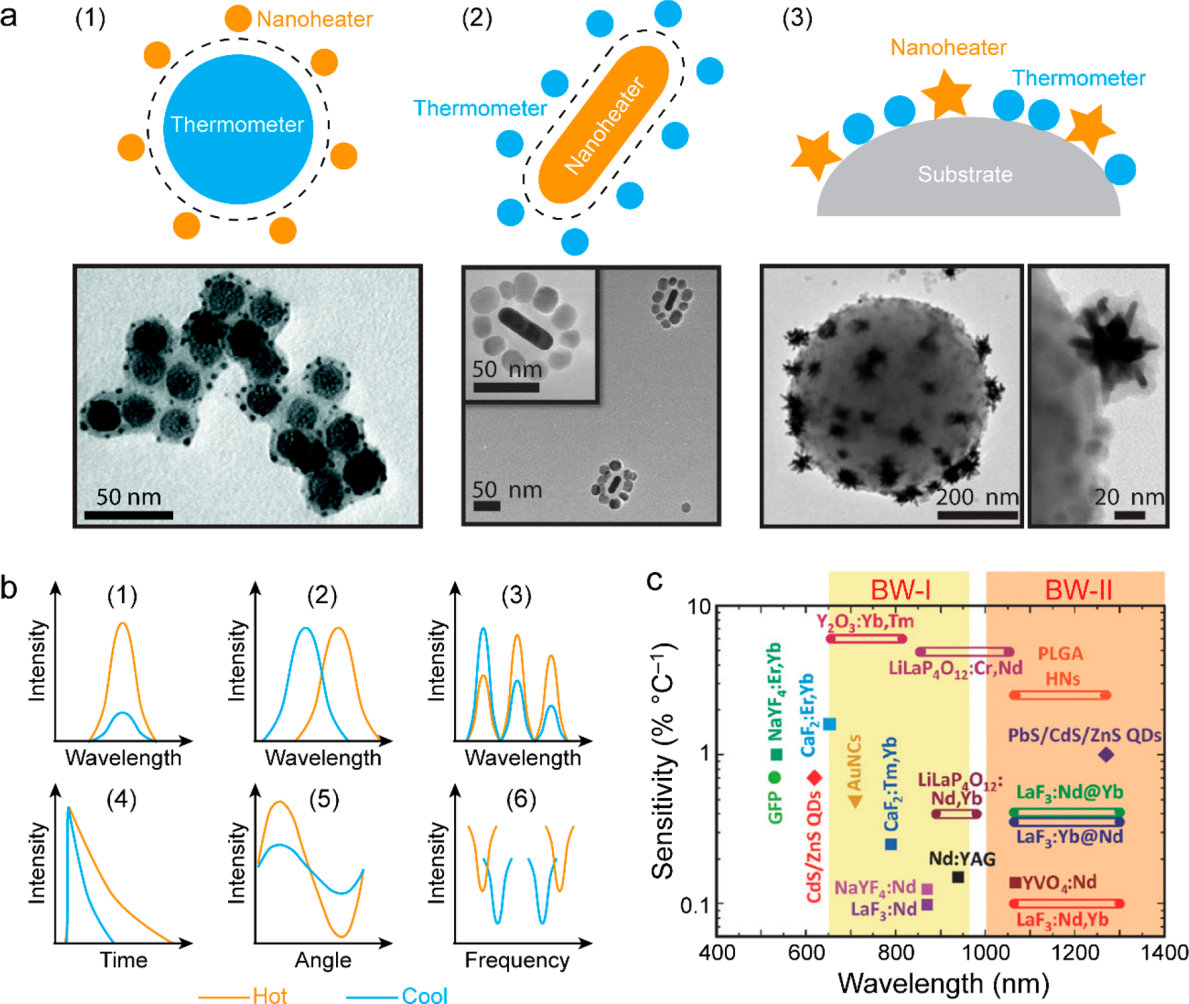
Nanothermometers for local temperature measurements. (a) Schematics (upper row) illustrating three different configurations for combining nanoheaters and nanothermometers and transmission electron microscopy (TEM) images (lower row) of representative hybrid nanostructures: (1) (NaYF_4_:Yb,Er up-conversion nanoparticle (UCNP))@(mesoporous silica (mSiO_2_))-(Au nanoparticle) core–satellite nanoassemblies. Reprinted with permission from ref (394). Copyright 2019 Royal Society of Chemistry. (2) (Au nanorod)-(NaGdF_4_:Yb,Er UCNP) core–satellite nanoassemblies. Reprinted with permission from ref (397). Copyright 2016 Wiley-VCH. (3) Polystyrene beads attached with Au nanostars and CaF_2_:Nd^3+^,Y^3+^ UCNPs. Reprinted from ref (400). Copyright 2019 Ivyspring International Publisher under the CC BY 4.0 license http://creativecommons.org/licenses/by/4.0/. (b) Illustrations showing temperature-sensing strategies relying on (1) emission intensity, (2) peak position, (3) emission intensity ratio, (4) emission lifetime, (5) fluorescence polarization anisotropy, (6) electron spin resonance or ODMR. (c) Thermal sensitivities of different luminescent nanothermometers and their spectral operation ranges. Reprinted with permission from ref (391). Copyright 2017 Wiley-VCH.

Heater–thermometer nanoplatforms refer to hybrid nanostructures consisting of photothermal nanomaterials (nanoheaters) and nanothermometers. Such hybrid nanostructures enable simultaneous heating and nanothermometry. A straightforward approach is to simply mix nanoheaters and nanothermometers in a matrix, which has been employed to compare the heating and absorbing efficiency of Au nanoparticles with different geometries,^392^ as well as to monitor plasmonic heating in a subtissue.^393^ Nevertheless, direct decoration of nanothermometers on the surface of nanoheaters, or *vice versa*, is highly desired for local temperature measurements with higher accuracy. Figure 13a illustrates three configurations that have been experimentally proven to work: (1) thermometer–heater core–satellite nanostructures,^394−396^ (2) heater–thermometer core–satellite nanostructures,^397−399^ and (3) codeposition of heaters and thermometers on a sub-microscale substrate.^400^ These heater–thermometer nanoplatforms can be constructed using different photothermal nanomaterials and nanothermometers as building blocks.

The selection of nanothermometers can cover a series of luminescent temperature-responsive probes, including fluorescent small molecules,^401^ fluorescent proteins,^402^ quantum dots (QDs),^403^ lanthanide-ion-doped UCNPs,^404^ vacancy-containing nanodiamonds,^405−407^ carbon dots,^408,409^ and polymeric nanoparticles.^409^ The operation of these nanothermometers mostly relies on the temperature-dependent fluorescence properties (Figure 13b), for example, the emission intensity, emission peak shifting, spectral ratio between the different fluorescence bands, lifetime, polarization anisotropy, and electron spin resonance or optically detected magnetic resonance (ODMR). All of these signals allow for the real-time and noninvasive monitoring of local temperatures. Briefly, the fluorescence intensity of small organic molecules generally decreases as the temperature is increased because of the thermal quenching effect.^401^ Some specific molecules, such as green fluorescent proteins and organic dyes, possess temperature-dependent fluorescence polarization anisotropy; that is, the ratio of the emission intensities collected under different polarization states can vary with the environmental temperature.^402^ Aggregation-induced emission (AIE) molecules have also been demonstrated to display temperature-responsive properties in terms of spectral position, fluorescence intensity, and fluorescence lifetime.^410^ Most semiconductor QDs exhibit temperature-dependent spectral shifts and lifetime variations because of their bandgap changes with temperature.^403^ UCNPs with specific thermally coupled energy-level pairs, such as Er^3+^ (^2^H_11/2_ and ^4^S_3/2_), Nd^3+^ (^4^F_5/2_ and ^4^F_3/2_), and Eu^3+^ (^5^D_1_ and ^5^D_0_), are commonly used for temperature sensing by monitoring the intensity ratio between the two related fluorescence bands, also known as ratiometric optical nanothermometry.^389,404^ Fluorescent nanodiamonds for temperature sensing rely on the spin resonances of the nitrogen-vacancy center and their temperature-dependent shifts in the ODMR spectrum at microwave frequencies.^405−407^ An important figure of merit for the comparison of nanothermometers is the relative thermal sensitivity, which is defined as the rate of change in the temperature-sensitive parameter with temperature, , where *Q* denotes the temperature-sensitive parameter (intensity, lifetime, ratio) and *T* denotes temperature. *S*_r_ is comparable between different systems with the consistent unit of K^–1^ or % K^–1^. Figure 13c provides a brief summary of the thermal sensitivities and spectral operation ranges of different luminescent nanothermometers, together with the first and second biological windows (BW-I and BW-II) where the absorption and scattering of biological tissues is minimal.^391^ Comparison of other factors, such as the thermal accuracy, sensor size, and thermal and special resolution, and more detailed discussion about different nanothermometers have been summarized in a few recent review articles.^386,388−391^

**Figure 14:**
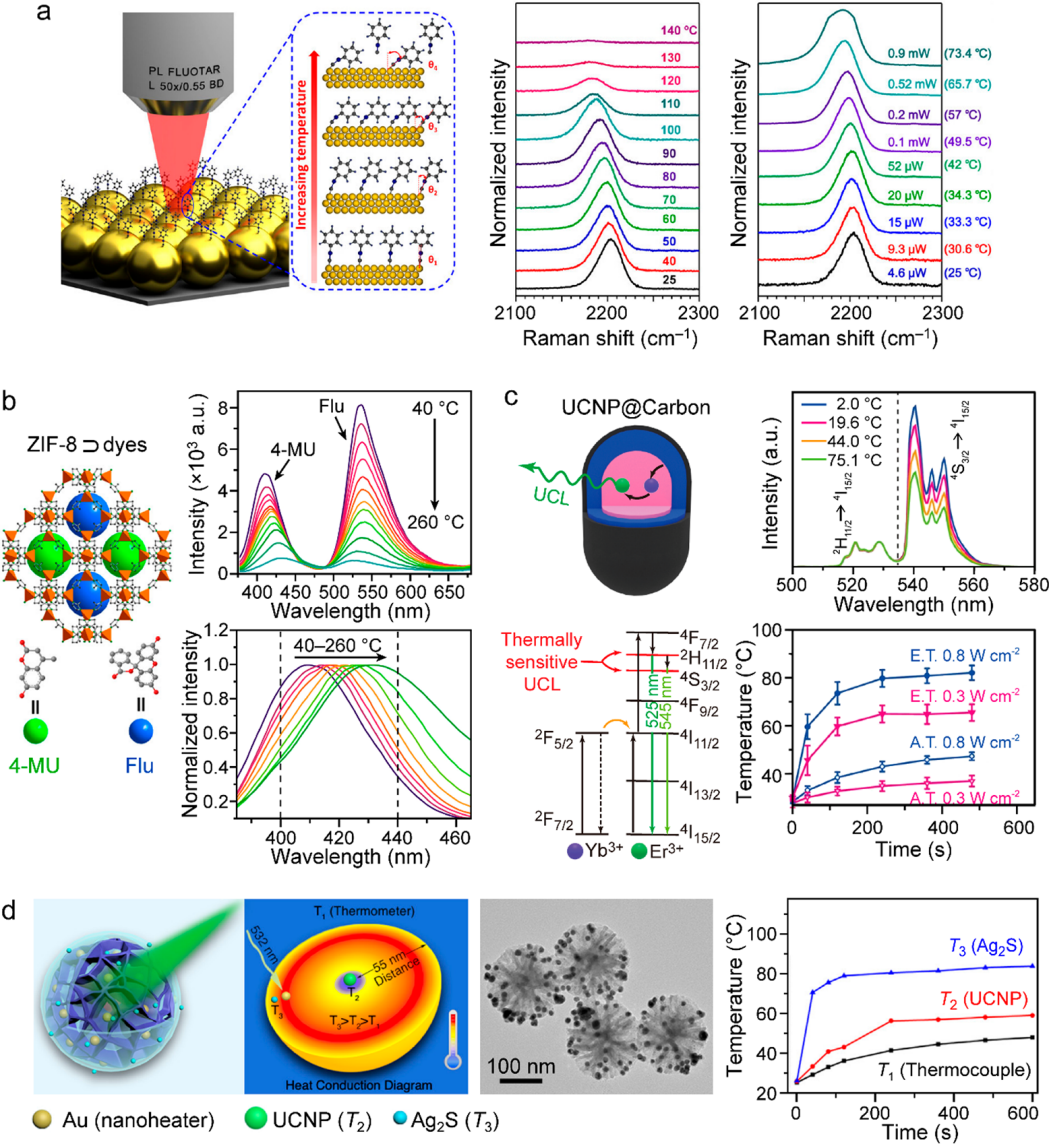
Heater–thermometer nanoplatforms. (a) Schematic (left) showing the temperature-dependent orientation change of phenyl isocyanide molecules on the surface of Au nanoparticles, and resultant temperature- (middle) and laser power-dependent (right) surface-enhanced Raman scattering (SERS) spectra of the N≡C stretching vibrations. Reprinted with permission from ref (411). Copyright 2018 American Chemical Society. (b) Dye-loaded ZIF-8 for temperature sensing based on the luminescent intensity ratio and emission peak shift. Reprinted with permission from ref (414). Copyright 2021 American Chemical Society. (c) Core@shell NaLuF_4_:Yb,Er@NaLuF_4_ UCNPs coated by carbon for photothermal heating and temperature sensing. Upper left: schematic of the UCNP@C nanoparticles. Lower left: energy diagram of the thermally sensitive UCL process. Upper right: emission spectra of the UCNP@C nanoparticles at different temperatures by external heating. Lower right: temperature evolution of the UCNP@C nanoparticles in an aqueous dispersion under irradiation with a 730 nm laser at 0.8 and 0.3 W·cm^–2^. A.T.: apparent temperature recorded by a thermal camera. E.T.: eigen temperature calculated by the UCL spectra. Reprinted from ref (396). Copyright 2015 Springer Nature under the CC BY 4.0 license http://creativecommons.org/licenses/by/4.0/. (d) Multilayer NaYbF_4_:2%Er@NaYF_4_@mSiO_2_@Au@SiO_2_@Ag_2_S nanoparticles. From left to right: schematic illustrating the multilayer structure, heat conduction diagram under internal heating, TEM image of the multilayer nanoparticles, and temperature evolution (*T*_1_: apparent temperature of the solution measured by a thermocouple; *T*_2_: core temperature measured by the UCNPs; *T*_3_: outmost layer temperature measured by the Ag_2_S QDs). Reprinted with permission from ref (415). Copyright 2020 American Chemical Society.

With advances in surface functionalization and assembly techniques, heater–thermometer nanoplatforms with different compositions and various configurations have been experimentally achieved. Plasmonic nanoparticles are the most commonly used nanoheaters because of their high photothermal conversion efficiencies and ease of surface functionalization. For example, local temperature measurements of Au nanoparticles have been demonstrated by monitoring the Raman spectra of phenyl isocyanide molecules functionalized on the top surface of the Au nanoparticles (Figure 14a).^411^ Since the molecules take a more tilted angle to the Au surface as the temperature is increased, the Raman peak associated with the N≡C stretching vibrations exhibits sensitive shifts with temperature. This phenomenon has further been used to determine CO photothermal desorption from the Au surface at ∼62 °C and to track the local temperature variations of a single living cell. Similarly, Au nanoparticles have been combined with molecular beacons,^412^ nanodiamonds,^413^ and UCNPs,^400^ respectively, for extracellular or intracellular local heating and temperature monitoring. Multimode temperature readout has been realized by encapsulating two types of luminescent dyes (4-Mu and Flu) into the pores of a nanoscale MOF zeolitic imidazolate framework-8 (ZIF-8) and measuring the emission intensity ratio and maximum emission wavelength (Figure 14b).^414^ The thermal sensitivity can reach *S*_r_ = 0.62% K^–1^ at 240 °C; albeit, the photothermal conversion effect is not significant in this system. UCNP@C core@shell nanoparticles have been successfully prepared for simultaneous photothermal heating and nanothermometry (Figure 14c).^396^ Accurate PTT has been demonstrated by use of the photothermally active carbon nanoshells as heaters and the UCNPs for the real-time monitoring of microscopic temperatures, so as to reduce injury on the normal tissues during photothermal treatment. The local temperature calculated from the up-conversion luminescence (UCL) spectra is 10–15 °C higher than the macroscopic temperature recorded by a thermal camera, indicating the important role of accurate nanothermometry in biomedical diagnosis and therapeutics. The same group has further developed a complex multilayer structure in the form of NaYbF_4_:2%Er@NaYF_4_@mSiO_2_@Au@SiO_2_@Ag_2_S (Figure 14d), where the Au nanoparticles play the role of nanoheaters, whereas both the NaYbF_4_:2%Er@NaYF_4_ UCNPs and the Ag_2_S QDs function as nanothermometers.^415^ In the photothermal measurements, the Ag_2_S nanothermometers, which are designed to be closest to the nanoheaters, experience rapid heating and reach an equilibrium temperature rapidly. More interestingly, the measured temperatures follow the order Ag_2_S > UCNP > thermocouple, which matches well with the order of the distance between the Au nanoparticles (nanoheaters) and the different thermometers. Although there are not many hybrid systems that can perform effective local heating and local temperature detection at the same time, such efforts are beneficial for the development of heater–thermometer nanoplatforms with accuracy, sensitivity, and resolution, as well as for understanding the heating mechanisms of different photothermal nanomaterials. However, it is worth noting that the introduction of a nanothermometer near a nanoheater might affect the optical properties of both sides, including the heating efficiency of the nanoheater and the temperature calibration curve of the nanothermometer. Careful calibration is therefore especially crucial for these new systems.^416^

### 4.2 Non-nanoprobe Thermometry

Non-nanoprobe thermometry refers to the direct measurement of the local temperatures or steady-state spatial temperature gradients of photothermal nanomaterials without introducing any nanothermometers. It mainly relies on various spectroscopies. The simplicity of local temperature control allows for an in-depth study of the dissipation and heat transfer mechanisms at the nanoscale.

**Figure 15:**
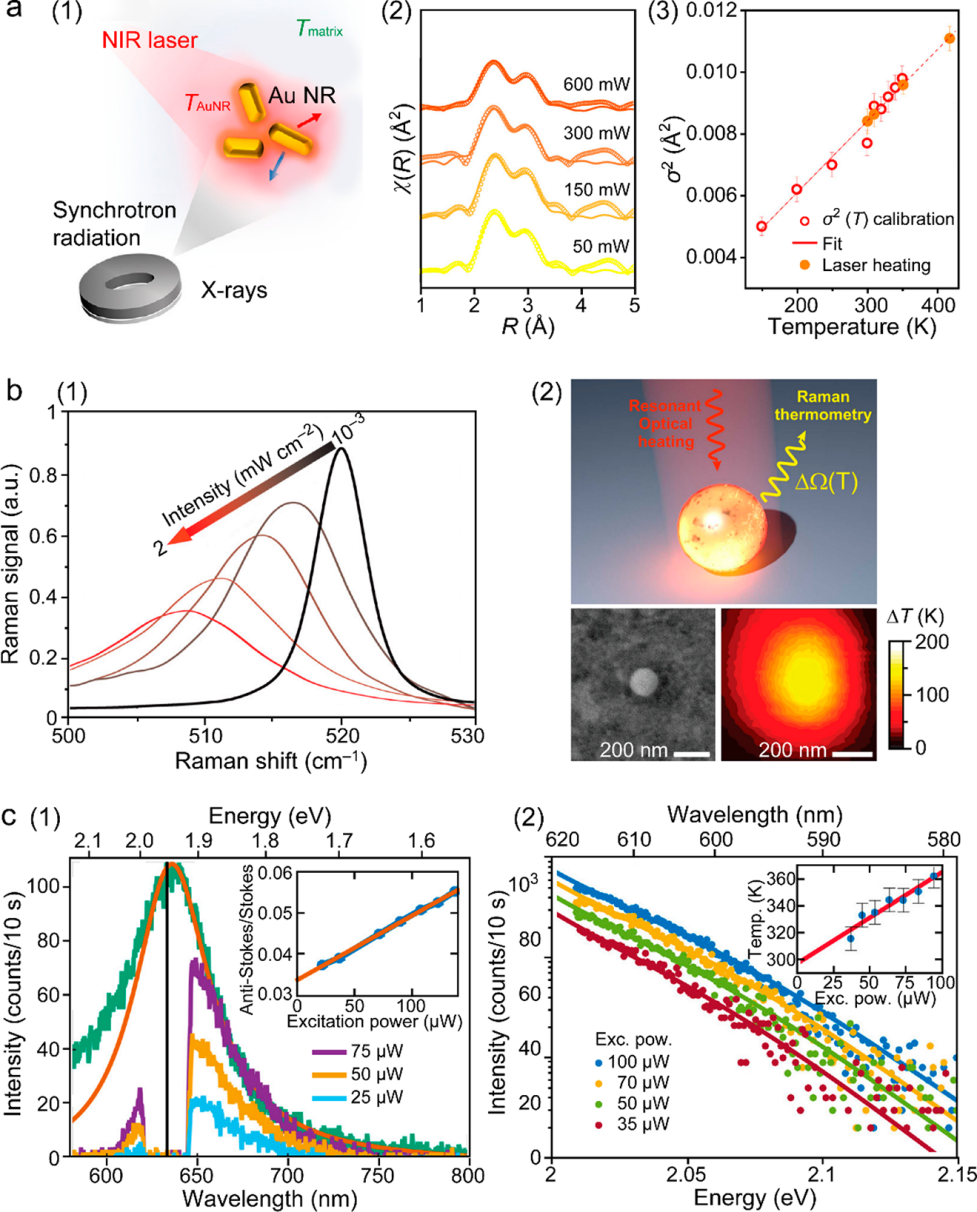
Non-nanoprobe thermometry. (a) Local temperature measurement of Au nanorods with X-rays. (1) Schematic illustrating the photothermal excitation of Au nanorods with visible-NIR light and the local temperature detection with X-rays. (2) Fourier transforms of the X-ray absorption fine structure spectra of Au nanorods under an NIR laser at different powers. (3) Debye–Waller factor parameters (σ^2^) of the Au nanorods as a function of temperature. The calibration curve was first obtained for a set of temperatures (open circles) and then used for the temperature determination under different laser excitation conditions (solid circles). Reprinted with permission from ref (417). Copyright 2021 American Chemical Society. (b) Local temperature measurement of individual Si nanoparticles by Raman spectroscopy. (1) Experimental Raman spectra for a spherical Si nanoparticle with a diameter of 350 nm on a glass substrate. (2) Schematic illustration, SEM image, and reconstructed 2D temperature map of a single Si nanoparticle under laser irradiation at an intensity of 2 mW μm^–2^. Reprinted with permission from ref (420). Copyright 2017 American Chemical Society. (c) Local temperature measurement of Au nanorods by anti-Stokes Raman spectroscopy. (1) Luminescence emission spectra of a single Au nanorod under 532 nm excitation (green) and 633 nm excitation at three different powers (purple, orange, and blue). The red curve is the surface plasmon profile extracted from the green curve using the Lorentz function. The inset shows the anti-Stokes-to-Stokes ratio as a function of the excitation power, overlapped with a linear fit in red. (2) Anti-Stokes Raman intensity of a single Au nanorod at different irradiation powers. The inset shows the extracted temperature at each power. Reprinted with permission from ref (427). Copyright 2018 American Chemical Society under the CC-BY-NC-ND 4.0 license http://creativecommons.org/licenses/by/4.0/.

X-ray can be used as the probe for local temperature measurements. *In situ* local temperature measurements of the photoinduced heating in single and hybrid Au nanoparticles have been demonstrated using extended X-ray absorption fine structure spectroscopy (EXAFS).^417^ The temperature dependence of the structural and vibrational local parameters of Au nanoparticles can be quantified through the Fourier analysis of the EXAFS spectra, which contain information about photoexcited electrons that interact with the atomic local environment of the scattered atoms (Figure 15a). Although the measurements are time-consuming (∼2 h per sample per experimental condition),^417^ the work reveals significant nanoscale thermal gradients, i.e., large variations of temperature within a small volume. In principle, this method allows for the measurements of the heating at the nanoscale for other types of nanoparticles under hyperthermia exposure. In addition, a scheme based on an optical pump and an X-ray diffraction probe has been developed to directly measure the photothermal effects on the lattice of TiN nanoparticles dispersed in water.^418^ The lattice temperature of the TiN nanoparticles, from room temperature up to as hot as 175 °C, can be measured by calibrating transient X-ray diffraction data against static powder diffraction measurements at elevated temperatures. The heat capacity of the TiN nanoparticles has further been studied. The temporal dynamics of heat transfer from the TiN nanoparticles to the solvent is found to be on a time scale of 0.4–1 ns. This technique has also been employed for the internal temperature measurements of magnetic nanoparticles heated by a high-frequency magnetic field.^419^ However, these X-ray-based thermometry techniques typically require powdered samples under inert gas atmosphere, which therefore limits their applications in complicated systems.

Raman spectroscopies, both of the Stokes and anti-Stokes components, can be utilized for measuring the local temperatures of nanomaterials. In a work reported in 2017, a single Si nanoparticle simultaneously acts as a heater and a thermometer and integrates effective photoinduced heat generation and broad-range temperature sensing.^420^ A photoinduced temperature up to 900 K is observed from the Si nanoparticle deposited on a glass substrate under the radiation of a typical 633 nm laser at moderate intensities (<2 mW μm^–2^). The temperature sensing is accomplished simply by monitoring the intensity variation of the characteristic 520 cm^–1^ (Stokes) Raman mode in silicon (Figure 15b), which is associated with the anharmonic effect in the lattice vibrations and inherently thermally sensitive. However, this method of Raman nanothermometry has very limited applicability to other materials. For most materials, anti-Stokes Raman emission signals are more sensitive to temperature variations than Stokes components.^421^ The intensity ratio between the anti-Stokes and Stokes Raman peaks has a temperature-dependent term , where ν_m_ is the vibrational frequency of the excited state. The anti-Stokes to Stokes intensity ratio is therefore commonly used as a more general approach for direct local temperature measurements.^422−426^ However, since the anti-Stokes signals are usually very weak, a careful calibration is crucial for accurate intensity measurements. An alternative method has therefore been developed to overcome this problem. In the method, the anti-Stokes luminescence emissions rather than the anti-Stokes to Stokes intensity ratio are used to obtain the absolute temperature of individual Au nanorods (Figure 15c).^427^ Without the need for precalibration, the temperatures of the Au nanorods can be determined *in situ* with an accuracy of 6% (4 K) by recording the anti-Stokes spectra of the individual nanorods with an acquisition time of 3 min (Figure 15c (1)). Furthermore, the accuracy can be improved to better than 2% by performing this single-particle Anti-Stokes emission measurement at different irradiation powers (Figure 15c (2)).^427^ Taken together, all the works discussed above about the thermometry for photothermal conversion suggest that fast and accurate transient local temperature probing techniques have remained a challenge.

## 5 Applications

Together with the investigation on photothermal conversion mechanisms and the advancement of nanofabrication techniques, immense effort has been made to extend the applications of photothermal nanomaterials. Nanomaterials with efficient solar-to-heat conversion provide a promising solution to satisfy the ever-increasing water and energy demand. Pure water can be produced from seawater and wastewater through solar water heating and evaporation, which can be accelerated by suitable photothermal nanomaterials. The generated hot vapor during solar evaporation can be used for sterilizing, driving mechanical work, and even producing electricity. Photothermal conversion by nanomaterials has also been employed in other physical (laser printing, photothermal manipulation), chemical (photothermal catalysis), and biological (PTT, drug delivery, bacterial inhibition) applications. In this section, we will provide an overview on the recent developments in the applications of photothermal nanomaterials. Some of the most representative examples are highlighted to show the advantages of nanomaterials in these photothermal applications.

### 5.1 Solar Thermal Water Heating

**Figure 16:**
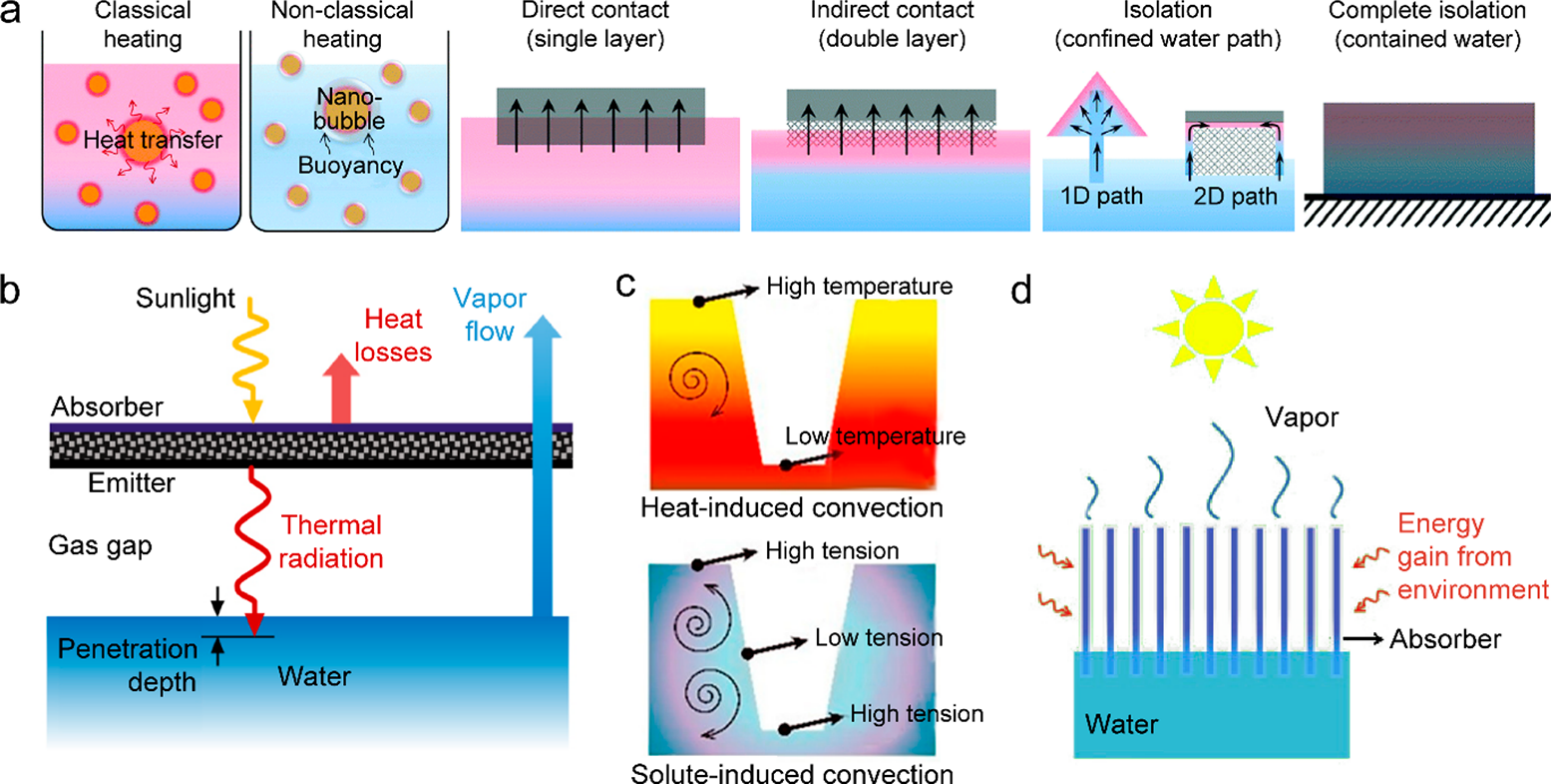
Solar thermal water heating and evaporation. (a) Schematics of the different types of photothermal systems for water heating and evaporation. Reprinted with permission from ref (51). Copyright 2018 Royal Society of Chemistry. (b) Noncontact radiative transfer from the solar absorber to the water. Reprinted with permission from ref (441). Copyright 2018 Cooper et al., published under the CC BY 4.0 license http://creativecommons.org/licenses/by/4.0/. (c) Marangoni-effect-accelerated water flow and -enhanced solar evaporation. Reprinted with permission from ref (465). Copyright 2021 American Chemical Society. (d) Environmental-energy-enhanced solar evaporation. Reprinted with permission from ref (448). Copyright 2018 under Elsevier user license.

Owing to the development of various photothermal materials, new solar thermal systems have emerged for efficient water heating and vapor generation. Because of the vast number of works in this area, representative works on solar thermal water heating and evaporation are summarized in Table 2. They serve as references for researchers who are interested in this area. Photothermal water heating systems can be categorized into different types according to the relative position of the solar-absorbing material and the water medium (Figure 16a).^7,51^ (1) When the solar absorbers are dispersed in water, the photothermal energy collected by the absorbers will transfer to water through classical (global heating) and nonclassical (nanobubble generation) mechanisms. Such volumetric systems, also known as nanofluids, usually employ well-dispersed plasmonic nanoparticles or carbon-based nanomaterials as the solar absorbers.^428−435^ Nanomaterials possess high surface-to-volume ratios and provide large surface areas for heat transfer to the surrounding medium. For classical heating, the solar absorbers act as the heat sources and transfer thermal energy to increase the temperature of the bulk fluid. For nonclassical heating, nanoparticles under concentrated sunlight absorb solar energy efficiently and raise their surface temperature above the boiling point of the fluid.^428,429^ A thin shell of water in direct contact with the nanoparticles transforms into vapor, forming nanobubbles. The complex made of the nanoparticle and the surrounding vapor moves to the liquid–air interface and releases the vapor. In this way, solar energy is primarily directed to the vaporization of water, with a much smaller fraction used for the heating of the bulk fluid. (2) When the solar absorbers are placed on the surface of the bulk water, the photothermal system is called the interfacial solar evaporation system.^436^ In contrast to the volumetric photothermal system, the interfacial system confines the solar heat and vapor generation at the water–air interface. The temperature of the underneath bulk water remains low during the evaporation process, minimizing the heat loss through the surrounding environment. The light-absorbing materials either directly float on water or are placed onto a porous layer with interconnected water-supplying channels.^7^ (3) Isolation systems with the solar absorbers separating from the bulk water have also been proposed to further suppress the conduction heat losses. To achieve a small contact area between the absorbers and the bulk water, confined 1D/2D water paths with a sufficient water-supply capability have been designed and fabricated.^437−439^ For example, monolithic carbon sponges with self-contained water have been demonstrated for vapor generation,^440^ where the photothermal system is completely isolated from the bulk water. In another example, a contactless vapor generation system has been proposed, with the absorber and the bulk water separated by a gas gap (Figure 16b).^441^ The absorber converts solar light to heat and consequently re-emits thermal IR radiation toward the bulk water. Vapor with temperatures as high as 133 °C is produced under one sun illumination in a nonpressurized system.

**Table 2:** Representative Works on Solar Thermal Water Heating

category	reference	photothermal material and device	water evaporation rate	evaporation efficiency	note
nanofluids	(428)	nanofluid of Au nanoshells		0.24	used for ethanol distillation
	(429)	nanofluid of Au nanoshells			used for sterilization
	(218)	nanofluid of Au-Ag nanoplates		0.785	
	(430)	Au, Ag, and Au-Ag blended nanofluids		0.3097	
	(431)	plasmonic nanofluid containing Au nanoparticles	6.27 kg m^–2^ h^–1^ under 10 sun irradiation	0.65	
	(432)	CuO and antimony-doped tin oxide composite nanofluid		0.925	
	(433)	reduced graphene oxide nanofluid		0.9693	exhibit better photothermal conversion efficiencies than the graphene and graphene oxide ones
	(434)	carbon black, graphene, and graphitized carbon black nanofluids	10.9 kg m^–2^ h^–1^ under 10 sun illumination	0.69	
	(435)	nanofluid of Fe_3_O_4_@CNTs		0.603	magnetic separation for recycling nanoparticles
carbon-based materials	(442)	carbon fibers	1.3 kg m^–2^ h^–1^ under 1 sun irradiation	0.8	salt-resistant ability based on the Donnan effect
	(443)	hierarchical Cu structures with Al_2_O_3_ coating and carbon black nanoparticles	1.31 kg m^–2^ h^–1^ under 1 sun irradiation	0.8	bioinspired structure
	(444)	carbon nanosheets	1.4 kg m^–2^ h^–1^ under 1 sun illumination	0.92	
	(445)	3D porous carbon foam	10.9 kg m^–2^ h^–1^ under 1 sun irradiation		wind used as an additional energy source
	(312)	carbon foam/exfoliated graphite layers	11 kg m^–2^ h^–1^ at 10 kW m^–2^ solar irradiance	0.85	
	(441)	contactless solar evaporator with porous reticulated vitreous carbon foam	2.5 L day^–1^ with daily insolation of 6 kW h m^–2^	0.25	antifouling and salt-rejecting structure
	(446)	CNT membrane	1.32 kg m^–2^ h^–1^ under 1 sun illumination	0.82	seawater desalination and wastewater purification
	(447)	CNTs/filter paper/cotton thread/polystyrene foam	1.42 kg m^–2^ h^–1^ under 1 sun illumination	0.812	isolated salt crystallization
	(448)	composite of cotton, cellulose, carbon black nanoparticles, and polystyrene foam	1.77 kg m^–2^ h^–1^ at 120 mW cm^–2^ solar irradiance	∼1	energy gained from the environment
	(314)	hollow carbon-nanotube aerogel	3.9186 kg m^–2^ h^–1^ under 3 sun illumination	0.868	
	(449)	ultrablack carbon aerogel	1.37 kg m^–2^ h^–1^ under 1 sun illumination	0.8751	implement CO_2_ activation to increase the hot-electron effect
	(450)	SiC/CNT coating		0.5094	used for anti-icing
	(451)	carbon foam deposited with layered BiInSe_3_	1.1 kg m^–2^ h^–1^ under 1 sun illumination		seawater desalination
	(452)	polyvinyl alcohol embedded with carbon black nanoparticles on a polyvinylidene fluoride membrane	0.5 kg m^–2^ h^–1^ at 700 W m^–2^ solar irradiance	0.538	saline water desalination
	(453)	poly methylmethacrylate coated with carbon black nanoparticles on polyacrylonitrile layer	1.3 kg m^–2^ h^–1^ under 1 sun illumination	0.72	seawater desalination with salt-resistant properties
	(454)	carbon-black-coated polyvinyl alcohol cloth	1.35 kg m^–2^ h^–1^ under 1 sun illumination	0.8475	continuous desalination with salt excretion
	(455)	umbrella architecture of carbon-coated fabric	9.05 kg m^–2^ h^–1^ under 1 sun illumination with natural wind		self-salt-cleaning
	(456)	3D-printed hydrogel decorated with carbon nanoparticles	4.12 kg m^–2^ h^–1^ under 1 sun illumination	0.921	inspired by the transpiration in trees
	(457)	aerogel of CNTs and hydroxyapatite nanowires	1.34 kg m^–2^ h^–1^ under 1 sun illumination	0.894	wastewater purification
	(458)	composite paper of CNTs and hydroxyapatite nanowires	14.31 kg m^–2^ h^–1^ under 10 sun illumination	0.928	seawater desalination and wastewater purification
	(459)	sponge with polydimethylsiloxane, CNTs, and cellulose nanocrystals	1.35 kg m^–2^ h^–1^ under 1 sun illumination	0.874	waste energy-to-electricity conversion by thermoelectric modules
	(437)	3D-printed structure with CNTs, graphene oxide, and nanofibrillated cellulose	1.25 kg m^–2^ h^–1^ under 1 sun illumination	0.856	
	(440)	monolithic carbon sponge	1.39 kg m^–2^ h^–1^ under 1 sun illumination	0.9	electricity generation by water evaporation-induced piezo-pyroelectric response
	(460)	hydrogel-coated graphite film	1.01 kg m^–2^ h^–1^ under 1 sun illumination	0.627	anticlogging coating prevents salt accumulation
	(461)	graphite/nonwoven film on nonwoven-wrapped polystyrene foam	34.8 kg m^–2^ h^–1^ at 30 kW m^–2^ solar irradiance	0.817	simultaneous generation of clean water and electricity
	(439)	carbonized mushrooms	1.475 kg m^–2^ h^–1^ under 1 sun irradiation	0.78	efficiency of 62% achieved for natural mushrooms
graphene-related materials	(462)	graphene oxide film	1.45 kg m^–2^ h^–1^ under 1 sun irradiation	0.94	solar desalination with four orders of salinity decrement
	(438)	graphene oxide	16.1 kg m^–2^ under 8 h outdoor solar irradiation	0.85	inspired by the natural transpiration process in plants
	(330)	graphene sheets	6.25 kg m^–2^ h^–1^ under 4 sun illumination	0.942	
	(463)	porous graphene	1.5 kg m^–2^ h^–1^ under 1 sun illumination	0.8	
	(464)	hierarchical graphene	1.4 kg m^–2^ h^–1^ at 5 kW m^–2^ solar irradiance	0.9	
	(320)	hydrophilically functionalized graphene	0.47 kg m^–2^ h^–1^ under 1 sun illumination	0.48	improved efficiency compared to chemically reduced graphene oxide
	(465)	hydrogel embedded with Ti_3_C_2_T_*x*_ MXene and reduced graphene oxide	3.62 kg m^–2^ h^–1^ under 1 sun illumination	0.91	enthalpy of vaporization lowered and Marangoni effect induced
	(466)	aerogel made of PPy-coated MnO_2_ nanowires and reduced graphene oxide	1.587 kg m^–2^ h^–1^ under 1 sun illumination	0.938	seawater desalination and wastewater purification
	(467)	reduced graphene oxide and silk fabric	1.48 kg m^–2^ h^–1^ under 1 sun illumination	1.02	energy gained from the environment
	(468)	conic arrays consisting of graphene-wrapped Fe_3_O_4_ nanoparticles	5.88 kg m^–2^ h^–1^ under 1 sun illumination		reconfigurable and magnetically responsive evaporator
wood materials	(469)	graphene-oxide-coated wood	14.02 kg m^–2^ h^–1^ under 12 sun illumination	0.83	
	(470)	flame-treated wood	1.05 kg m^–2^ h^–1^ under 1 sun illumination	0.72	water evaporation rate of 3.46 kg m^–2^ h^–1^ and efficiency of 0.81 under 3 sun illumination
	(471)	PPy-decorated wood	1.014 kg m^–2^ h^–1^ under 1 sun illumination	0.725	
	(472)	CuFeSe_2_-nanoparticle-decorated wood	6.6 kg m^–2^ h^–1^ under 5 sun illumination	0.862	
modified papers	(473)	CNT-modified filter paper	1.15 kg m^–2^ h^–1^ under 1 sun illumination	0.75	electricity extracted from the evaporation-induced salinity gradient
	(474)	PPy-modified paper	2.99 kg m^–2^ h^–1^ under 1 sun illumination		
	(262)	air-laid paper and Au-nanoparticle-based film	1.71 mg s^–1^ under 4.5 sun irradiation	0.778	
	(475)	Au nanoparticles supported on air-laid paper			
	(476)	Fe_3_O_4_ nanoparticles on air-laid paper	1.70 kg m^–2^ h^–1^ under 1 sun illumination	0.797	adjustable device by magnetic field
	(477)	Fe_3_O_4_ nanoparticles on air-laid paper	1.3 kg m^–2^ h^–1^ under 1 sun illumination	0.8	wastewater purification
cellulose-based materials	(33)	cellulose membrane deposited with Ti_2_O_3_ nanoparticles	5.03 kg m^–2^ h^–1^ at 5 kW m^–2^ solar irradiance	0.92	nearly 100% internal solar-thermal conversion efficiency
	(478)	membrane of mixed cellulose ester, ZnO nanorods, and Au nanoparticles	8.7 kg m^–2^ h^–1^ under 10 sun illumination		
	(479)	cellulose-based fabric/polystyrene	2.5 L m^–2^ day^–1^ under 1 sun illumination	0.22	salt rejection
gels	(480)	bilayered hydrogel	11.33 kg m^–2^ h^–1^ under 1 sun illumination		
	(481)	3D-printed hydrogel decorated with Fe_3_O_4_ nanoparticles	5.12 kg m^–2^ h^–1^ under 1 sun illumination		superior light absorption properties and rapid capillary-driven water transport
	(482)	hydrogel made of reduced graphene oxide and Ti_3_C_2_T_*x*_ MXene	2.09 kg m^–2^ h^–1^ under 1 sun illumination	0.935	reduced vaporization enthalpy
	(483)	hierarchically nanostructured gel made of polyvinyl alcohol and PPy	3.2 kg m^–2^ h^–1^ under 1 sun illumination	0.94	reduced vaporization enthalpy
	(484)	plasmonic aerogel based on biofoam and Au nanorods	0.32 mg cm^–2^ s^–1^ at a power density of 5.1 W cm^–2^	0.763	
	(485)	bilayer aerogel of MoS_2_ nanosheets and bacterial nanocellulose	6.15 kg m^–2^ h^–1^ at 5.35 kW m^–2^ solar irradiance	0.81	
membranes	(486)	microporous membrane deposited with Au nanoparticles	11.8 kg m^–2^ h^–1^ under 10 sun irradiation	0.85	
	(295)	W_18_O_49_ mesocrystals on polytetrafluoroethylene membrane	1.13 kg m^–2^ h^–1^ under 1 sun illumination	0.82	
	(263)	black Au membrane	18.5 kg m^–2^ h^–1^ under 20 sun illumination	0.57	
	(487)	PDA/polyethylenimine/PPy@polyamide nanofibrous membrane	1.43 kg m^–2^ h^–1^ under 1 sun illumination	0.869	seawater desalination and wastewater purification
	(488)	WO_2.72_/polylactic acid fiber membrane	3.81 kg m^–2^ h^–1^ at 0.294 W m^–2^ solar irradiance	0.8139	
	(489)	PDA-coated polyvinylidene fluoride membrane	0.49 kg m^–2^ h^–1^ at 0.75 kW m^–2^ solar irradiance	0.45	
	(490)	hybrid membrane of MoS_2_ nanosheets and single-walled nanotubes	6.6 kg m^–2^ h^–1^ at 4 kW m^–2^ solar irradiance	0.915	seawater desalination
	(491)	membrane of PPy-coated stainless steel mesh	0.92 kg m^–2^ h^–1^ under 1 sun illumination	0.58	self-healing hydrophobicity
	(133)	self-assembly of Al nanoparticles in anodic Al_2_O_3_ membrane	5.7 kg m^–2^ h^–1^ under 4 sun illumination	0.9	seawater desalination
	(492)	polyvinylidene fluoride membrane with Al-Ti-O nanostructures	0.50 kg m^–2^ h^–1^ under 1 sun illumination	0.7752	seawater desalination
	(493)	Ag-nanoparticle-loaded polyvinylidene fluoride membrane			temperature polarization
	(494)	Au@TiO_2_ nanoparticles on microporous membrane	3.7 kg m^–2^ h^–1^ under 5 sun illumination	0.4634	seawater desalination and wastewater purification
other composites	(495)	composite of polyethylene glycol and poly(acrylamide-co-acrylic acid) copolymer		0.933	higher thermal conductivity than pure polyethylene glycol
	(496)	composite sheet of HCuPO and polydimethylsiloxane	1.85 kg m^–2^ h^–1^ under 1 sun illumination	0.636	saline water desalination
	(124)	nanocomposite of SiO_2_/Ag@TiO_2_	5.86 L m^–2^ h^–1^ under 7 kW m^–2^ Xe lamp irradiance	N/A	seawater catalysis and desalination
	(497)	3D-cup-shaped composite of CuFeMnO_4_ and silica	2.04 kg m^–2^ h^–1^ under 1 sun illumination	∼1	diffuse reflectance reabsorbed and excess energy gained from the surrounding
	(498)	cermet-coated copper substrate		0.71	thermal concentration and heat localization
	(499)	Janus interface based on copper foil and foam	2.21 kg m^–2^ h^–1^ under 1 sun illumination	0.88	water evaporation and solar-thermal conversion separated on the two sides of the film generator
	(500)	Al-absorber-based solar water purifier	1.063 kg m^–2^ h^–1^ under 1 sun illumination	0.7	minimized optical loss, enhanced heat transfer and condensation
	(265)	assembly of Au nanoparticles in disordered nanoporous Al_2_O_3_ template	5.5 kg m^–2^ h^–1^ under 4 sun illumination	0.9	
	(501)	femtosecond-laser-treated Al foil	1.26 kg m^–2^ h^–1^ under 1 sun illumination	0.427	wastewater purification
	(502)	assembly of Ag nanoparticles in porous Al template	5 kg m^–2^ h^–1^ under 4 sun illumination	0.8	wastewater purification
	(503)	Au-nanoflower-dispersed in nanoporous silica matrix	1.356 kg m^–2^ h^–1^ under 1 sun illumination	0.85	parallel production of fresh water and triboelectricity
	(333)	multilayer PPy nanosheets	1.38 kg m^–2^ h^–1^ under 1 sun illumination	0.92	
	(282)	MoO_3-*x*_ QDs	4.95 kg m^–2^ h^–1^ under 5 sun illumination	0.62	
	(504)	poly(vinyl alcohol) network embedded with V-doped MoO_3_ nanospheres	2.01 kg m^–2^ h^–1^ under simulated solar light	0.9344	strong light absorption through heavy V-doping
	(505)	polymer film embedded with titanium oxynitride spheres	1.49 kg m^–2^ h^–1^ under 1 sun illumination	0.891	seawater desalination
	(506)	multistage solar still	5.78 kg m^–2^ h^–1^ under 1 sun illumination	3.85	vaporization enthalpy recycled

The water evaporation rate and efficiency are key parameters for evaluating the capability of a photothermal system to generate water vapor. The water evaporation rate is defined as the amount of generated vapor by a light absorber directly exposed to solar light of unit area within unit time. The evaporation efficiency η_eva_ is defined as the ratio of the energy consumed for evaporation (*P*_eva_) to the energy of light incident on the photothermal system (*P*_solar_).^507^
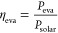
5.1*P*_eva_ can be calculated by the product between the vaporization enthalpy of water and the weight of the evaporated water. The values of the water evaporation rates and efficiencies of various photothermal systems are also listed in Table 1. In addition to increasing the light-absorbing capability of the solar absorber, the water evaporation rate and efficiency can also be improved by optimizing the heat management and water supply, harvesting the environmental energy, as well as reducing and reusing the vaporization enthalpy. Groove structures on the hydrogel surface can introduce temperature gradients and tension gradients, activating the Marangoni convection effect to accelerate water flow (Figure 16c).^465^ The accelerated water flow can reduce the viscosity of the internal water and activate the free water around the surface, increasing the evaporation rate. In contrast to traditional interfacial solar vapor generators with heat loss into the environment, most 3D absorbers can be kept cooler than their surrounding environment, which allows energy to be conveyed from the environment and thus to efficiently diminish the energy loss.^448,467,497^ With the environmental energy used as an additional input, the calculated evaporation efficiencies of those systems can be close to or even beyond 100%.^448,467,497^ A carbon foam with interconnected porous structures has been reported to harvest wind energy as additional energy.^445^ An impressive evaporation rate of 10.9 kg m^–2^ h^–1^ under one sun illumination has been achieved with a convective flow of 6 m s^–1^. Porous networks of polymer films have been reported to confine water into clusters and lead to the reduction of the evaporation enthalpy.^483,504,505^ Reduced vaporization enthalpies have also been observed in hybrid hydrogel and explained by the abundant hydrophilic groups for weakening the interaction of the hydrogen bonds between the water molecules.^465,482^ A thermally localized multistage solar still has been demonstrated to recycle the vaporization enthalpy, where the latent heat released from a stage is recycled at the next stage to produce vapor.^506^ An evaporation rate of 5.78 kg m^–2^ h^–1^ and a remarkable evaporation efficiency of 385% under one sun illumination have been experimentally demonstrated.

#### 5.1.1 Seawater Desalination

**Figure 17:**
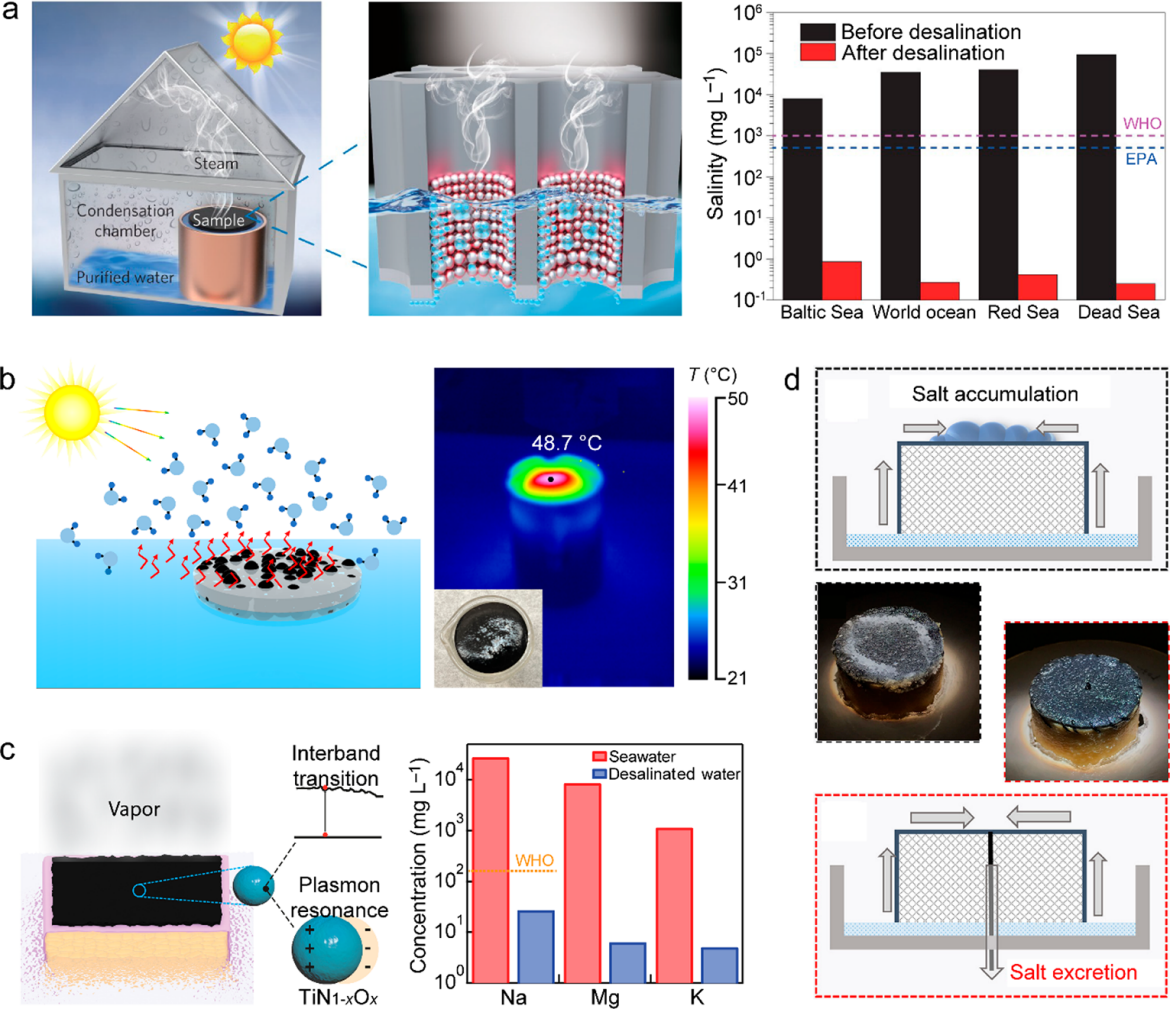
Solar heating for seawater desalination. (a) Setup and solar desalination performance of 3D assembled Al nanoparticles. The measured salinities represent the weight percentages of Na^+^ of the four simulated seawater samples before and after desalination. The dashed colored lines refer to the WHO and the US EPA standards for drinkable water. Reprinted with permission from ref (133). Copyright 2016 Springer Nature. (b) Schematic and IR image of a porous interlaced poly(vinyl alcohol) network embedded with V-doped MoO_3_ nanospheres during solar seawater desalination. Reprinted with permission from ref (504). Copyright 2022 American Chemical Society. (c) Schematic and seawater desalination performance of hollow titanium oxynitride spheres as the photothermal transducers. Reprinted with permission from ref (505). Copyright 2022 American Chemical Society. (d) Illustrations and photographs of salt accumulation/excretion in conventional/salt-excreting evaporators. Reprinted with permission from ref (454). Copyright 2021 Wiley-VCH under the CC BY 4.0 license http://creativecommons.org/licenses/by/4.0/.

Although water covers 71% of the surface of the Earth, most of the water resources are stored in the oceans and are too salty to be directly utilized. Desalination, a process that removes salts from saline water, is regarded as one of the most effective solutions to overcome water scarcity. A few developed countries, such as Saudi Arabia, Singapore, and Israel, have been investing in seawater desalination to address their water scarcity issues. Most current techniques of seawater desalination remain expensive since they require enormous amounts of energy. Solar seawater desalination, taking “free energy” from the Sun and producing negligible hazards to the environment, is a green technology to generate freshwater. The integration of a water condensation system with the solar evaporation system can be directly used for seawater desalination. The vapor generated from seawater under solar illumination by a photothermal system is condensed and collected to produce water with low salinity. In a pioneering work, the nanopores of anodic aluminum oxide membranes act as the paths for efficient water supply and continuous vapor flow (Figure 17a).^133^ Plasmon-enhanced solar desalination has been successfully demonstrated with the broadband absorption of the assembled Al nanoparticles. The salinities of four representative seawater samples after desalination are all remarkably decreased below the salinity levels of drinkable water defined by the World Health Organization (WHO) and the US Environmental Protection Agency (EPA). Solar absorber films have recently been prepared from V-doped MoO_3_ nanospheres. They give a fast seawater evaporation rate of 2.01 kg m^–2^ h^–1^ and a high evaporation efficiency of 93.44% (Figure 17b).^504^ The light absorption of the MoO_3_ nanospheres is significantly enhanced by V-doping, giving rise to over 90% absorption in the wavelength range from 250 to 2000 nm. IR imaging of the solar evaporation system shows that the surface temperature of the solar-absorbing film reaches up to 48.7 °C, while the temperature of the bulk water is barely changed. The synergistic effect between the thermal management and the great light-absorbing capability of the V-doped MoO_3_ nanospheres results in a superior photothermal conversion performance. The interband-transition-induced resonance and plasmon resonance endow nitridized titania nanospheres with a strong light absorption capability (Figure 17c).^505^ The light absorption of the assembly of differently sized titanium oxynitride spheres is significantly enhanced across the solar spectrum. Hollow titanium oxynitride spheres are further incorporated into polyacrylamide-based films and integrated with a 2D water-wicking material for solar seawater desalination. A water evaporation rate of 1.49 kg m^–2^ h^–1^ and an evaporation efficiency of 89.1% have been demonstrated under simulated sunlight. The freshwater generated from the solar seawater desalination system possesses a significantly reduced salinity, satisfying the WHO standards for drinkable water. Moreover, the salts produced during solar evaporation can be automatically cleaned for continuous desalination. Salt management in solar seawater desalination is vital for maintaining the desalination performance with long-term stability (Figure 17d).^454^ During seawater desalination, the leaving of vapor from the bulk water produces concentrated salts, including NaCl and CaCO_3_. The fouling of the slats on an evaporation system can deteriorate the light-absorbing performance and clog the water-supply path. Various designs have been proposed to address the fouling issue. In one design, the source water is taken up from the edge of the evaporator and the concentrated brine is excreted out before saturation through a directional flow.^454^ White fabric wicks in a floating multilayer solar evaporation structure have been used for delivering water and rejecting excess salts at the same time.^479^ A 3D umbrella architecture has been developed to guide the accumulated salts through the predesigned pathways without blocking the evaporative surface.^455^ A stable evaporation rate from a hypersaline brine with a salinity of 20 wt % is achieved over 4 day operation with minimal salt accumulation. In a proposed contactless configuration (Figure 16b), the solar absorber does not touch the water surface and transfers energy to the seawater through thermal radiation, effectively circumventing the fouling problem.^441^

#### 5.1.2 Wastewater Purification

**Figure 18:**
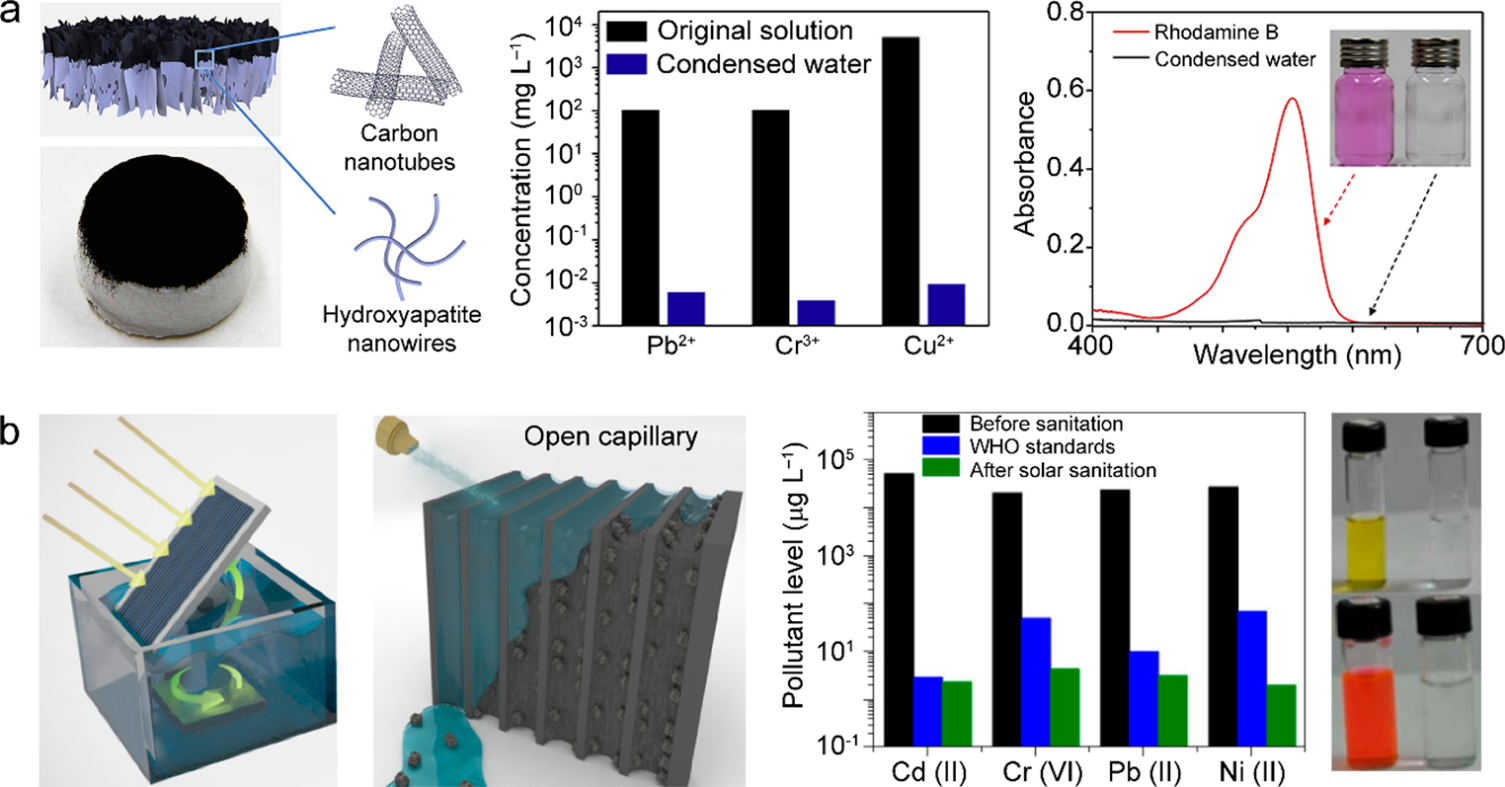
Solar water heating for wastewater purification. (a) Self-floating aerogel composed of CNTs and hydroxyapatite nanowires for highly efficient solar-energy-assisted water purification. The middle panel shows the concentrations of three types of heavy metal ions in the simulated wastewater before and after photothermal wastewater purification. The right panel shows the UV–visible absorption spectra and photographs of a rhodamine B aqueous solution before and after photothermal purification. Reprinted with permission from ref (457). Copyright 2019 Elsevier. (b) A superwicking and super-light-absorbing aluminum surface for efficient solar-based water sanitation. The schematics show the black aluminum sheet with a rotatable platform to maximize the incident solar flux, and an open capillary architecture for easy cleaning. The histogram demonstrates the water sanitation of four types of heavy metals. The right panel shows the photographs of a Cr^2+^ (top) and a rhodamine 6G dye (bottom) aqueous solution before (left) and after (right) photothermal purification. Reprinted with permission from ref (501). Copyright 2020 Singh et al., published under the CC BY 4.0 license http://creativecommons.org/licenses/by/4.0/.

Solar thermal water evaporation can also be employed for wastewater purification. A bilayer aerogel composed of hydrophobic CNTs and hydrophilic hydroxyapatite nanowires has been reported for photothermal water purification (Figure 18a).^457^ The CNT layer exhibits a high solar light absorptivity. The hydroxyapatite nanowires function as a thermal insulator for inhibiting the heat loss to the bulk water and confining the thermal energy at the evaporative surface.^457,458^ The cross-linked pores and channels in the bilayer aerogel promote water transportation and vapor escape. Clean water can be produced from simulated wastewater containing heavy metal ions or organic dyes after vapor generation and condensation. The concentrations of Pb^2+^, Cr^3+^, and Cu^2+^ ions in the purified water are measured to be below the WHO drinking water standards. A superwicking black Al surface has been designed for efficient wastewater purification (Figure 18b).^501^ Femtosecond laser has been used to produce Al sheets with open capillary channels, which can be easily cleaned and reused. The light-absorbing Al sheet is mounted onto polystyrene foam and floated on the water surface to construct a solar evaporation system. The system has been proven to be effective for purifying wastewater with 14 common types of contaminants, including various heavy metals, light metals, as well as industrial, domestic, and agricultural pollutants. After purification, the concentrations of all the tested contaminants are reduced by 4–5 orders of magnitude and meet the WHO standards for drinkable water. Moreover, the device can be mounted onto a rotatable platform to track sunlight for optimizing incident solar irradiance. The versatility of plasmonic structures has been demonstrated in the application of water purification. The pronounced local field enhancement of plasmonic structures in the solar evaporation system enables on-site pollution detection by Raman scattering.^502^ Bifunctional Au@TiO_2_ nanoparticles have been reported for simultaneous vapor generation and photocatalytic degradation of pollutants.^494^ Photothermal-evaporation-induced seawater desalination and wastewater purification are believed to play vital roles in addressing the issues of water scarcity.

#### 5.1.3 Electricity Generation

**Figure 19:**
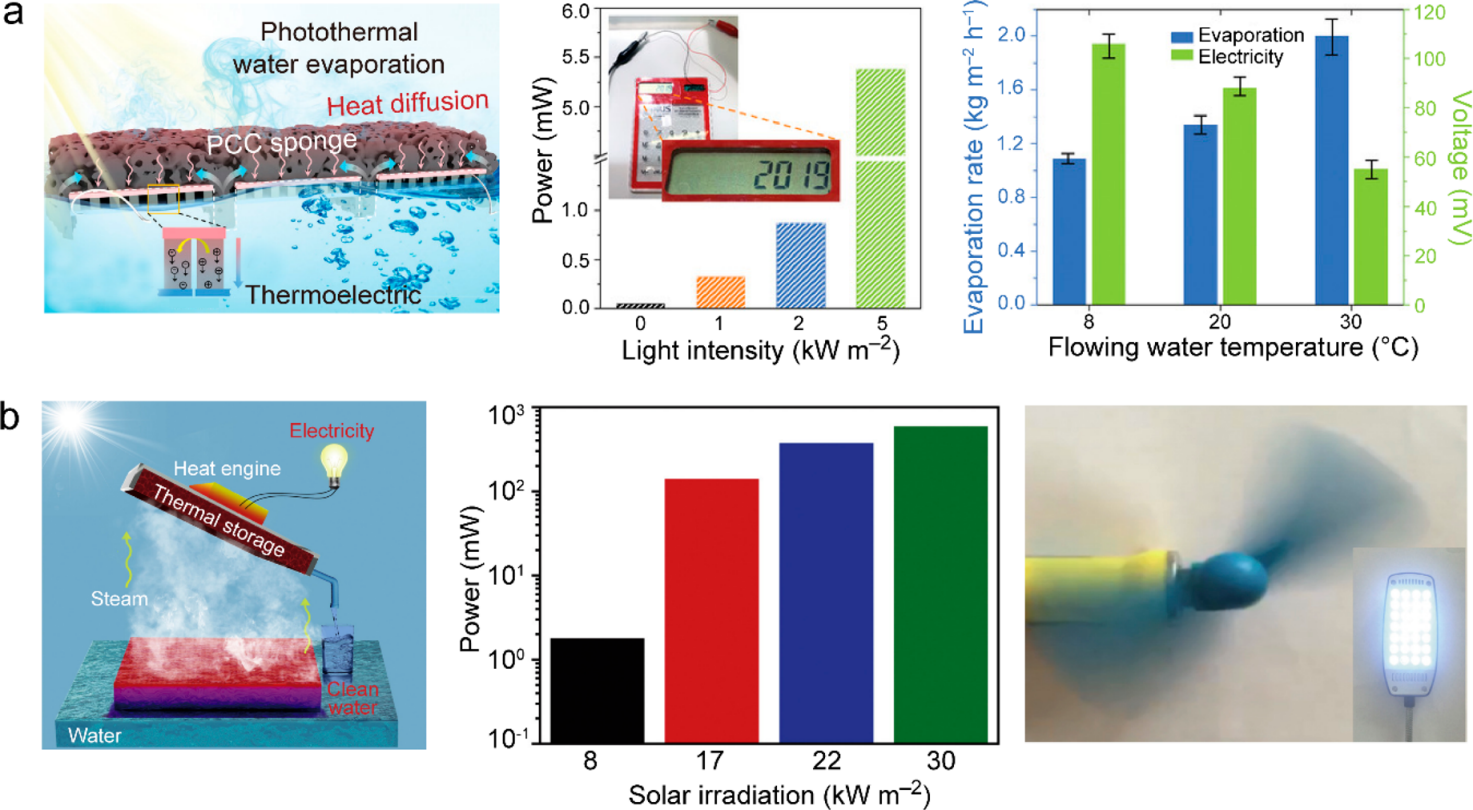
Solar water heating for electricity generation. (a) Schematic of the interfacial photothermal water evaporation and electricity generation processes based on polydimethylsiloxane/carbon nanotubes/cellulose nanocrystals (PCC) sponge and thermoelectric modules (left panel). The middle panel shows the maximum output power of the thermoelectric modules under different solar irradiation powers with a digital calculator powered by the PCC sponge under 5 sun illumination shown in the inset. The right panel displays the water evaporation rates and open circuit voltages of the hybrid photothermal device at different flowing water temperatures under an optical density of 1 kW m^–2^. Reprinted with permission from ref (459). Copyright 2019 Wiley-VCH. (b) Schematic illustrating the condensation process during solar steam generation for the simultaneous generation of clean water and electricity (left panel). The middle panel shows the maximum output power of the thermoelectric device under different solar irradiation powers, and the right panel displays the optical image of an operating electric fan and light-emitting diodes powered by the solar steam system. Reprinted with permission from ref (461). Copyright 2018 under Elsevier user license.

Solar energy is converted into thermal energy and stored in the vapor and heated water after the solar evaporation process. A substantial amount of energy from the input sunlight is wasted if fresh water is the only target of the solar evaporation system.^53^ Making the most use of solar energy along the photothermal evaporation process can contribute in addressing the energy and water scarcities. Researchers have developed strategies to further harvest the input solar energy by generating electricity during the processes of solar absorption, vapor generation, and water condensation. The generation of thermoelectric power at the interface of the photothermal absorbers and the bulk water has been reported (Figure 19a).^459^ A shape-conforming 3D organic sponge is fabricated for solar evaporation and integrated with thermoelectric modules for electricity generation. The sponge absorbs solar light and transfers energy to the upper side of the thermoelectric modules through heat diffusion. With the lower side of the thermoelectric modules cooled by the underneath bulk water, the temperature difference between the two sides of the thermoelectric modules produces electricity by the Seebeck effect. The generated electricity is proportionally boosted with solar irradiation. It can power a digital calculator under 5 sun illumination. On the other hand, the thermoelectric modules also serve as thermal insulators to isolate the sponge from the bulk water, resulting in an improved water evaporation rate. The water evaporation is also improved by the increased temperature of the flowing bulk water. In addition, lowering the temperature of the bulk water also increases the temperature difference between the two sides of the thermoelectric modules, which gives rise to a higher thermoelectric potential. Electricity can also be extracted from the evaporation-induced salinity gradient in a solar desalination system equipped with a piece of ion-selective membrane.^473^ For instance, the storage and recycling of the enthalpy of the solar steam have been demonstrated for the simultaneous generation of electricity and fresh water (Figure 19b).^461^ Heat is transferred from the high-temperature steam to a polyurethane-foam-wrapped Al chamber. Electricity is then generated by Bi_2_Te_3_-based thermoelectric modules due to the temperature difference between the heated chamber and the room-temperature environment. The output electric power reaches 574 mW under 30 kW m^–2^ solar irradiation, enabling continuous operation of an electric fan and 28 light-emitting diodes. Moreover, the chamber with thermal capacity can maintain electricity generation after the light source is turned off. It is beneficial for reducing the influence of intermittent illumination during practical applications. The waste energy from solar vapor can also be harvested by a polyvinylidene fluoride film for electricity generation based on the coupled pyroelectric and piezoelectric effects.^440^ Electricity can also be generated during the condensation and collection of fresh water. For example, the flow of condensed water droplets on a polytetrafluoroethylene surface produces electricity by triboelectric nanogenerators in a condensate collection device.^503^ With further improvement in the energy conversion efficiency and reduction in the device cost, these multifunctional and sustainable systems will move toward commercial applications in the foreseeable future.

### 5.2 Structural Color Printing

**Figure 20:**
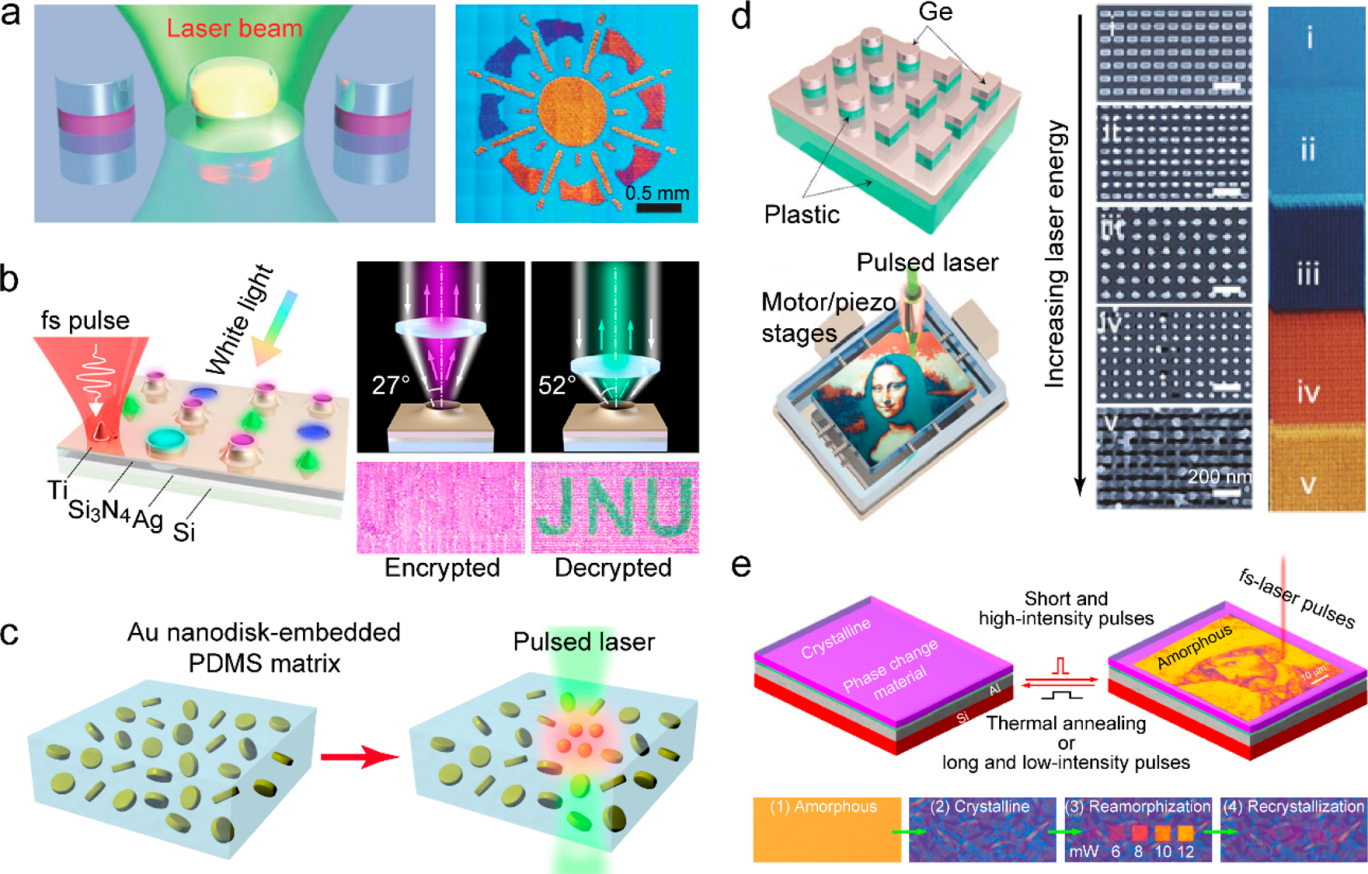
Photothermal color printing. (a) Schematic illustrating laser printing governed by the photothermal reshaping of plasmonic nanostructures (left). A laser-printed plasmonic color image is given on the right as an example. Reprinted with permission from ref (64). Copyright 2016 Springer Nature. (b) Schematic illustrating focused femtosecond-laser-printed nanovolcanoes and scattered colors under oblique illumination (left). The image encryption and decryption under dark-field objective lenses with different incidence angles are shown on the right. Reprinted with permission from ref (515). Copyright 2018 American Chemical Society. (c) Schematics of a Au nanodisk-embedded poly(dimethylsiloxane) (PDMS) matrix before and after laser printing. The Au nanodisks are thermally reshaped into nanospheres under single-pulse laser exposure with a sufficient energy. Reprinted with permission from ref (528). Copyright 2020 Wiley-VCH. (d) Schematics of laser-printing Ge nanostructures for generating structural colors (left). The SEM images of the structures printed by different laser power dosages and the corresponding optical images of the color palettes are shown on the right. Reprinted with permission from ref (65). Copyright 2017 Zhu et al., published under the CC BY 4.0 license http://creativecommons.org/licenses/by/4.0/. (e) Schematic of a rewritable device consisting of a Sb_2_S_3_ phase-change material switched between its crystalline and amorphous states (upper panel). The crystalline sample (purple) is amorphized using femtosecond laser pulses, while the amorphous sample (yellow) is crystallized using a thermal annealing process. The optical micrograph of Vincent van Gogh’s self-portrait can be written on the device by varying the exposure power of the femtosecond laser pulses. On the bottom panel are the optical micrographs of the device with Sb_2_S_3_ in the amorphous (1) and crystalline (2) states. The crystalline sample can be reamorphized to varying degrees by femtosecond laser pulses with different excitation powers (3) and can be switched back to the crystalline state after a thermal annealing process (4). Reprinted with permission from ref (532). Copyright 2020 Liu et al., published under the CC BY 4.0 license http://creativecommons.org/licenses/by/4.0/.

Structural colors arise from the interaction of light with photonic structures at the wavelength or subwavelength scale.^61,508,509^ Fade-resistant and environmentally friendly structural colors with unprecedented printing resolution have gained much attention in numerous applications, including anticounterfeiting labels, optical encryption, high-density optical storage and display. With the development of laser and lithography techniques, direct laser printing becomes a powerful and convenient way for generating structural colors based on the localized photothermal heating of metal and dielectric materials.^85^ The interference between the incident laser and surface EM waves has been used to produce periodic surface structures.^510−514^ Laser illumination can also change the morphology or phase of prefabricated micro-/nanostructures, giving rise to target colors. Laser pulses have been used to generate transient local heat for the melting and reshaping of nanoimprinted Al nanodisks (Figure 20a).^64^ Laser pulses with different energy densities produce Al nanostructures with various morphologies, which support different plasmonic resonances to create colorful appearances. Color images are printed with a speed of one nanosecond per pixel and a resolution of up to 127,000 dots per inch, exceeding the diffraction limit. In another work, plasmonic nanovolcanoes at different splashing stages are produced on a Ti thin film by laser pulses with varied energy fluences (Figure 20b).^515^ The scattering colors of the nanovolcanoes are found to be dependent not only on the morphologies but also on the incidence angle of the illumination light. The angularly anisotropic color appearances of the plasmonic nanovolcanoes are therefore utilized to encrypt hidden color images for information security and anticounterfeiting. Interestingly, the photothermal reshaping of plasmonic nanostructures by laser pulses can also be realized below the melting point of a bulk metal, as explained by a surface-diffusion model.^382,516,517^ Besides the laser power, adjusting the laser wavelength and polarization also provides degrees of freedom to control the structural morphologies and resultant color pixels.^518−520^ Two orthogonal arms of anisotropic plasmonic nanostructures can be separately reshaped by lasers with their polarization along different directions, achieving multiplexed pixels.^519^ Instead of writing on the 2D surfaces of different materials,^521−525^ the structural color prints can alternatively be prepared inside 3D matrices.^526−528^ The laser printing of plasmonic colors inside transparent Au-nanodisk-embedded polymeric matrices has been demonstrated (Figure 20c).^528^ The position of the focused laser spot can access a 3D space in different depths inside the polymer with uniformly distributed circular Au nanodisks, creating multiple layers of color patterns inside a single piece of matrix. The employment of more degrees of freedom endows structural color prints with high security levels and large storage densities.

Dielectric materials, as an alternative to metals for certain optical applications, can also be photothermally modified by direct laser writing.^65,529−532^ For example, Ge nanostructures can harvest energy from pulsed laser irradiation through above-bandgap absorption and undergo morphology changes (Figure 20d).^65^ In contrast to plasmonic heating with a rapid thermal change at the metal surface, the evenly distributed electric field inside the dielectric resonators potentially heats the resonators more homogeneously, making the associated photothermally driven morphology changes more controllable. With the increase of the laser power, Ge nanorods are gradually changed to shortened rods, spheres, and finally holes, causing the evident color variations. Furthermore, using phase-change materials, one can realize rewritable structural color prints, as have been reported in Sb_2_S_3_ films (Figure 20e).^532^ Sb_2_S_3_ is a phase-change material that possesses a refractive index difference as large as ∼1 between its crystalline and amorphous states.^532,533^ The crystallization of Sb_2_S_3_ films is achieved by thermal annealing, while the crystalline-to-amorphous transition is realized by employing high-intensity laser pulses with short duration to randomize the atomic arrangement. Partial reamorphization states with intermediate colors have also been demonstrated by changing the excitation power of the laser pulses. With further improvement in the write–erase cyclability of the color changes, such phase-change materials can become promising for optical encryption and next-generation high-resolution color display devices.

### 5.3 Photothermal Manipulation

#### 5.3.1 Opto-thermophoresis of Nanomotors

**Figure 21:**
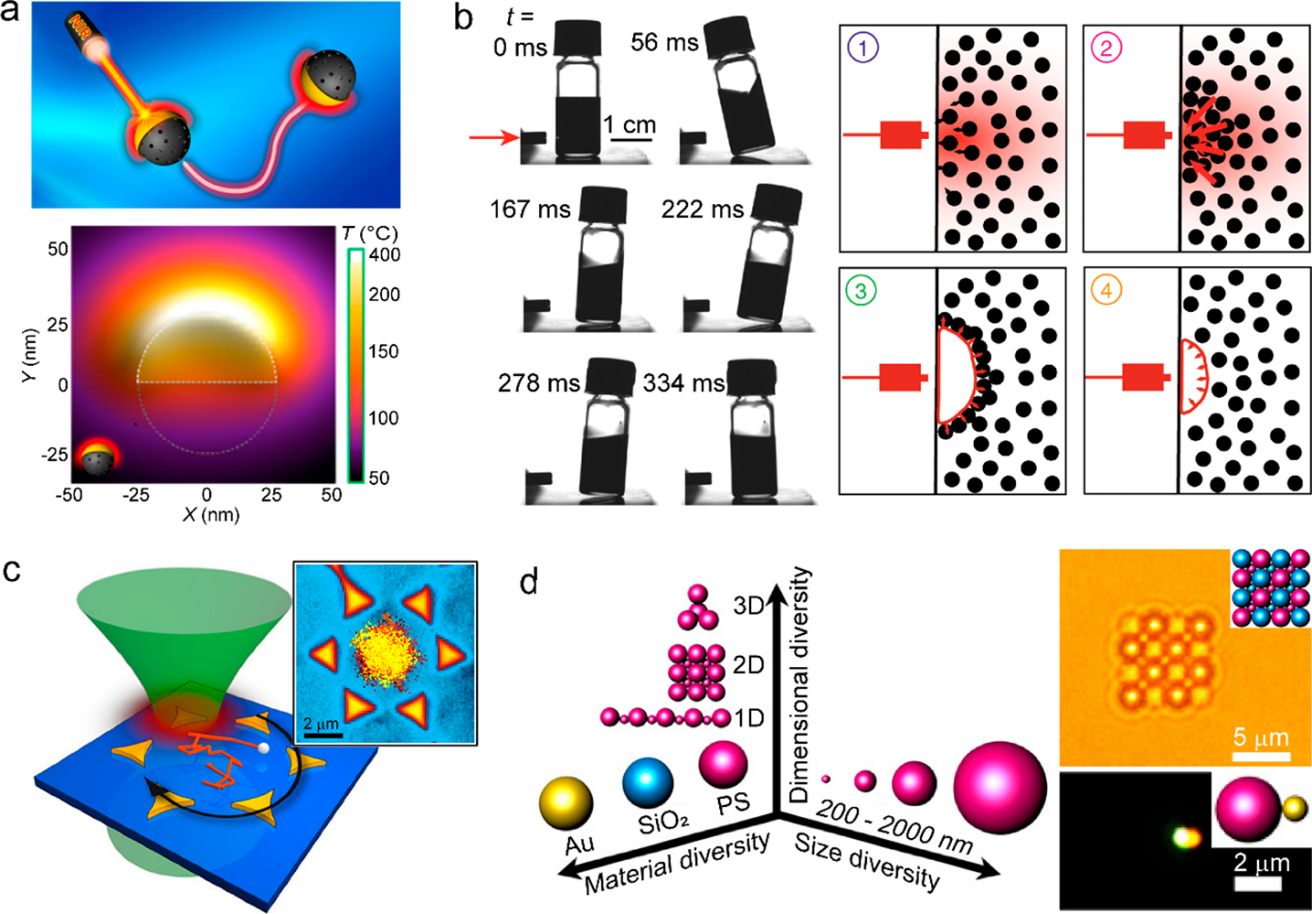
Opto-thermophoresis of nanomotors. (a) Schematic (upper) of a NIR-light-powered Janus mSiO_2_ nanomotor and calculated steady-state temperature distribution (lower) of a representative nanomotor under 3 W cm^–2^ irradiation. Reprinted with permission from ref (539). Copyright 2016 American Chemical Society. (b) Snapshots (left) and schematics (right) showing the mechanism of the motion of a PbS-nanoparticle-filled vial propelled by a NIR laser (indicated by a red arrow). The proposed mechanism of the photomechanical effect contains four steps: (1) thermophoretic motion of the PbS nanoparticles toward the laser source; (2) brutal force release induced by Jeans’ instability; (3) explosive growth of a bubble, which disperses the accumulated PbS nanoparticles; and (4) collapse of the bubble and return to the initial state. The temperature gradient is represented by the red color. Reprinted with permission from ref (561). Copyright 2020 Kavokine et al., published under the CC BY 4.0 license http://creativecommons.org/licenses/by/4.0/. (c) Schematic showing the trapping of a single polystyrene nanosphere in an open gold structure on a glass substrate. The inset shows the trajectory points of a 200 nm polystyrene sphere trapped within a hexagonal lattice of triangular Au pads by a moving laser with a rotation frequency of 18.9 Hz. Reprinted with permission from ref (573). Copyright 2013 American Chemical Society. (d) Schematic and two representative examples of the opto-thermophoretic assembly of colloidal matter of diverse sizes (from the subwavelength to micrometer scale) and materials (polymeric, dielectric, metal) with versatile configurations. A bright-field optical image of a 2D hybrid superlattice of 2 mm polystyrene spheres, 0.96 mm polystyrene spheres, 2 mm silica beads, and 1 mm silica beads and a dark-field optical image of a heterogeneous dimer of a 500 nm polystyrene sphere bead and a 200 nm Au nanosphere are shown on the right panel. Reprinted with permission from ref (575). Copyright 2017 Lin et al., published under the CC BY 4.0 license http://creativecommons.org/licenses/by/4.0/.

Motors, which can convert other forms of energy into mechanical work to produce motion, play a crucial role in the development of human society.^57,534^ The miniaturization of motors toward the nanoscale has attracted intensive research interests.^58^ The opto-thermophoretic effect provides a low-power solution for manipulating nanomotors by creating a temperature gradient field through optical heating.^535,536^ The hydrostatic pressure induced by a temperature gradient field occurs in the vicinity of the nanomotor surface. It moves the nanomotor to decrease the interfacial free energy.^55^ In general, nanomotors with positive (negative) Soret coefficients are thermophobic (thermophilic) and move from the hot (cold) region to the cold (hot) region. The magnitude and sign of the Soret coefficient are affected by many parameters, including the shape and size of the nanomotor, the nanomotor–solvent interfacial properties, the surrounding temperature, and the hydrodynamic boundary effects.^55,536−538^ In a work with Janus mSiO_2_ nanomotors fabricated by sputtering a 10 nm Au layer on one side of mSiO_2_ nanoparticles (Figure 21a),^539^ a NIR laser is employed to locally heat the Au half-shell, resulting in the formation of a thermal gradient across the nanomotor. The opto-thermophoretic effect actively drives the nanomotor to move opposite to the direction of the Au half-shell side at a speed up to 950 body lengths per second under a laser power of 703 kW m^–2^. Different types of opto-thermophoretic nanomotors have been reported, including plasmonic nanostructures,^540−543^ carbonaceous nanoparticles,^544−546^ biomolecules,^547,548^ and hybrid nanoparticles.^549−560^ Opto-thermophoretic manipulation can be achieved at not only the nanoscale but also the macroscopic scale. The displacement of a vial of nanofluids over centimeter-scale distances has been realized by a laser beam irradiation (Figure 21b).^561^ The laser-irradiated PbS nanoparticles in the vial absorb the incident light and serve as local heating sources to trap other nanoparticles in the heated region through negative thermophoresis. Once the particle density in the illuminated region increases up to a point beyond the Jeans’ instability, the collective motion of the PbS nanoparticles occurs and pushes the entire vial away from the laser. Remarkably, a 1.5 W laser illumination is sufficient to propel a vial weighing 3.5 g at an average speed of 1 mm s^–1^. The opto-thermophoretic effect has also been used for trapping^562−570^ and assembling^571,572^ nanoparticles. An opto-thermophoretic trapping platform has been built for thermophobic nanoparticles (Figure 21c).^573,574^ Since polystyrene nanospheres are repelled away from the laser-heated Au pads, the thermophobic nanoparticles can be confined inside a hexagonal lattice of the Au pads by rotating the laser at suitable speeds. A strategy of opto-thermophoretic assembly has been developed to manipulate and construct colloidal matter based on ionic depletants under a light-controlled temperature field (Figure 21d).^575^ Diverse colloidal particles, as the building blocks, can be trapped and moved one by one to assemble into various superstructures of arbitrary 1D, 2D, and 3D configurations. Moreover, the superstructures can be disassembled and then reassembled into different configurations.^576,577^

#### 5.3.2 Photothermal Actuators and Robots

**Figure 22:**
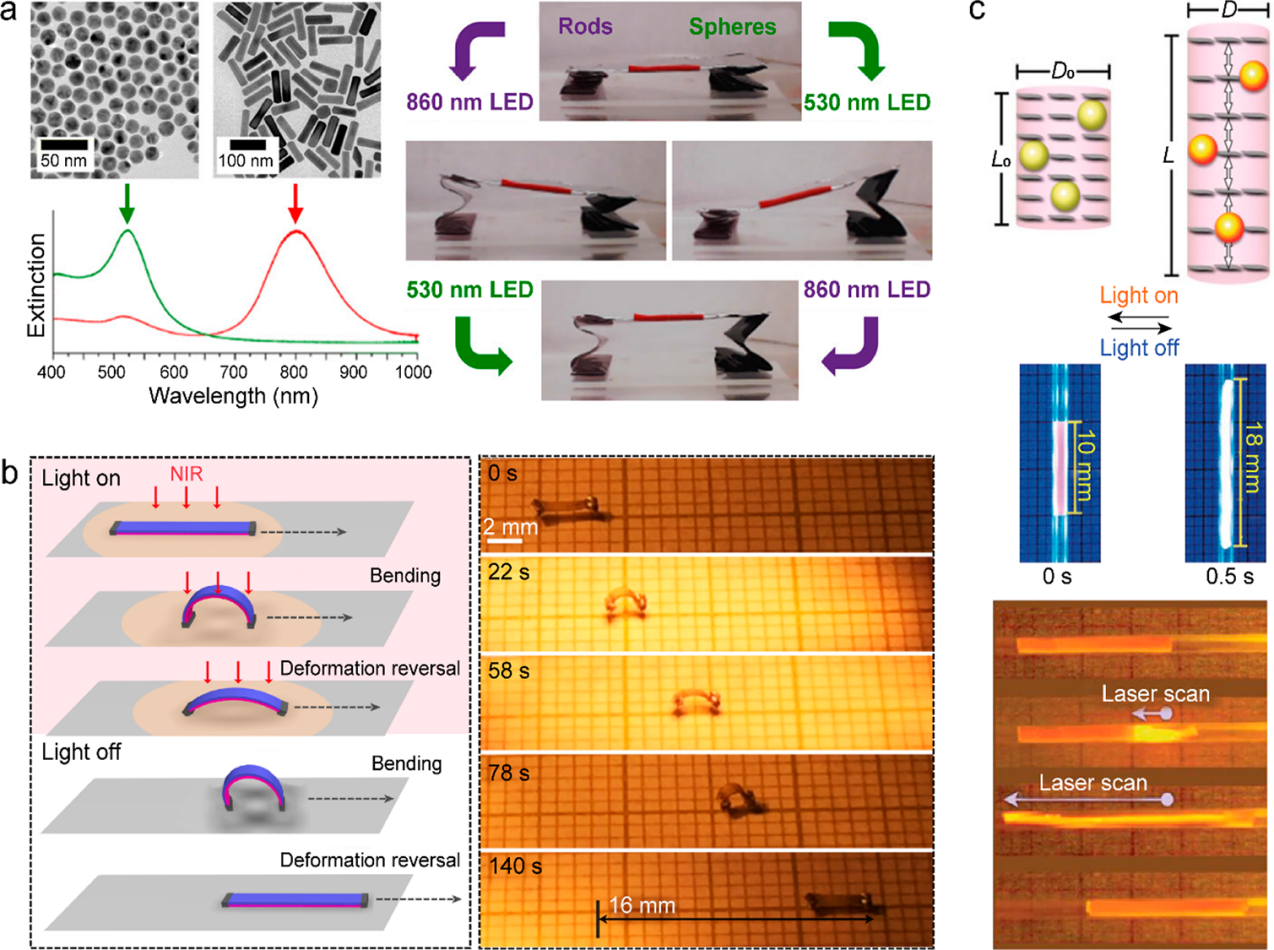
Photothermal actuators and robots. (a) Sequential actuation of shape-memory polymer films through the wavelength-selective photothermal heating of Au nanospheres and nanorods. The TEM images and extinction spectra of the Au nanospheres and nanorods are shown on the left panel. A wavelength-controlled stage is demonstrated on the right panel with optically controlled heights and tilt angles using two legs made of folded shape-memory polymer films embedded with the Au nanospheres and nanorods, respectively. Two light-emitting diodes are used to selectively drive the shape recovery of the two legs. Reprinted with permission from ref (578). Copyright 2018 American Chemical Society. (b) Schematics and photographs of PDA-coated LCN actuators, which can advance two steps through arching up/flattening down in a single on and off irradiation cycle. Reprinted with permission from ref (585). Copyright 2021 Xiao et al., published under the CC BY 4.0 license http://creativecommons.org/licenses/by/4.0/. (c) Earthworm-like directed peristaltic crawling enabled by an anisotropic hydrogel actuator. The upper and middle panels show the schematics and photographs of the light-responsive actuation of a hydrogel consisting of titanate nanosheets aligned orthogonally to the cylindrical gel axis and light-absorbing Au nanoparticles. The hydrogel undergoes peristaltic crawling and moves oppositely toward the laser scanning direction when the irradiation spot is moved along the cylindrical gel axis, as shown in the photographs (bottom panel). Reprinted with permission from ref (598). Copyright 2018 Wiley-VCH.

Plasmonic nanoparticles, carbon nanomaterials, and organic photothermal agents have been incorporated into the matrices of shape-memory polymers,^578−584^ liquid crystals,^585−597^ hydrogels,^136,598−604^ elastomers,^605,606^ and biopolymers^607,608^ to form photothermal actuators and robots. The photothermal conversion by light-absorbing materials leads to basic mechanical deformations, such as bending, twisting, rotating, and jumping, of thermally responsive components to function as grippers, mills, swimmers, and syringes.^66,68,609−612^ Light-absorbing materials also serve as thermally responsive components in some actuators.^613−615^ Several metrics, including the generated stress and strain, Young’s modulus, durability, response time, and energy consumption, have been taken into account during the design of photothermal actuators and robots.^616−618^ Two types of Au nanocrystals, nanospheres and nanorods have been selectively dispersed into thermoplastic polyurethane shape-memory polymer films with a transition temperature of 55 °C (Figure 22a).^578^ The sizes of the Au nanospheres and nanorods are carefully adjusted so that their LSPR wavelengths are matched with those of two light-emitting diodes at 530 and 860 nm, respectively. When the Au nanocrystal-embedded polyurethane films are exposed to the 530 (860) nm light, only the polyurethane film embedded with the wavelength-matched Au nanocrystals (nanospheres or nanorods) undergoes a shape deformation. The wavelength-selective photothermal heating therefore enables the sequential actuation of a stage with the folded shape-memory polymer films. Desynchronized liquid crystalline network (LCN) actuators have been designed with a deformation reversal capability (Figure 22b).^585^ The two sides of the LCN actuators are made to start deforming at different temperatures and exerting different reversible strains by asymmetrical cross-linking and/or asymmetrical stretching. PDA layers are coated on the cross-linked LCNs to serve as the photothermal agent. After being exposed to a light source with a power density of 30.5 mW mm^–2^, the PDA-coated LCN film arches up and then flattens down, taking a step forward. After the light is switched off, the film bends again and then flattens down to the initial state as it further cools, moving another step forward. The LCN film therefore implements two steps forward in a single light-on/off cycle. In another example, an anisotropic hydrogel has been created. It exhibits the capability of an earthworm-like directed peristaltic crawling inside a glass capillary (Figure 22c).^598^ The hydrogel contains Au nanoparticles for photothermal conversion, a thermoresponsive polymer network for switching the electrical permittivity of the hydrogel interior, and magnetically oriented titanate nanosheets to synchronously change their anisotropic electrostatic repulsion. The employed poly(*N*-isopropyl acrylamide) hydrogel can reversibly dehydrate and rehydrate upon heating and cooling, causing reversible expansion and contraction of the hydrogel along the direction orthogonal to the plane of titanate nanosheets. Once the hydrogel is illuminated with a visible laser, the irradiated region becomes thinner and longer, resulting in the reduction of friction with the capillary glass. When the irradiation spot is scanned along the cylindrical axis, the hydrogel undergoes peristaltic crawling and moves oppositely toward the laser scanning direction owing to quick and sequential elongation/contraction events. Besides the wavelength and intensity of the incident light,^578,619−622^ the photothermal actuators and robots can be further controlled by the polarization of the light sources.^623,624^ Noncontact manipulation of photothermal actuators and robots with improved response speeds, energy conversion efficiencies, and mechanical robustness will pave a promising way toward smart autonomous systems.

### 5.4 Photothermal Catalysis

**Figure 23:**
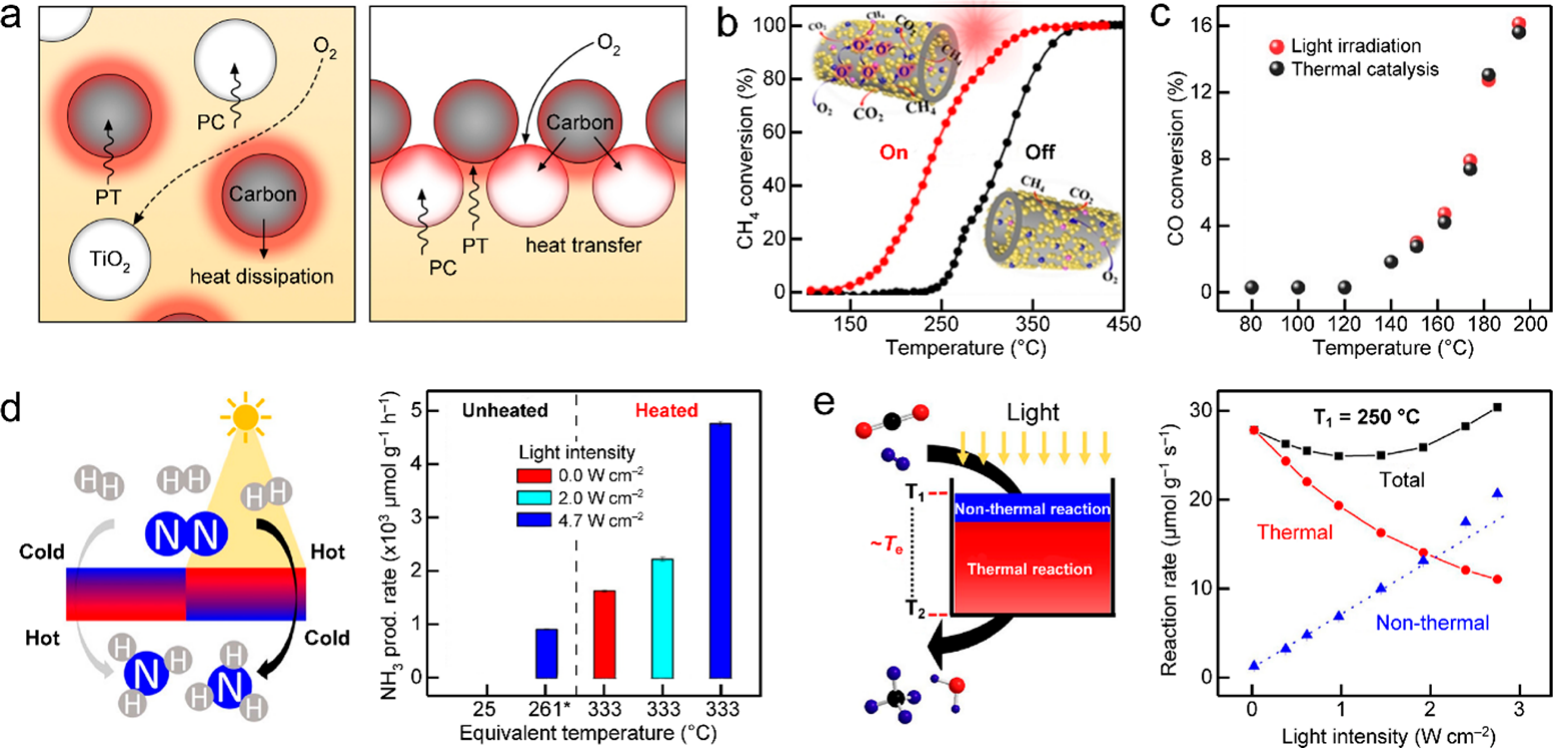
Three categories of photothermal catalysis. (a) Thermally assisted photocatalysis. Schematic illustrating the photothermal effect and oxygen diffusion in a diphase (left) and a triphase (right) system. PC represents photocatalysis and PT represents the photothermal effect. Reprinted with permission from ref (276). Copyright 2021 Wiley-VCH. (b,c) Photoassisted thermocatalysis. The CH_4_ conversion with PdO/Mn_3_O_4_/CeO_2_ nanocomposite supported on 1D halloysite nanotubes with and without light irradiation (on and off) is shown in (b). Reprinted with permission from ref (635). Copyright 2021 Wiley-VCH. The CO conversion with a Co-based catalyst with and without UV–visible irradiation (photothermal heating/direct thermal heating) is shown in (c). Reprinted with permission from ref (636). Copyright 2018 Wiley-VCH. (d,e) Photothermal cocatalysis. The schematic (left) illustrating the effect of light-induced thermal gradients on ammonia production and the NH_3_ production rates (right) under dark and different illumination conditions are shown in (d). Reprinted with permission from ref (637). Copyright 2019 American Chemical Society. The schematic (left) showing the modified reaction chamber for the temperature measurements of thermal and nonthermal reactions and the total, thermal, and nonthermal reaction rates (right) as functions of the light intensity are shown in (e). Reprinted with permission from ref (639). Copyright 2018 American Chemical Society.

Heat and light are two crucial physical quantities that drive chemical reactions. With the rapid development of photothermal nanomaterials, photothermal catalysis has been widely explored to enhance the catalytic activity and achieve high selectivity for specific products, even under moderate reaction conditions.^9,45,625^ As light and thermal energy can collectively or separately participate in the catalytic process, photothermal catalysis can be divided into three major categories.^8,626^ The first category is thermally assisted photocatalysis, where photons mainly drive the catalytic reaction with thermal energy acting as an assisting role. Thermal energy alone can hardly drive this type of catalytic reactions. The photothermal effect is mainly reflected in the rising temperature that accelerates the migration of photogenerated charge carriers and the diffusion of reactant molecules.^627−629^ The assisting thermal energy can apparently reduce the activation energy and thus facilitate the catalytic process. To better utilize the photothermal effect in photocatalytic reactions, the gas–liquid–solid triphase system has been proposed in several recent works for improving the catalytic performance.^630−633^ In conventional liquid–solid diphase systems, the thermal energy is rapidly dissipated to the surrounding medium and less can contribute to the interfacial reaction (Figure 23a).^276^ On the contrary, the generated thermal energy that concentrates at the local sites of a carbon layer can be readily transferred to the adjacent catalysts in the triphase system. With the efficient heat supply, the triphase photocatalytic reaction rate can therefore be improved by 13 times in comparison to that in the diphase system. However, it is worth mentioning that more thermal energy does not always bring better catalytic performances. The balance between thermodynamics and kinetics is normally manipulated to achieve the best catalytic performance.^626^ Moreover, high temperatures also challenge the stability of catalysts, which significantly affects the catalytic performance.

The second type of photothermal catalysis is photoassisted thermal catalytic activity, where the thermochemical reaction is dominant while photogenerated charge carriers serve as promoters. The introduction of light can lead to the increase in the local temperature because of the direct vibration absorption and indirect nonradiative relaxation.^94,634^ These two impacts are thus beneficial for promoting the photoassisted thermal catalytic process. For example, the activity of a catalytic reaction can be boosted by the enhanced capability of activating and adsorbing oxygen with the help of photogenerated electrons (Figure 23b).^635^ In the extreme case, light can only be used to generate heat. The catalytic performance under light irradiation shows a very similar tendency to the case of traditional thermal catalysis (Figure 23c).^636^ This type of photocatalysts is desired to have narrow bandgaps and high photothermal conversion efficiencies.

The third category is photothermal cocatalysis, where the thermochemical and photochemical processes make a synergistic contribution to the catalytic reaction. The cocatalytic performance is better than the overall contribution of individual photo- and thermocatalytic reactions. The thermal gradients modulated by photothermal heating have been demonstrated to efficiently produce ammonia with high conversion yields and reaction rates (Figure 23d).^637^ The utilization of plasmonic Ru nanoparticles can facilitate the photothermal effect, but its limit hot carrier generation owing to the broad and weak plasmon resonance.^638^ As shown in Figure 23d, simultaneously introducing light and external heating into the catalytic system can largely enhance the production of NH_3_. The NH_3_ production rate is even larger than the total rate of those under sole light illumination and sole external heating, indicating a synergistic contribution of the thermochemical and photochemical processes in the reaction. The nonthermal plasmonic effect such as hot charge carrier transfer is ruled out from the contribution to the enhancement of the NH_3_ production rate by comparing the catalytic performance under direct and indirect illumination. To further distinguish and quantify the thermal and nonthermal contributions in such a photothermal cocatalytic system, the research group has developed a methodology to *in situ* measure the temperatures in the thermal and nonthermal reactions (Figure 23e).^639^ The effective thermal and nonthermal reaction rates can be correctly extracted by a thermal gradient model based on the measured temperatures.

In the catalytic systems involving plasmonic nanomaterials, photothermal heating and nonthermalized (hot) charge carriers can be effectively generated due to the well-known LSPR effect.^266^ Both of them can contribute to the enhancement of the catalytic activities, even though these two phenomena happen at different time scales and stem from different physical processes.^86^ Therefore, much effort has been made to distinguish the photothermal effect from the hot charge carrier effect in photothermal catalysis.^640−644^ But it has still remained challenging to unambiguously identify the contributions of these two factors due to the involved wide time scales and diverse mechanisms.

### 5.5 Selected Applications in Life Sciences

#### 5.5.1 Photothermal Therapy (PTT)

With the fascinating development of photothermal materials, PTT has emerged as a powerful potential therapeutic technology because of its minimal invasiveness, low toxicity, and spatiotemporal selectivity.^645,646^ PTT makes use of photothermal nanomaterials to produce topical hyperthermia to trigger the death of abnormal cells.^10^ When a photothermal agent is activated by external laser irradiation at a specific wavelength, the generated heat can accumulate in lesions and precisely target at tumors. Relatively low input power is required for tumor ablation because of the efficient light absorption of photothermal nanomaterials. Such selective and effective thermal ablation can minimize the damage to the surrounding healthy tissues. To achieve complete eradication of tumors, NIR light with long wavelengths, especially in the NIR-II window, is preferable for the deep tissue penetration during PTT.^11,647,648^ Moreover, photothermal nanomaterials with desired absorption wavelengths can be readily designed to avoid the intrinsic light absorption of biological chromophores, leading to the reduction of phototoxicity in normal tissues. The modulation of the size, shape, and surface functionalization of photothermal nanomaterials, as well as the biological microenvironment, can be performed to improve the delivery efficiency and therapeutic efficacy of photothermal agents.^71,649−652^ Robust photothermal nanomaterials have been extensively developed to possess specific physicochemical and pharmaceutical properties for the reduction of adverse effects and the enhancement of therapeutic efficacy.^20,24,46,653,654^ In addition, PTT has also been carried out in clinical pilot trials as a safe and effective therapeutic modality. The clinical progresses of PTT with various photothermal nanomaterials have been widely made for the treatment of head and neck cancer, coronary atherosclerosis, diabetic macular abnormalities, age-related macular degeneration, prostate cancer, and genitourinary syndrome of menopause (national clinical trials: NCT 00848042, NCT 01270139, NCT 01975103, NCT 02569892, NCT 02680535, NCT 03288883).^10,11^ A single-treatment clinical device study of AuroLase Therapy for the direct focal ablation of prostate tissue was demonstrated by the company of Nanospectra Biosciences.^655^ Since silica-cored Au nanoshells served as the first photothermal nanomaterials for clinical use, more and more inorganic agents with strong absorption in the NIR region, high chemical stability, adjustable water solubility, and minimal cytotoxicity have entered clinical trials.^648,653^ Clinical trials have also been underway for small organic molecules due to their good biodegradability, good repeatability, and simple preparation.^690,713^ Nevertheless, the major challenges for the clinical implementation of photothermal nanomaterials are the complex metabolism and excretion behaviors.^719^ The representative works on PTT with diverse photothermal nanomaterials in recent years^656−706^ are summarized in Table 3.

**Table 3:** Representative Works on PTT in Recent Years

category	reference	photothermal materials and devices	illumination conditions	photothermal conversion efficiency
metals	(656)	Au nanostars	808 nm laser irradiation (0.5 W cm^–2^, 5 min)	85.5%
	(657)	Au nanoparticles	808 nm laser irradiation (1 W cm^–2^, 5 min)	53.6%
	(658)	Au-Pd nanoparticles	808 nm laser irradiation (0.5 W cm^–2^, 5 min)	41.9%
	(659)	(Ag nanocube core)@(Au nanorod shell) nanostructures	808 nm laser irradiation (1 W cm^–2^, 10 min)	87.28%
	(660)	Au nanostars	785 nm laser irradiation (0.02 W cm^–2^, 20 min)	63.6%
	(661)	Au nanoparticles	808 nm laser irradiation (1 W cm^–2^, 10 min)	48.4%
	(662)	Pd nanosheets	808 nm laser irradiation (1.5 W cm^–2^, 2 min)	39.2%
	(663)	Cu nanoparticles	808 nm laser irradiation (0.3 W cm^–2^, 10 min)	26.5%
	(664)	Au nanostars	1064 nm laser irradiation (0.5 W cm^–2^, 5 min)	67.1%
	(665)	Mn nanoparticles	1064 nm laser irradiation (1.2 W cm^–2^, 10 min)	22.1%
	(666)	Fe nanoparticles	808 nm laser irradiation (0.95 W cm^–2^, 20 min)	67%
	(667)	Ag nanoparticles	808 nm laser irradiation (0.25 W cm^–2^, 5 min)	71.68%
	(668)	Mn nanoparticles	940 nm laser irradiation (1 W cm^–2^, 7 min)	70%
	(669)	Cu single atoms	1064 nm laser irradiation (0.39 W cm^–2^, 5 min)	41.6%
	(670)	Au nanorods	980 nm laser irradiation (0.1 W cm^–2^, 5 min)	40.62%
semiconductors	(671)	MnO_*x*_/PDA nanobombs	808 nm laser irradiation (2 W cm^–2^, 5 min)	34.8%
	(672)	flower-like MnO_2_ nanoparticles	808 nm laser irradiation (1 W cm^–2^, 15 min)	21.3%
	(673)	γ-Fe_2_O_3_ nanoparticles	1064 nm laser irradiation (1.1 W cm^–2^, 10 min)	59.85%
	(674)	Fe_3_O_4_@Cu_1.77_Se nanoparticles	1064 nm laser irradiation (0.75 W cm^–2^, 10 min)	67.6%
	(675)	Fe_3_O_4_ nanoparticles	808 nm laser irradiation (1 W cm^–2^, 10 min) or 650 nm laser irradiation (0.5 W cm^–2^, 10 min)	34.6%/17.9%
	(676)	Sb_2_O_3_ nanoparticles	1210 nm laser irradiation (1 W cm^–2^, 8 min)	44%
	(677)	FeS nanoparticles	1064 nm laser irradiation (1 W cm^–2^, 6 min)	56.51%
	(678)	Fe_3_O_4_ nanoparticles on graphene oxide nanosheets	808 nm laser irradiation (1.5 W cm^–2^, 10 min)	55.89%
	(679)	CuS nanoparticles	1060 nm laser irradiation (1 W cm^–2^, 10 min)	57.9%
	(680)	CuS nanoparticles	808 nm laser irradiation (0.5 W cm^–2^, 5 min)	45.6%
	(681)	IrWO_*x*_ nanoparticles	808 nm laser irradiation (1 W cm^–2^, 5 min)	27%
	(682)	CuSe nanoparticles	808 nm laser irradiation (3 W cm^–2^, 10 min)	31.9%
organic polymers	(683)	spirolactone nanoparticles	808 nm laser irradiation (1.5 W cm^–2^, 10 min)	36.9%
	(684)	two isoindigo-based semiconducting conjugated polymers	808 nm laser irradiation (0.8 W cm^–2^, 6 min)	70.6%
	(685)	PTTe nanoparticles	1064 nm laser irradiation (1 W cm^–2^, 10 min)	47.5%
	(686)	zwitterion–liposome hybrid nanoparticles	1064 nm laser irradiation (1 W cm^–2^, 6 min)	30.8%
	(687)	O-T molecular oligomerization nanostructures	1064 nm laser irradiation (1.0 W cm^–2^, 5 min)	73%
	(688)	DTTVBI nanoparticles	808 nm laser irradiation (0.8 W cm^–2^, 6 min)	45.8%
	(689)	N^+^TT-mCB nanoparticles	808 nm laser irradiation (0.45 W cm^–2^, 5 min)	78%
	(690)	BNDI-Me π-conjugated molecules	808 nm laser irradiation (0.3 W cm^–2^, 10 min)	50%
	(691)	nanoparticles of A-D-A type planar phototheranostic agent	808 nm laser irradiation (0.3 W cm^–2^, 10 min)	80%
	(692)	J-aggregates of aza-coating heptamethine cyanines	808 nm laser irradiation (1 W cm^–2^, 5 min)	57.59%
	(693)	nanoparticles of D-A-D type conjugated small molecules	1064 nm laser irradiation (1 W cm^–2^, 8 min)	35.8%
	(694)	D-A conjugated Ru(II)-arene complex nanoparticles	808 nm laser irradiation (0.5 W cm^–2^, 10 min)	24.2%
	(695)	V-shaped DUT850 molecules	808 nm laser irradiation (0.33 W cm^–2^, 5 min)	60%
	(696)	nanoparticles derived from borane-modified dianthracenylpyrazines	808 nm laser irradiation (1 W cm^–2^, 5 min)	41.8%
	(697)	nanoparticles of organic metal adjuvants	1064 nm laser irradiation (1 W cm^–2^, 5 min)	47.0%
	(698)	supramolecular photothermal nanodrugs	680 nm laser irradiation (0.5 W cm^–2^, 10 min)	48.0%
	(699)	glucose oxidase engineered PAn nanoplatforms	808 nm laser irradiation (1 W cm^–2^, 10 min)	55%
	(700)	nanoparticulate heavy-atom-free boron dipyrromethene dye derivative	808 nm laser irradiation (2 W cm^–2^, 5 min)	60.02%
	(701)	nanoaggregates of polymeric photothermal agents coated with DC membranes	808 nm laser irradiation (1 W cm^–2^, 5 min)	30.5%
	(702)	aza-boron dipyrromethene dimers	915 nm laser irradiation (0.54 W cm^–2^, 10 min)	60.3%
	(703)	polymer multicellular nanoengagers	1064 nm laser irradiation (1 W cm^–2^, 6 min)	88.8%
2D nanomaterials	(704)	sheet-like 2D manganese(IV) complex	730 nm laser irradiation (0.75 W cm^–2^, 5 min)	71%
	(705)	Sn nanosheets	808 nm laser irradiation (1 W cm^–2^, 10 min)	37.9%
	(706)	Fe-based 2D nanosheets	1064 nm laser irradiation (1 W cm^–2^, 10 min)	43.3%
carbon-based materials	(313)	SWCNTs and MWCNTs	808 nm laser irradiation (1 W cm^–2^, 5 min)	59.3%/57.8%

**Figure 24:**
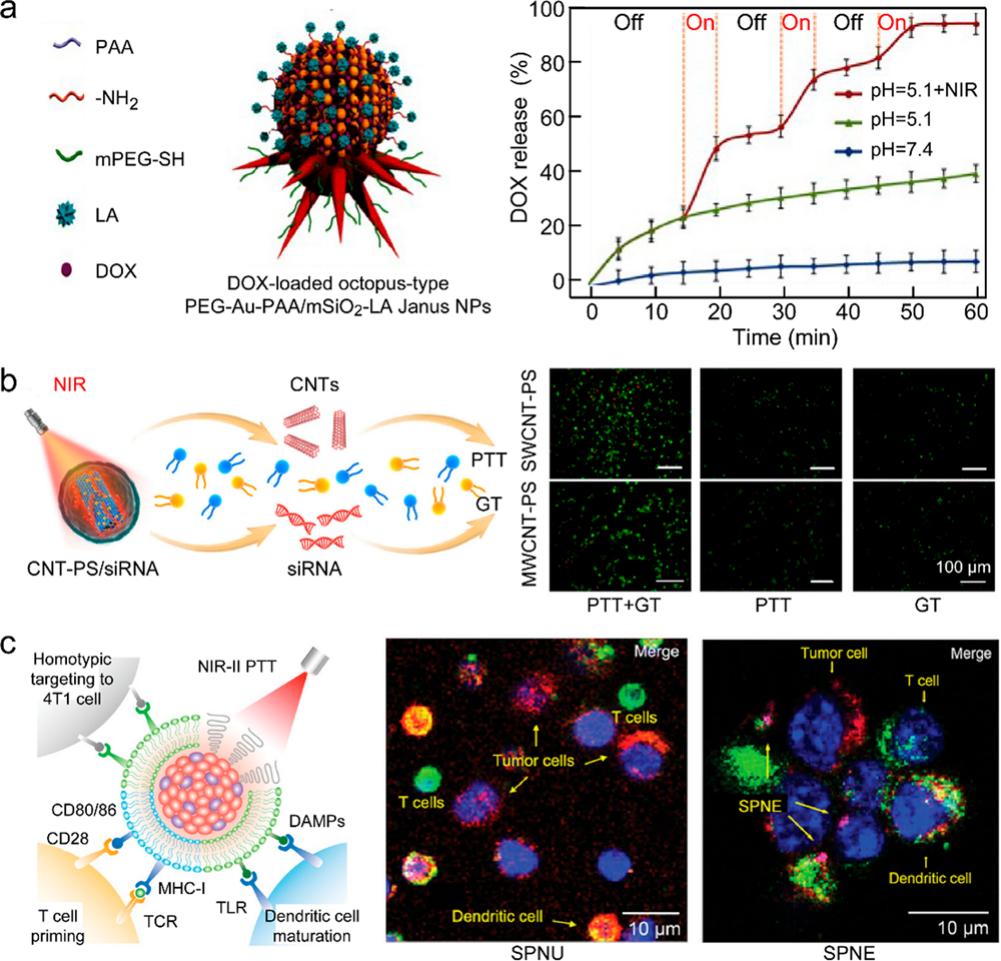
Synergistic cancer treatment based on PTT. (a) Chemo-PTT. Au@PAA Janus nanoparticles are used as the templates to grow a mSiO_2_ shell and Au branches, which are separately modified with methoxy-poly(ethylene glycol)-thiol (mPEG-SH) and lactobionic acid (LA). Left: schematic illustrating the octopus-type PEG-Au-PAA/mSiO_2_-LA Janus nanoparticle. Right: release profiles of the DOX-loaded Janus nanoparticles under different treatments. Reprinted with permission from ref (718). Copyright 2016 Wiley-VCH. (b) Gene-PTT. Peptide lipid and sucrose laurate are used to coat CNTs to form bifunctional delivery systems (denoted as CNT-PS). Left: schematic showing the temperature-sensitive CNT-PS/siRNA nanoparticle for synergistic PTT and GT. Right: qualitative analysis by fluorescence microscopy of apoptotic cells under the combined treatment. Reprinted with permission from ref (313). Copyright 2021 American Chemical Society. (c) Immuno-PTT. Left: schematic illustrating SPNE-mediated multicellular engagement, immune activation, and NIR-II photothermal effects. Middle and right: confocal laser scanning microscopy (CLSM) images of 4T1 cells, T cells, and DCs cocultured with uncoated semiconducting polymer nanoparticles SPNU (middle) or SPNE (right). 4T1 cells and DCs are labeled with DiI (red) and DiO (green) molecular probes, respectively. DCs are stained with phycoerythrin anti-CD80 antibody and fluorescein isothiocyanate anti-CD86 antibody (yellow). Reprinted with permission from ref (703). Copyright 2021 Wiley-VCH.

Apart from the thermal ablation with PTT, an appropriate temperature increase can be beneficial for promoting tissue regeneration, stimulating drug release, improving the drug delivery efficiency, alleviating hypoxia, and other therapeutic activities.^71,707−709^ These advantages further promote PTT to combine with other therapeutic modalities to achieve improved treatment outcomes by an additive or synergistic effect. The combination partners can mutually not only improve the penetration depth of PTT but also augment the antitumor efficacy at lower doses of photothermal agents or lower irradiation laser powers. A number of therapies, such as photodynamic therapy, chemotherapy, radiotherapy, immunotherapy, sonodynamic therapy, chemodynamic therapy, and gene therapy (GT), have been widely incorporated with PTT.^11,710−714^ For example, as the essential therapeutic approach for malignant tumors, conventional chemotherapy often suffers from low drug release, limited dosage, and heavy systemic toxicity.^715^ The combination of photothermal nanomaterials and chemotherapeutic drugs has been utilized to address the mentioned limitations.^716,717^ Uniform Au@poly(acrylic acid) (PAA) nanostructures have been facilely synthesized for actively targeted chemo-PTT (Figure 24a).^718^ Through the significant accumulation of these Janus nanoparticles at the tumor site, the tumor temperature can exceed 60 °C, producing a high tumor suppression of 98%. Apart from active targeting, the release of doxorubicin hydrochloride (DOX) can be greatly enhanced up to 90% due to the remote heat generation. Moreover, the thermal energy can make the chemotherapeutic agents distribute more evenly, leading to better or even complete tumor elimination. Gene-PTT is another important combinational therapeutic modality, where the photothermal heating controls the intracellular gene expression.^668,719^ The photothermal activation of gene delivery can be realized by heating the cell membrane for enhanced endocytosis or cell fusion. CNT-based systems with excellent photothermal and temperature-sensitive properties have been demonstrated for synergistic antitumor activities (Figure 24b).^313^ The temperature-sensitive molecules of peptide lipid and sucrose laurate have been used to functionalize single-walled carbon nanotubes (SWCNTs) and multiwalled carbon nanotubes (MWCNTs) into photoswitchable gene carriers for loading anti-survivin small-interfering ribonucleic acid (siRNA). The increase in temperature can induce the disassembly of these gene carriers and thus facilitate the trapped siRNA to escape from the endosomes. The siRNA release can effectively suppress the tumor growth by the expression of surviving silencing. More than 76% tumor cells are killed during the gene-PTT, while PTT or GT alone only achieves half of the effect as the combinational therapy. In addition to chemo- and gene-PTTs, immuno-PTT is an emerging therapeutic strategy that introduces a systemic anticancer immune response by the photothermal ablation of a targeted tumor.^720−723^ The immunogenetic cell death (ICD) caused by PTT can be beneficial to the eradication of the disseminated disease.^724,725^ A semiconducting polymer nanoengager (SPNE) has been employed for NIR-II immuno-PTT (Figure 24c).^703^ As the coating of the fused membranes of 4T1 tumor cells, the design of SPNE enables multicellular engagements among different cells. Due to the vaccination effects of the cell membranes, SPNE can exert an intrinsic immune response and thus facilitate the activation of T cells and dendritic cells (DCs). Upon NIR-II irradiation, SPNE is driven to directly destroy tumors and induce the ICD of the cancer cells, which can facilitate T cell priming and DC maturation. The populations of the primed T cells and mature DCs are increased by 1.45- and 1.21-fold in primary tumors. The tumor growth is efficiently curbed with the inhibition rate of 97% and eliminated with no recurrence for 30 days. As a result, PTT and its combinational therapies with demonstrated efficacy have been well developed as promising treatment options for various life-threatening diseases.^667,679,726−728^

#### 5.5.2 Drug Delivery

**Figure 25:**
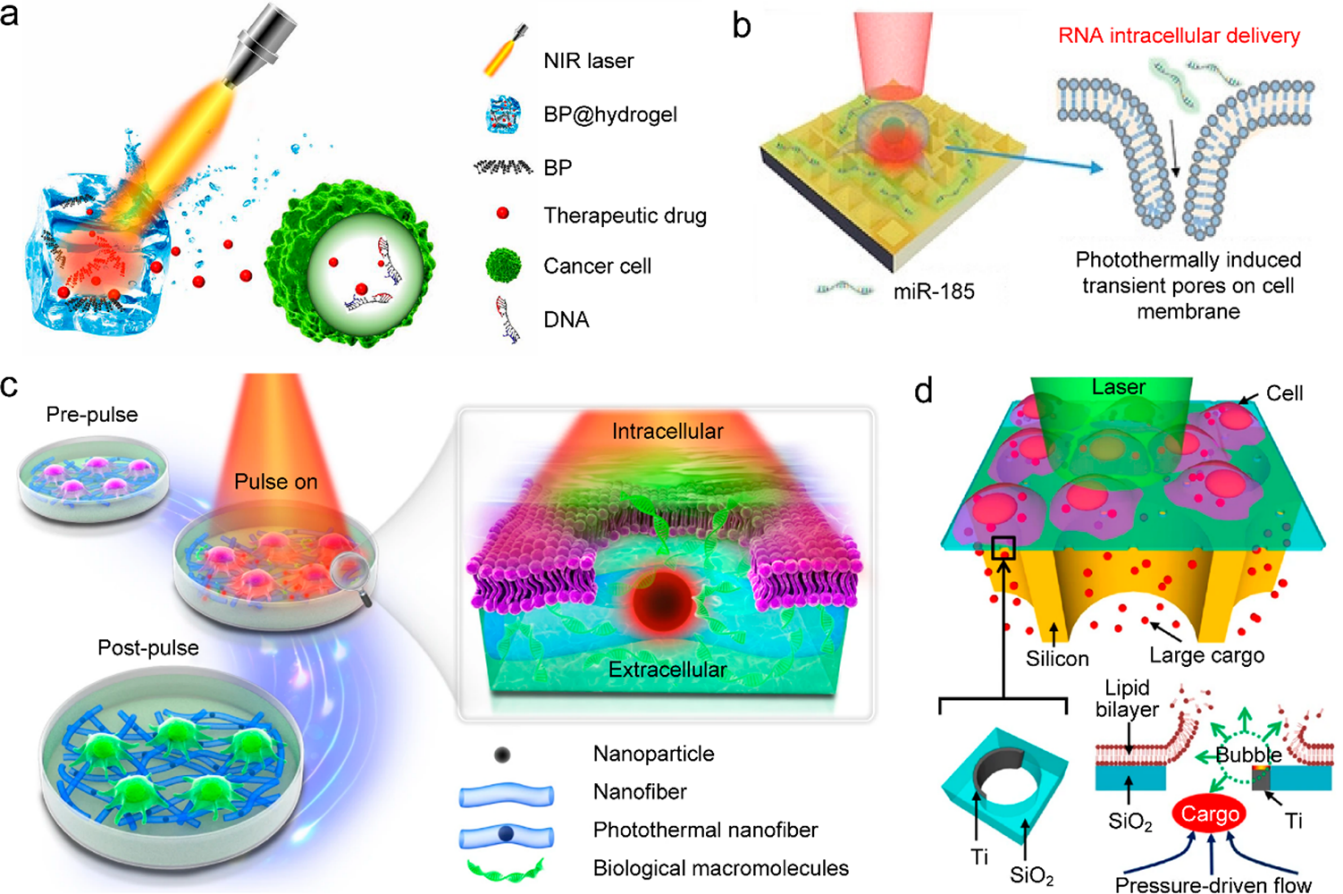
Drug delivery. (a) Schematic showing the release of the encapsulated chemotherapeutic drug caused by NIR light irradiation on BP@hydrogel, resulting in the breaking of the DNA chains and the apoptosis induction. Reprinted with permission from ref (740). Copyright 2018 National Academy of Sciences. (b) Schematic illustrating the intracellular delivery of miRNA by the photothermal heat generated on plasmonic pyramid arrays. Reprinted with permission from ref (756). Copyright 2022 Wiley-VCH. (c) Schematic showing intracellular delivery through membrane permeabilization with photothermal nanofibers. Reprinted with permission from ref (757). Copyright 2021 Springer Nature. (d) Schematic illustrating large-cargo delivery driven by photothermal-heat-triggered cavitation bubbles. Reprinted with permission from ref (758). Copyright 2015 Springer Nature.

Drug delivery is an essential biological process in living tissues because the delivery efficiency and accumulation of therapeutic medicines directly determine the success and failure of a treatment.^729^ Chemotherapy, suffering from insufficient delivery and poor pharmacokinetics of drugs, is one typical representative that often fails in tumor treatments.^730^ The remote heat generation provided by photothermal nanostructures can be utilized to trigger drug release, promote the cellular uptake of drugs, and overcome drug resistance.^731−734^ A variety of photothermal nanomaterials, especially hollow nanostructures and 2D nanosheets with high drug loading efficacy, have been designed as nanocarriers for controllable drug release.^735−739^ A BP-based hydrogel drug delivery system with high therapeutic efficacy has been reported (Figure 25a).^740^ The phase transition from the solid to gel state of BP@hydrogel can be triggered to control the drug release under NIR light irradiation. Owing to the good biocompatibility and biodegradability of BP, the BP@hydrogel nanocarriers are completely degradable and nontoxic. Inorganic nanocarriers generally possess high photothermal conversion efficiencies and high photothermal stability, which are beneficial for drug delivery.^741^ However, many biological barriers are required to be overcome before the nanocarriers enter targeted cells.^742^ The existence of biofilms, which hinder the penetration of nanocarriers and reduce drug susceptibility, is one of the significant barriers for clinical trials.^667^ Various strategies have been explored to enhance the penetration of drug carriers by introducing targeting membranes, changing the protecting ligands/coatings, and optimizing the size and shape of the carriers.^743−747^ Both biodegradability and biocompatibility can be achieved through integration with organic polymers.^748−750^ For monomeric photothermal molecules, supramolecular design offers an excellent opportunity for efficient drug delivery by overcoming their disadvantages of limited accumulation, poor aqueous solubility, and low photothermal conversion efficiencies.^751,752^ Moreover, the strategy for triggering drug release is also an essential part for the design of drug carriers. For the use of photothermal heat as an external stimulus, the drug release can be achieved through the deformation of thermosensitive materials or destabilization of the interaction between carriers and drugs.^753−755^ Apart from the construction of drug carriers, drug delivery can also be realized by a temporal cell-membrane-disruption method (Figure 25b).^756^ The instantaneous high temperature generated by plasmonic pyramid arrays is used to disrupt the cell membranes to promote the intracellular delivery. The membrane permeation is thus increased with the production of transient pores on the cell membranes, which permits the direct diffusion of miRNA molecules. To minimize toxicity to healthy cells, intercellular delivery without direct cellular contact with photothermal nanoparticles has also been demonstrated (Figure 25c).^757^ Although embedded in biocompatible electrospun nanofibers, iron oxide nanoparticles can effectively absorb visible light and transfer efficiently generated heat to distinct places of the cell membrane with improved membrane permeability. When the temperature rapidly increases at the interface between the photothermal nanostructure and the cell membrane due to the photothermal effect, nanobubbles can be formed through the vaporization of the liquid environment (Figure 25d).^758^ The expansion and collapse of the vapor nanobubbles can create high pressure and strong fluid flows, thereby disrupting the nearby membrane and driving the intercellular delivery. The generated vapor nanobubbles can also prevent thermal energy from transferring to the surrounding environment, which is beneficial for better cell viability. This membrane-disruption strategy offers a low-cytotoxicity and high-throughput way to deliver diverse biomolecules and nanoparticles into cells.

#### 5.5.3 Bacterial Inhibition

**Figure 26:**
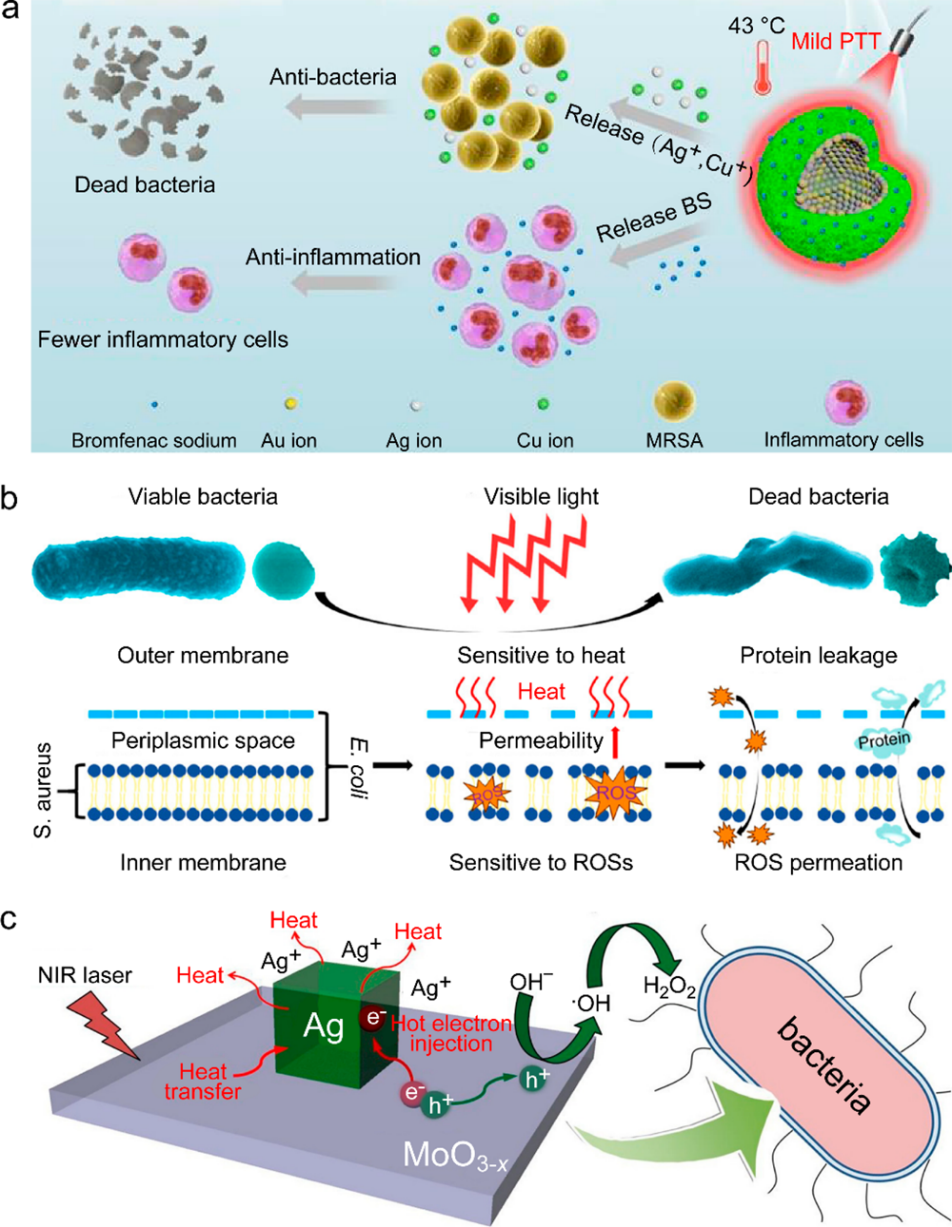
Antibacterial therapy. (a) Schematic illustrating the antibacterial treatment through the release of metal ions (Ag^+^ and Cu^+^) from AuAgCu_2_O-bromfenac sodium nanoparticles. Reprinted with permission from ref (770). Copyright 2020 Ye et al., published under the CC BY 4.0 license http://creativecommons.org/licenses/by/4.0/. (b) Illustration showing the antibacterial mechanism of bacterial membrane disruption with ROSs. Reprinted with permission from ref (774). Copyright 2019 American Chemical Society. (c) Schematic illustrating the photothermal and photocatalytic antibacterial mechanisms of hybrid nanostructures made of MoO_3-*x*_ and Ag. Reprinted with permission from ref (775). Copyright 2018 Elsevier.

The wide antibiotic abuse to combat bacterial infections has brought about a series of severe healthcare problems in our daily life. The bacterial resistance caused by antibiotics and the formation of self-protected biofilms make bacterial inhibition a global health task.^759,760^ The development of photothermal materials offers a promising way for addressing antibacterial challenges.^327,761,762^ Owing to the photothermal effect, the generated hyperthermia can destroy the integrity of pathogenic bacteria. Unlike the response to antibiotics, bacteria under photothermal treatment have difficulty producing drug resistance and adverse effects.^381^ The protective matrix of bacterial cells, including proteins and nucleic acids, can be inactivated or damaged by localized heating,^763,764^ and thus antibacterial drugs find it much easier to penetrate the biofilm and kill the targeted bacteria. This bactericidal treatment with hyperthermia generally requires high power of light irradiation and high dosage of photothermal agents. Other bactericidal strategies have also been investigated to incorporate with the photothermal effect to achieve better curative efficacy.^765^ The introduction of toxic metal ions such as Ag^+^, Cu^2+^, As^3+^, and Hg^2+^ can facilitate the bactericidal activities by coordinating with the biological ligands of bacteria.^766,767^ The formation of metal–biomolecule complexes can thus destroy the structures of DNAs, membranes, and enzymes, resulting in the death of bacterial cells.^768,769^ Hollow AuAgCu_2_O-bromfenac sodium nanostructures have been designed as bifunctional carriers for both antibacterial and anti-inflammatory treatments (Figure 26a).^770^ When the release of Ag^+^ and Cu^2+^ is mediated by a mild photothermal effect for bacterial elimination, the anti-inflammatory drug can also be released to facilitate tissue rehabilitation. The participating Cu^2+^ not only acts as a bactericidal agent but also promotes the healing of the infected wounds. Another important strategy for the bactericidal activity is to generate reactive oxygen species (ROSs) through a photodynamic or photocatalytic process.^771−773^ ROSs, including singlet molecular oxygen, hydroxyl radicals, and superoxide anions, can cause autophagy, apoptosis, and necrosis of bacterial cells.^774^ The synergistic combination of photothermal and photodynamic effects has been demonstrated to enhance bacterial membrane permeation (Figure 26b).^774^ The outer and inner bacterial membranes are disrupted by heat and ROSs, respectively. The entry of ROSs in bacteria can further induce the outbreak of oxidative stress and the protein leakage, leading to a fast and effective bactericidal activity. A plasmonic hybrid system made of MoO_3-*x*_ and Ag has been reported for enhanced bacterial killing with synergistic photothermal and photocatalytic effects (Figure 26c).^775^ MoO_3-*x*_ can efficiently convert the absorbed NIR light into heat, and further transfer the thermal energy to the Ag nanocubes to drive the release of Ag^+^. The hot electrons and holes can be produced and separated at the interface between MoO_3-*x*_ and Ag, promoting the generation of ROSs. The bactericidal activity is greatly enhanced in the cooperation of thermal energy, Ag^+^, and ROSs.

#### 5.5.4 Polymerase Chain Reaction (PCR)

**Figure 27:**
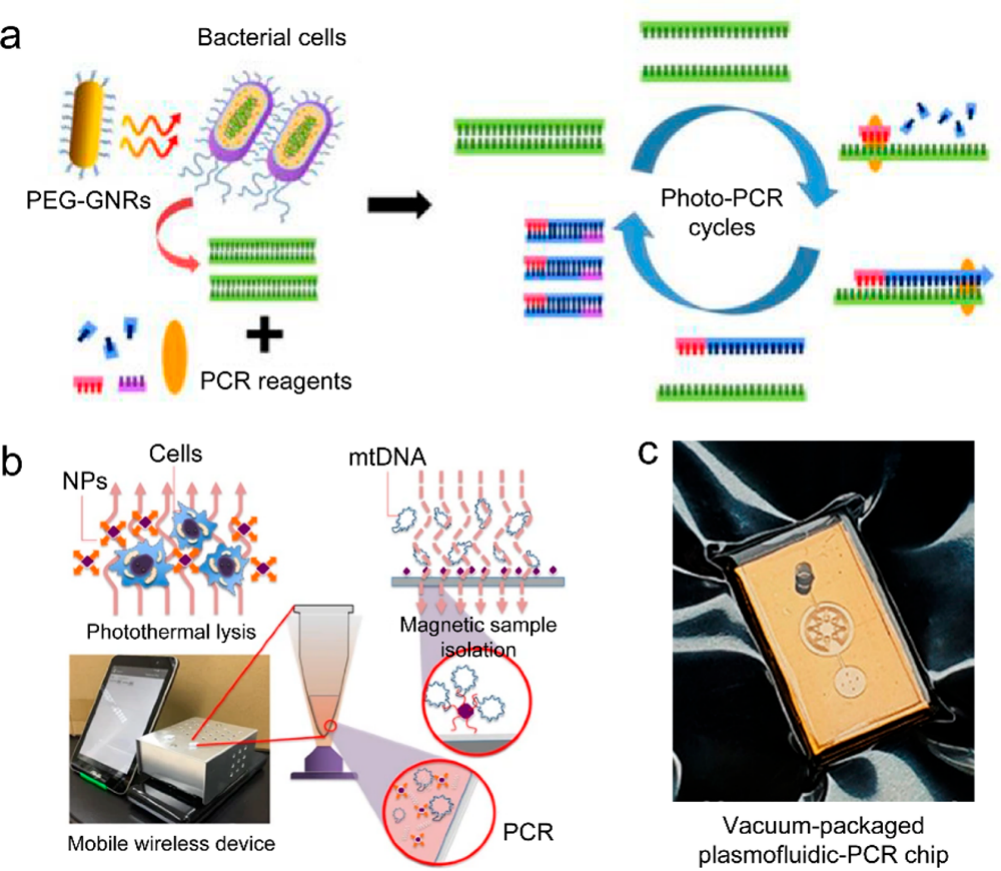
Polymerase chain reaction. (a) Schematic showing DNA extraction and the photo-PCR of bacterial cells. Reprinted with permission from ref (780). Copyright 2017 Ivyspring International Publisher under the CC BY 4.0 license http://creativecommons.org/licenses/by/4.0/. (b) Schematic depicting the real-time PCR using dual-modal magnetic Fe_3_O_4_ nanoparticles for DNA isolation and amplification. Reprinted with permission from ref (782). Copyright 2016 Springer Nature under the CC BY 4.0 license http://creativecommons.org/licenses/by/4.0/. (c) Photograph of the plasmofluidic-PCR chip. Reprinted with permission from ref (75). Copyright 2021 Kang et al., published under the CC BY 4.0 license http://creativecommons.org/licenses/by/4.0/.

PCR has been the standard molecular diagnostic routine for amplifying and detecting nucleic acids because of its high sensitivity and specificity. PCR mainly relies on a three-step thermal cycling, including (1) denaturation—separation of two strands of DNAs at a high temperature; (2) annealing—hybridization of the complementary and short single-stranded DNA fragments at a low temperature; and (3) extension—generation of a new DNA strand from the annealed DNA template by polymerase.^776^ Such a chain reaction process allows researchers to replicate billions of specific nucleic acid segments exponentially. The initial nucleic acid concentration can also be estimated according to the cycle threshold value. PCR has therefore been regarded as the gold standard for quantitative diagnosis. However, many conventional PCR-based bioassays are limited by time-consuming procedures and expensive Peltier-based heating blocks.^74^ Since multiple cycles and two or three discrete temperatures are required for the amplification of nucleic acids, to reduce the thermocycling rate has become the key to achieving fast/ultrafast PCR. Nanomaterials with excellent photothermal capabilities have been explored to accelerate heating and cooling processes through volumetric heating and efficient heat transfer.^76,777−779^ A Au-nanorod-based PCR system has been reported for the rapid detection of bacterial cells (Figure 27a).^780^ The procedures of the bacteria lysis and DNA amplification are integrated into one step to reduce the cycling time. The total time for the complete identification of the bacterial DNAs is shortened to less than 20 min. By optimizing the structural design of nanomaterials for higher photothermal conversion efficiencies, the thermocycling time can be further reduced to achieve the desirable ultrafast PCR.^77,781^ Dual-modal Fe_3_O_4_ nanoparticles with both photothermal and magnetic properties have been used to develop a portable real-time PCR device (Figure 27b).^782^ The magnetic Fe_3_O_4_ nanoparticles are employed to promote the movements of the primers and DNA templates and thus accelerate the DNA amplification. Compared to plasmonic metals, Fe_3_O_4_ possesses better thermal stability. It can resist high temperatures. Furthermore, an ultrafast vacuum-packaged plasmofluidic PCR chip has been developed for highly efficient and small-volume bioassay (Figure 27c).^75^ The amplification process with 40 cycles can be completed in 306 s for SARS-CoV-2 and in 264 s for λ-DNA, together with an amplification efficiency of 91%. In a recent PCR example, the nucleic acid amplification can be reduced to 4 cycles with a runtime of 7.5 min for the detection of SARS-CoV-2.^788^ PCR-based tests have been the gold standard with a high analytical accuracy of 99% for COVID-19 confirmation.^783,787^ Although the PCR technology has been already commercialized for rapid diagnosis, particularly for COVID-19, the investigation of nanomaterials with better photothermal performances is still an essential pathway for the development of ultrafast, robust, and portable PCR at the point-of-care level.^783,784^

#### 5.5.5 Fight against COVID-19

**Figure 28:**
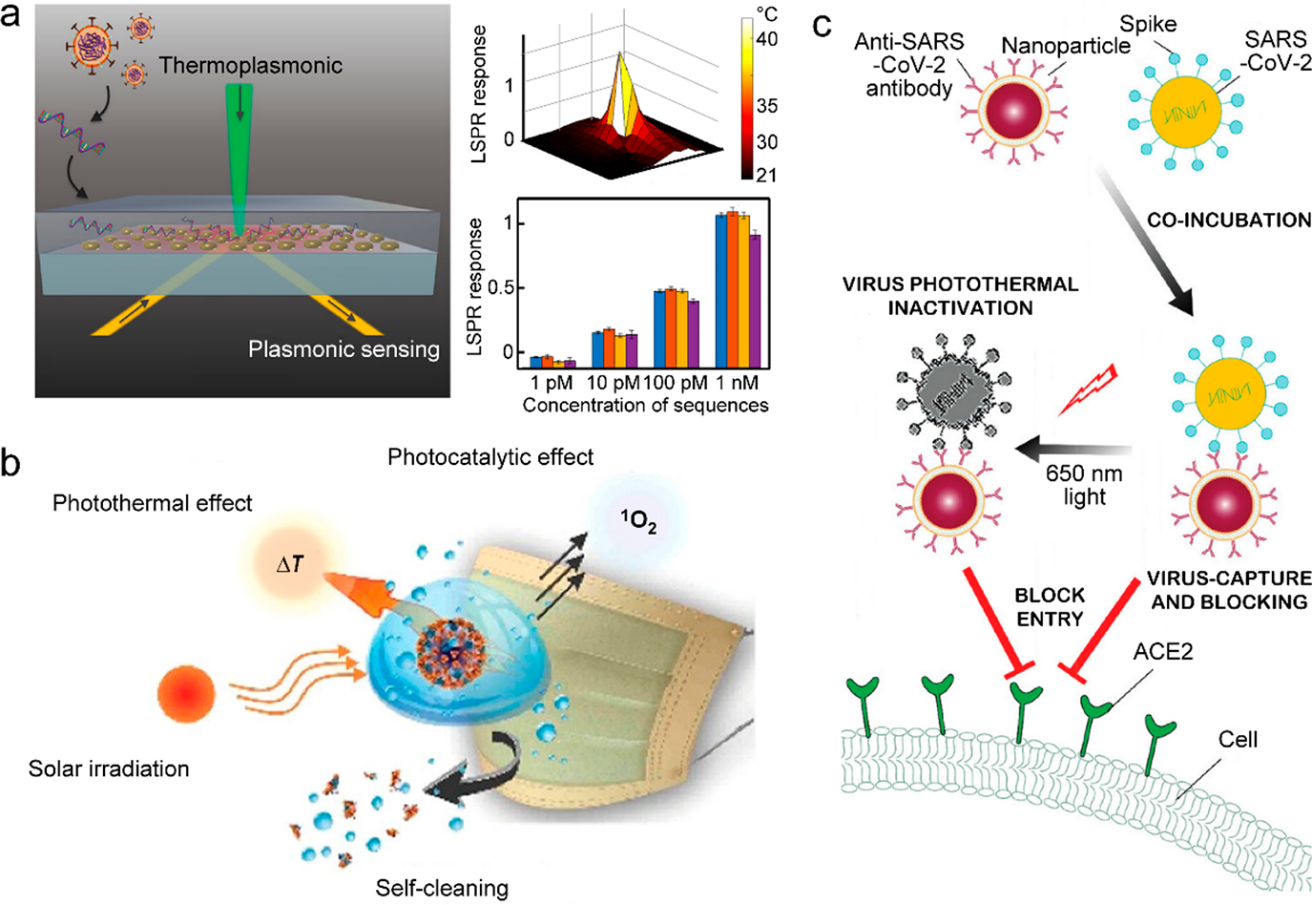
Applications of photothermal nanomaterials in COVID-19 diagnosis, prevention, and treatment. (a) Left: schematic showing photothermally enhanced LSPR sensing. Right: temperature distribution map around the heat source on a Au nanoisland (top) and concentrations of various viral oligos measured with the dual-functional LSPR biosensor (bottom). Reprinted with permission from ref (80). Copyright 2020 American Chemical Society. (b) Schematic illustrating the inactivation of the virus in a nonwoven surgical mask through photothermal, photocatalytic, and hydrophobic self-cleaning processes. Reprinted with permission from ref (789). Copyright 2021 American Chemical Society. (c) Schematic illustrating a multifunctional photothermal nanoparticle for the capture (by antibody) and inactivation (by photothermal conversion) of SARS-CoV-2. Reprinted with permission from ref (791). Copyright 2022 Cai et al., published under the CC BY 4.0 license http://creativecommons.org/licenses/by/4.0/.

The outbreak of COVID-19, resulting from the infection of severe acute respiratory syndrome coronavirus 2 (SARS-CoV-2), has brought about a global pandemic to threaten public health. Scientists around the world have made great progresses in personal protective equipment, effective vaccines, reliable diagnostic tools, and therapeutic approaches.^78,785,786^ Photothermal nanomaterials can also pose a significant impact on the processes of diagnosis, prevention, and treatment to fight against COVID-19. Apart from the PCR-based test, a dual-functional plasmonic biosensor based on the photothermal effect and LSPR sensing has been developed with a highly sensitive and reliable detection capability for COVID-19 diagnosis (Figure 28a).^80^ The localized photothermal heat is employed to elevate the hybridization temperature of the nucleic acids and improve the separation accuracy of two similar gene segments. The detection limit for the selected SARS-CoV-2 sequences can be lowered to a concentration of 0.22 pM. Respiratory face masks can also be integrated with photothermal nanomaterials for antimicrobial action (Figure 28b).^789^ The synergistic combination of photothermal and photocatalytic effects can endow the masks with reusability and a self-sterilizing ability. More types of reusable and antimicrobial face masks have also been demonstrated in cooperation with different photothermal nanomaterials.^82,790^ Furthermore, the decoration of photothermal nanoparticles with high-affinity neutralizing antibodies, which are specific to the spike proteins of SARS-CoV-2, has been designed to effectively capture the virus (Figure 28c).^791^ SARS-CoV-2 is forbidden to enter host cells due to the antibody treatment and subsequently inactivated owing to the photothermal effect. This strategy of functionalizing photothermal nanoparticles with antiviral agents offers tremendous potential for blocking SARS-CoV-2 and reducing the infection.^78,106^

### 5.6 Other Applications

Besides the above-discussed applications, photothermal nanomaterials can also be potentially applied in sensing, wearable devices, energy storage and conversion, as well as photothermal electrodes. In this section, several representative examples of these applications will be presented.

**Figure 29:**
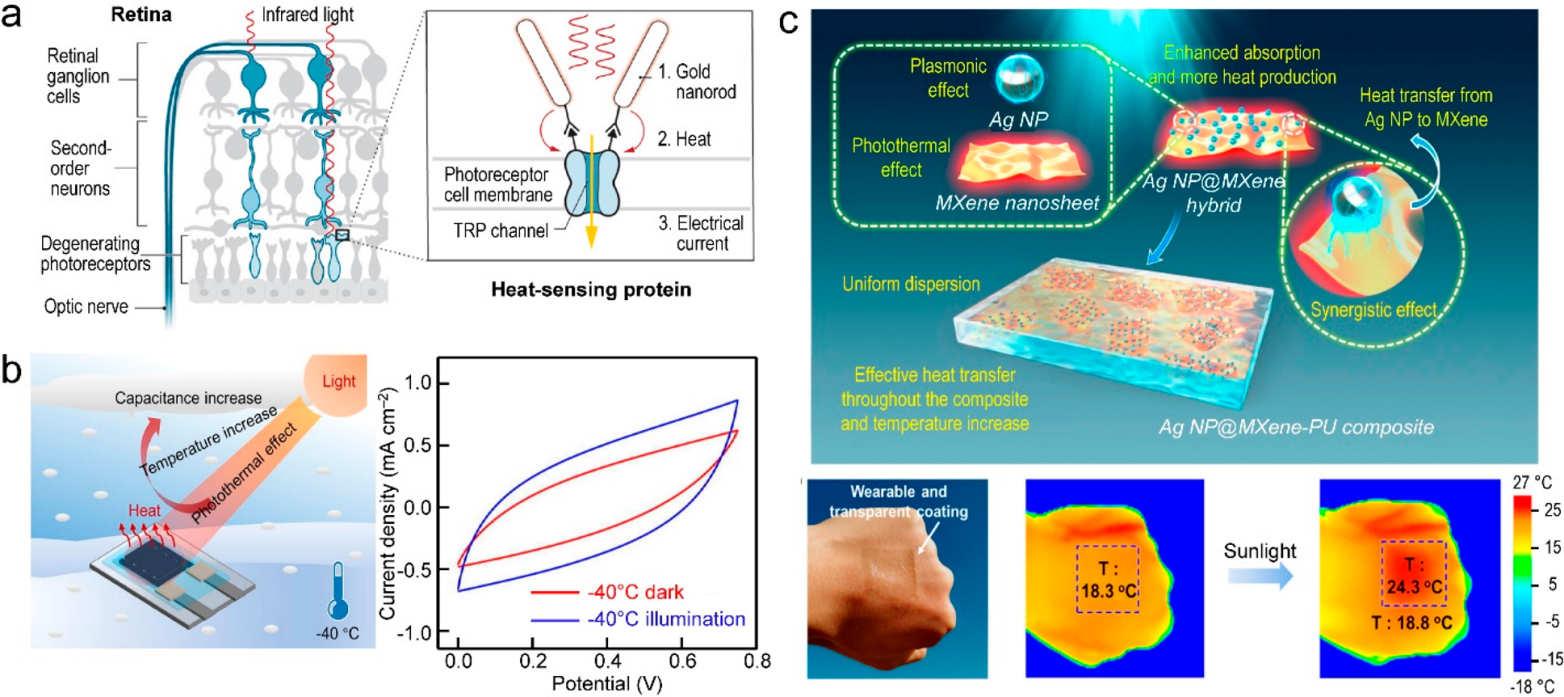
Representative potential applications of photothermal nanomaterials in sensing, photothermal electrodes, and wearable devices. (a) Sensing. The schematic illustrates the restoration of the light sensitivity of an eye using Au nanorods bound to temperature-sensitive engineered TRP channels. Reprinted with permission from ref (792). Copyright 2020 American Association for the Advancement of Science. (b) Photothermal electrodes. Left: schematic illustrating a TiN-based supercapacitor under solar illumination at an open environment and −40 °C. Right: cyclic voltammetry curves of the photothermally assisted supercapacitor at −40 °C with and without solar illumination. Reprinted with permission from ref (797). Copyright 2021 Elsevier. (c) Wearable devices. Top: schematic illustrating the photothermal effect produced from (Ag nanoparticle)@MXene hybrid structures when irradiated with light. Bottom: photograph (left) of a composite film made of (Ag nanoparticle)@MXene and polyurethane and attached on one human hand, and IR thermal images of the human hand with the attached film before (middle) and after (right) solar irradiation for 1 min. Reprinted with permission from ref (801). Copyright 2019 American Chemical Society.

Photothermal nanomaterials are normally hybridized with some other temperature-sensitive components to achieve sensing functionality. In the hybrid system, photothermal nanomaterials function as light-to-heat converters that will transfer the converted thermal energy to the temperature-sensitive components. The temperature-sensitive components further transform heat into other types of signals, for instance, electrical changes, which are easily detected and thus realize the sensing function. For example, Au nanorods have been used as antennae for IR light, transforming light into heat in photoreceptors that are the light-sensing cells in the eye (Figure 29a).^792,793^ Au nanorods can capture NIR light at their resonance wavelengths to generate heat, which is harnessed to open temperature-sensitive transient receptor potential (TRP) channels. Antibodies are used to bind Au nanorods to the heat-sensitive TRP channels. Combined with GT, such a dual system can enable the activation of photoreceptors by IR light and thus be potentially used to restore light sensitivity in a blind human retina.

The heat generated by photothermal nanomaterials under light irradiation has been widely used to accelerate chemical reactions and enable reactions that require harsh conditions to proceed under mild conditions. Photothermal nanomaterials can consequently be employed as electrode materials for electrochemical reactions, which will enhance electrocatalytic reactions^794−796^ or allow reactions to proceed in cold atmosphere.^797^ Take photothermal supercapacitors as an example (Figure 29b, left panel).^797^ A photothermal supercapacitor has been designed using an electrochemically active and photothermal electrode material. It shows the capability of operation even under −40 °C. The used commercially available TiN nanocrystals exhibit broad light absorption (>98%) in the entire solar spectrum and a photothermal conversion efficiency of 62.5%, endowing the as-fabricated device with a rising temperature of 25.8 °C under solar light illumination. Such a photothermal effect further contributes to the restored capacitance of the device from a declined one at −40 °C (Figure 29b, right panel). The capacitance and energy density of the device at −40 °C are restored to be 70.9% and 59.3%, respectively, of those at 25 °C when under solar light illumination.

Wearable devices have emerged as a hot research topic in recent years due to their great potential in human healthcare and health monitoring.^798−800^ Specifically, photothermal nanomaterials provide wearable devices with multiple functions including PTT, self-healing, EMI shielding, human physiological signal monitoring, and energy harvesting.^801−807^ We focus here on the discussion of the functions of nanomaterials introduced by the photothermal effect, among which PTT has been described in detail in Section 5.5.1. We therefore discuss here the representative examples of how photothermal nanomaterials work in the self-healing of wearable devices, as shown in Figure 29c and in energy harvesting.

Wearable devices can suffer from unexpected mechanical damages during practical applications because the real world is dynamic rather than static. Silver-nanoparticle-anchored MXene nanohybrids have been designed to achieve a rapid and *in situ* repair of the damaged elastic polyurethane. The nanohybrids have been incorporated into a polyurethane elastomer through solution-based blending and coating processes. The synergistic effects from the photothermal conversion of the MXene nanosheets and the plasmonic effect of the Ag nanoparticles lead to a temperature increase of approximately 111 °C under light irradiation for 5 min (Figure 29c, top panel). Such an increased temperature results in the fusion of the elastic polyurethane and consequently the repair of the damaged composite. A healing efficiency of 97% has been achieved.

It is also worth mentioning that when integrated with thermoelectric materials, photothermal nanomaterials can potentially harvest solar energy and transfer it to thermal energy, which is further harnessed to generate electricity to power wearable devices.^806,807^ For instance, PPy, a photothermal layer, has been deposited on a thermoelectric layer (poly(3,4-ethylenedioxythiophene):tosylate (PEDOT:Tos)) to achieve photothermoelectric generation for wearable devices.^803^ Taking advantage of the efficient photothermal conversion of PPy, the as-fabricated photothermoelectric fabric can generate a higher voltage of 536.47 μV than that without the PPy coating (294.13 μV) under IR light irradiation, giving a power density up to 13.76 nW m^–2^.

Phase-change materials provide one promising way to store solar thermal energy because of their high phase-change latent heat.^808^ The key factors in determining the photothermal energy conversion efficiency include the capability of solar-thermal conversion, thermal stability, shape stability, thermal conductivity, and thermal energy storage density.^809−813^ Even though conventional phase-change materials, such as paraffin waxes and fatty acids, exhibit high energy-storage capacities, they generally own limitations in photothermal energy conversion, thermal conductivity and stability. They therefore exhibit low photothermal energy conversion efficiencies, which limits their applications in solar energy conversion. Photothermal nanomaterials have become promising for effectively addressing the above-mentioned challenges through incorporation with phase-change materials. Different types of photothermal nanomaterials, including carbon-based nanomaterials (graphene oxide, CNTs),^811,814,815^ MXene nanosheets,^813,816−818^ metal nanoparticles,^810,819^ and organic polymers,^820−822^ have been successfully combined with phase-change materials to achieve high photothermal energy conversion efficiencies. For example, a layered phase-change composite made of polyethylene glycol and Ti_3_C_2_T_*x*_ has been demonstrated to show an improved photo-to-thermal storage efficiency of 94.5% under natural solar light irradiation, which is higher than that of pure polyethylene glycol.^813^ Besides the photothermal storage efficiency, the as-fabricated composite also presents a high energy density and stable property before and after the phase change.

## 6 Conclusion and Outlook

The recent advances on photothermal nanomaterials have been comprehensively summarized in this review article, including their photothermal conversion mechanisms, material choices, temperature measurements, and diverse applications. One major advantage of photothermal nanomaterials is their broad light absorption range and excellent photothermal conversion ability, thus allowing for the efficient utilization of solar energy as a sustainable solution for energy scarcity. In addition to acting as an energy source, light can also serve as an external control to remotely alter thermal gradients, as well as the position and morphology of photothermal nanoparticles. Through further integration with other molecules or particles, the functionalized photothermal nanomaterials with synergistic properties can perform multiple tasks for targeted applications, including light manipulation, catalysis, and therapy, with the assistance of thermal energy.

Although the generation of photothermal heat can be divided into the three different mechanisms, which are plasmonic localized heating, nonradiative relaxation, and thermal vibrations, it has still been difficult to unambiguously identify the photothermal conversion mechanism of a given type of nanostructure among various newly emerging materials and to choose proper nanomaterials for specific applications. To further improve the performance of photothermal materials and extend their applications to more scenarios, many challenges will need to be overcome in the near future. The synthesis of nanomaterials with extremely high absorption over a broad wavelength range has always become a very active field, attracting more and more research interests toward perfect light absorbers.^15,18^ Further development of economical approaches for the mass production of photothermal materials is essential for realizing their ultimate usage in daily life and industrial applications. Moreover, how to maintain the stability and photothermal conversion efficiencies of nanomaterials during long-term operation has still remained a challenge for practical applications and requires further exploration.^22,27,28^

The implementation of many photothermal applications requires the accurate monitoring of local temperatures, such as PTT,^20,24,71^ biomedical diagnostics,^385,386^ photochemistry,^9,387^ nanofabrication,^64,85,519^ and realistic microelectronic devices.^798,800^ Regardless of the ultimate application, there is still much room for the development of new nanothermometry with high sensitivity, accuracy, spectral/temporal resolution, reproducibility, stability, biocompatibility, cost effectiveness, and ease of operation. The heater–thermometer nanoplatforms reported so far have shown the possibility of simultaneous photothermal heating and real-time temperature feedback, which is particularly attractive for controllable biomedical therapy and photothermal catalysis. On the one hand, biological applications of heater–thermometer nanoplatforms not only require biocompatibility but also place additional demands on the optical properties that allow the heating beam, the fluorescence excitation beam, and the emission beam to pass through the involved tissues.^385^ On the other hand, an ideal tool is also highly demanded for monitoring photocatalytic processes. Even though thermal energy has been utilized in many works to drive or assist in catalytic chemical reactions, it has remained challenging to clearly distinguish the contributions of photothermal heating and nonthermalized hot charge carriers. Debates have lasted for many years on the dominant effect in plasmon-enhanced catalysis, where both hot charge carriers and thermal energy can be produced through the excitation of LSPRs.^653−657^ As these two effects occur at different time scales and are originated from different physical processes, advanced technologies and ingeniously designed experiments are required to uncouple the underlying mechanisms of plasmon-enhanced catalysis and thus realize the use of plasmons to better control chemical reactions in terms of the reaction pathway, activity, and selectivity.

For solar thermal water heating and evaporation, a standardized process for evaluating the performance of different photothermal nanomaterials is still lacking.^53,507^ Researchers are suggested to list the detailed measurement conditions and calculation methods for the evaluation of the evaporation efficiency of the studied photothermal system. A widely discussed guideline by researchers in this field to fairly compare different photothermal nanomaterials will be highly desired. Further studies on water desalination and purification should also consider volatile organic compounds, some of which can be vaporized and collected with desalinated water.^53^ The development of multifunctional nanomaterials with the capabilities of photothermal conversion and the removal of volatile organic compounds can be a solution. For structural color printing, we envision that further research should be devoted to the development of efficient techniques for tuning the colors of individual high-resolution pixels toward dynamic color display.^62,508^ Beyond the control of the reflection, transmission, and scattering spectra of light in structural color prints, localized laser heating can also be used to fabricate metasurfaces and metamaterials for the adjustment of the phase, polarization, and angular momentum of light.^525,590^ The merits of light-driving nanomotors, actuators, and robots include the remote energy supply and multiple tuning parameters of light for photothermal manipulation. However, most research works on photothermal manipulation have only demonstrated simple motions of micro-/nano-objects. More efforts are needed toward the design and construction of mature systems that can accomplish complicated tasks. The biomedical applications of photothermal manipulation for in-body treatment and surgery as well as precise manipulation of biological components are still challenging.^616^ Biocompatible and biodegradable nanoscale motors, actuators, and robots, which can be operated under safe stimulating conditions, are required. Additional efforts are also desired for reducing the response time during in-body operation. We are confident that the more elegant integration of photothermal nanomaterials with thermal responsive components will produce smarter nanomotors, actuators, and robots for wider applications in engineering and medicine.

Photothermal nanomaterials have been widely studied in the field of life sciences and especially show great potential for PTT. There are still many problems to be addressed. High water solubility, good biocompatibility and biodegradability are desired for inorganic photothermal nanomaterials to provide low toxicity.^98,645^ Organic nanomaterials are expected to have high thermal stability and high photothermal conversion efficiencies. The development of nanomaterials with strong light absorption in the biological NIR-II window is required to achieve deeper tissue penetration for enhanced photothermal therapeutic efficacy. It is also necessary to quantify the damage caused by the hyperthermia on the surrounding tissues and minimize the injury of normal cells.^381^ Moreover, much effort should be made to integrate photothermal nanomaterials with biologically mimic coatings to overcome different biological barriers and thus facilitate drug delivery, bacteria inhibition, and other biological activities.^742^

Nowadays, most of the aforementioned photothermal nanomaterials and nanothermometers are commercially available as raw materials for researchers and start-up companies. While most applications have only been demonstrated in research laboratories, a few examples have shown promise for real-life applications, such as the PCR test, AuroLase Therapy, and wearable devices. Furthermore, there is great potential of photothermal nanomaterials in energy science in terms of solar cells, photothermal catalysis, and energy storage. In general, there exist several critical factors for successful commercialization. First, the ability to scale up the production of diverse photothermal nanomaterials with optimized cost efficiencies is essential for most industrial applications. Second, the biosecurity and environmental safety of these materials have not been fully assessed, which is a critical consideration for commercialization.

We believe that there will be ceaselessly enthusiastic research effort to continuously broaden the family of photothermal nanomaterials and explore new directions for real-life practical applications. The ongoing progresses will undoubtedly address most unresolved challenges and bring about new scientific inquiries. All of the effort will make photothermal nanomaterials an even more powerful light-to-heat converter.

## Abbreviations

0D: zero-dimensional
1D: one-dimensional
2D: two-dimensional
3D: three-dimensional
AIE: aggregation-induced emission
BP: black phosphorus
CLSM: confocal laser scanning microscopy
CNTs: carbon nanotubes
CNT-PS: carbon nanotube coated with peptide lipid and sucrose laurate
COFs: covalent organic frameworks
COVID-19: coronavirus disease 2019
D-A: donor–acceptor
DC: dendritic cell
DOX: doxorubicin hydrochloride
DPP: diketopyrrolopyrrole
EM: electromagnetic
EMI: electromagnetic interference
EPA: Environmental Protection Agency
EXAFS: extended X-ray absorption fine structure spectroscopy
FIR: far-infrared
GT: gene therapy
HOMO: highest-occupied molecular orbital
ICD: immunogenetic cell death
IR: infrared
LA: lactobionic acid
LCN: liquid crystalline network
LSPR: localized surface plasmon resonance
LUMO: lowest-unoccupied molecular orbital
MWCNTs: multiwalled carbon nanotubes
MOFs: metal–organic frameworks
mPEG-SH: methoxy-poly(ethylene glycol)-thiol
mSiO2: mesoporous silica
MXene: two-dimensional transition metal carbide/nitride
NHE: normal hydrogen electrode
NIR: near-infrared
ODMR: optically detected magnetic resonance
PAA: poly(acrylic acid)
PAn: polyaniline
PCC: polydimethylsiloxane/carbon nanotubes/cellulose nanocrystals
PCR: polymerase chain reaction
PDA: polydopamine
PDMS: poly(dimethylsiloxane)
PPy: polypyrrole
PT: polythiophene
PTT: photothermal therapy
PTTe: poly[2,2″-((E)-4,4″-bis(2-octyldodecyl)-[6,6″-bithieno[3,2-b]pyrrolylidene]-5,5″-(4H,4′H)-dione)-alt-2,5-(tellurophene)]
QDs: quantum dots
ROSs: reactive oxygen species
SARS-CoV-2: severe acute respiratory syndrome coronavirus 2
SWCNTs: single-walled carbon nanotubes
SEM: scanning electron microscopy
siRNAs: small-interfering ribonucleic acids
SPNE: semiconducting polymer nanoengager
SPNU: uncoated semiconducting polymer nanoparticles
TEM: transmission electron microscopy
TMDCs: transition metal dichalcogenides
TRP: transient receptor potential
UCL: up-conversion luminescence
UCNPs: up-conversion nanoparticles
WHO: World Health Organization
ZIF-8: zeolitic imidazolate framework-8
